# DCAF12 Ubiquitin Ligase Promotes Lung Cancer Metastasis by Modulating the TRiC/CCT Chaperonin Complex

**DOI:** 10.1002/advs.202509695

**Published:** 2025-10-05

**Authors:** Zhenyi Wang, Huanhuan Huang, Kaizong Huang, Xiaowen Cui, Leilei Wu, Renyu Lin, Zhe Zhao, Hua Chen, Cheng Zheng, Weilin Jin, Song Chen, Jiayan Chen, Yaping Xu, Dongping Wei

**Affiliations:** ^1^ Medical Research Center The First Affiliated Hospital of Wenzhou Medical University Wenzhou Zhejiang 325015 China; ^2^ Department of Oncology Nanjing First Hospital Nanjing Medical University Nanjing Jiangsu 210006 China; ^3^ Department of Clinical Pharmacology Lab Nanjing First Hospital Nanjing Medical University Nanjing Jiangsu 210006 China; ^4^ Department of Radiation Oncology Shanghai Pulmonary Hospital School of Medicine Tongji University Shanghai 200433 China; ^5^ Department of Otolaryngology The First Affiliated Hospital of Wenzhou Medical University Wenzhou Zhejiang 325015 China; ^6^ Xuyi People's Hospital Kangda College of Nanjing Medical University Huai'an Jiangsu 211700 China; ^7^ Department of Thoracic Surgery The First Affiliated Hospital of Wenzhou Medical University Wenzhou Zhejiang 325015 China; ^8^ Institute of Cancer Neuroscience Medical Frontier Innovation Research Center The First Hospital of Lanzhou University The First Clinical Medical College of Lanzhou University Lanzhou Gansu 730000 China; ^9^ Institute of Medicinal Biotechnology Jiangsu College of Nursing Huai'an Jiangsu 223300 China; ^10^ Translational Research Institute of Henan Provincial People's Hospital and People's Hospital of Zhengzhou University Academy of Medical Sciences Zhengzhou University Zhengzhou Henan 450053 China; ^11^ Department of Radiation Oncology Fudan University Shanghai Cancer Center Shanghai 200032 China

**Keywords:** DCAF12, metastasis, proteostasis, TRiC/CCT complex, ubiquitination

## Abstract

Metastasis is the primary challenge in lung cancer treatment. Although proteostasis supports tumor growth, the mechanism by which ubiquitin ligases reprogram chaperone networks to drive metastasis is poorly understood. In this study, it is revealed that DDB1‐CUL4‐associated factor (DCAF12), a substrate receptor for CUL4‐RING ubiquitin ligases, regulates metastatic progression through ubiquitin‐mediated proteostatic reprogramming. DCAF12 depletion suppresses tumor cell migration and stemness in vitro and reduces pulmonary/hepatic metastasis in vivo. Mechanistically, DCAF12 catalyzes the non‐degradative ubiquitination of TRiC/CCT subunits, enhancing chaperonin assembly and folding of cytoskeletal effectors (β‐actin/tubulin) and oncogenic clients (STAT3/Raptor/mLST8), thereby activating the YAP, STAT3, and mTOR pathways. Both genetic knockdown and pharmacological blockade (via HSF1A) of this axis potently inhibit metastasis. Clinically, DCAF12 overexpression is correlated with YAP/STAT3 activation, advanced metastasis, and poor survival. Three key insights are revealed: 1) ubiquitination‐mediated TRiC/CCT regulation as a metastatic switch, 2) DCAF12 as an oncogenic proteostasis hub, and 3) therapeutic potential validated through multimodal targeting. These findings establish the DCAF12‐TRiC/CCT axis as a mechanistically novel target that simultaneously disrupts cytoskeletal dynamics and oncogenic signaling, making it a promising therapeutic strategy for metastatic lung cancer.

## Graphical abstract

This study elucidates how DCAF12 facilitates non‐degradative ubiquitination to stabilize TRiC/CCT, thereby enhancing the folding capacity of chaperonins. This mechanism promotes the maturation of cytoskeletal proteins and activates key oncogenic drivers, including YAP, STAT3, and mTOR, ultimately driving metastatic progression in lung cancer. These findings underscore the therapeutic potential of targeting this axis using the TRiC/CCT inhibitor HSF1A.

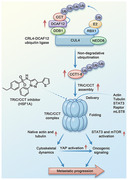

## 1 Introduction

Metastasis accounts for the majority of cancer‐related deaths, with non‐small cell lung cancer (NSCLC) representing a particularly aggressive form of disseminated disease.^[^ ^1^, ^2^ ^]^ Although immune checkpoint inhibitors (e.g., anti‐PD‐1/PD‐L1 therapy) and targeted therapies have expanded treatment options, metastatic NSCLC maintains a poor 5‐year survival rate of <10%,^[^ ^2^, ^3^ ^]^ underscoring the need to identify the novel drivers of metastasis.

Tumor progression is sustained by proteostasis in cancer cells, whereby coordinated protein synthesis and folding within the tumor microenvironment maintain malignant phenotypes.^[^ ^4^, ^5^ ^]^ Chaperone proteins, as primary regulators of this process, facilitate proper protein folding and maintain cellular protein functionality.^[^ ^6^, ^7^ ^]^ Among these, the eukaryotic chaperonin‐containing TCP1 (CCT) complex, also known as the TCP1 ring complex (TRiC), plays a crucial role.^[^ ^7^, ^8^, ^9^ ^]^ The TRiC/CCT complex has a large molecular structure (≈1 MDa) comprising two stacked octameric rings formed by eight paralogous subunits (CCT1–8).^[^ ^10^, ^11^ ^]^ Its double‐ring structure creates distinct folding chambers: the equatorial domains provide adenosine triphosphatase activity, whereas the apical domains mediate substrate binding.^[^ ^10^, ^11^, ^12^ ^]^ The TRiC/CCT complex folds ≈10% of the cellular proteome,^[^ ^13^, ^14^ ^]^ including critical cytoskeletal proteins (actin and tubulin),^[^ ^15^, ^16^ ^]^ cell cycle‐associated proteins (CDH1, p27, CDC20, and PLK1),^[^ ^17^, ^18^, ^19^ ^]^ tumor suppressors (VHL and p53),^[^ ^20^, ^21^ ^]^ and oncogenic signaling proteins (STAT3, Raptor, and mLST8).^[^ ^22^, ^23^ ^]^ Despite its fundamental importance, the mechanistic regulation of TRiC/CCT assembly and function is poorly understood. Elevated expression of TRiC/CCT subunits in multiple cancers correlates with tumor progression, while depletion suppresses tumor growth, metastasis, and chemoresistance,^[^ ^24^, ^25^, ^26^ ^]^ highlighting the need to systematically investigate the TRiC/CCT oncogenic mechanisms to reveal novel therapeutic vulnerabilities.

Protein ubiquitination is an essential post‐translational modification mediated by a three‐enzyme cascade consisting of ubiquitin‐activating(E1), conjugating (E2), and ligase (E3) enzymes, which regulates cellular homeostasis through targeted protein degradation and functional remodeling.^[^ ^27^ ^]^ Among E3 ubiquitin ligases, which are key specificity determinants, the cullin‐RING ligase (CRL) family constitutes the largest group in humans.^[^ ^27^, ^28^ ^]^ An example is the CUL4‐RING ligase (CRL4) complex, which develops around the scaffold proteins CUL4A and CUL4B, integrating the catalytic RING protein RBX1 and utilizing the adaptor Damage‐specific DNA binding protein 1 (DDB1) to form substrate receptor modules.^[^ ^29^, ^30^ ^]^ Substrate recognition primarily occurs through DDB1‐CUL4‐associated factors (DCAFs), typically WD40‐repeat proteins containing a conserved WDxR motif for DDB1 binding.^[^ ^29^, ^30^, ^31^ ^]^ Notably, although the human genome encodes ≈90 DCAF proteins, ≈20 specific substrates have been confirmed,^[^ ^29^, ^32^, ^33^ ^]^ underscoring the need for systematic characterization of this biologically important protein family.

Dysregulation of the CRL4 complex, which is frequently manifested as upregulated expression of core components (CUL4A, CUL4B, RBX1, and DDB1), is associated with multiple cancer types.^[^ ^32^, ^33^ ^]^ Specific DCAF proteins, including DCAF1, DCAF2, DCAF4, DCAF5, DCAF6, DCAF7, DCAF13, and WDR4, promote tumorigenesis by degrading tumor suppressors, whereas others (AMBRA1, CRBN, COP1, AhR, and WDR70) exhibit tumor‐suppressive properties by degrading oncogenic proteins.^[^ ^32^, ^33^, ^34^, ^35^, ^36^, ^37^, ^38^, ^39^ ^]^ Although numerous DCAF proteins have been identified, their roles as oncogenes or tumor suppressors are largely unknown, presenting a critical knowledge gap in the development of targeted cancer therapies.

DCAF12, also known as WDR40A or TCC52, is an evolutionarily conserved regulator of cellular homeostasis with distinct functional roles in various biological contexts. Previous *Drosophila* genetic studies revealed that in developmental apoptosis, DCAF12 facilitates ubiquitination‐independent cleavage of the caspase inhibitor Diap1 to mediate Reaper/Hid/Grim‐dependent programmed cell death.^[^ ^40^ ^]^ This apoptotic function is crucial for morphogenetic tissue remodeling because DCAF12 deficiency leads to the accumulation of excess cells and works synergistically with the loss of tumor suppressors to promote neoplastic growth.^[^ ^40^ ^]^ Previous related studies have revealed the capacity of DCAF12 to regulate neuromuscular synaptic function in a ubiquitination‐dependent manner,^[^ ^41^ ^]^ demonstrating its mechanistic flexibility. In mammalian systems, DCAF12 regulates T cell homeostasis through ubiquitination of Moloney leukemia virus 10 homolog (MOV10) ^[^ ^42^ ^]^ and controls autophagic flux via degradation of melanoma antigen gene (MAGE) family proteins.^[^ ^43^ ^]^ Although these studies have elucidated the diverse physiological roles, the oncogenic potential of the canonical function of DCAF12 as a CRL4‐associated E3 ubiquitin ligase is inadequately characterized.

In this study, we demonstrate that DCAF12 is a significant oncogenic driver in NSCLC by promoting metastasis through non‐degradative ubiquitination of the TRiC/CCT complex. This post‐translational modification stabilizes the TRiC/CCT assembly and enhances its chaperone activity, thereby facilitating metastasis via two coordinated mechanisms: 1) cytoskeletal reorganization and 2) simultaneous activation of YAP, STAT3, and mTOR pathways. Our study uncovered a striking, context‐dependent functional switch for DCAF12, which exhibits oncogenic activity in mammals despite its tumor‐suppressive role in *Drosophila*, highlighting the therapeutic potential of targeting the DCAF12‐TRiC/CCT axis. These results expand the functional repertoire of DCAF proteins beyond their canonical role in protein degradation and establish chaperone ubiquitination as a novel regulatory mechanism in cancer progression.

## 2 Results

### 2.1 DCAF12 Drives Lung Adenocarcinoma Metastatic Progression

Our integrated bioinformatics and functional approach systematically identified DCAF family members involved in lung cancer pathogenesis. Analysis of The Cancer Genome Atlas (TCGA) data revealed that seven DCAF genes (*DCAF2, DCAF4, DCAF7, DCAF12, DCAF13, DCAF15*, and *DCAF17*) were significantly upregulated in lung adenocarcinoma (LUAD) (Figure S1A–C, Supporting Information). Functional screening using highly metastatic 889DTC cells, which harbor dTomato for tracing, demonstrated that knockdown of the corresponding mouse orthologs (except for *Dcaf4*) impaired cell migration (Figure S1D,E, Supporting Information), suggesting their role in motility regulation. Among these, *Dcaf12* was a key regulator, with siRNA knockdown inducing pronounced migration defects (Figure S1D,E, Supporting Information), while marginally affecting cell viability (Figure S1F, Supporting Information).

**Figure 1:**
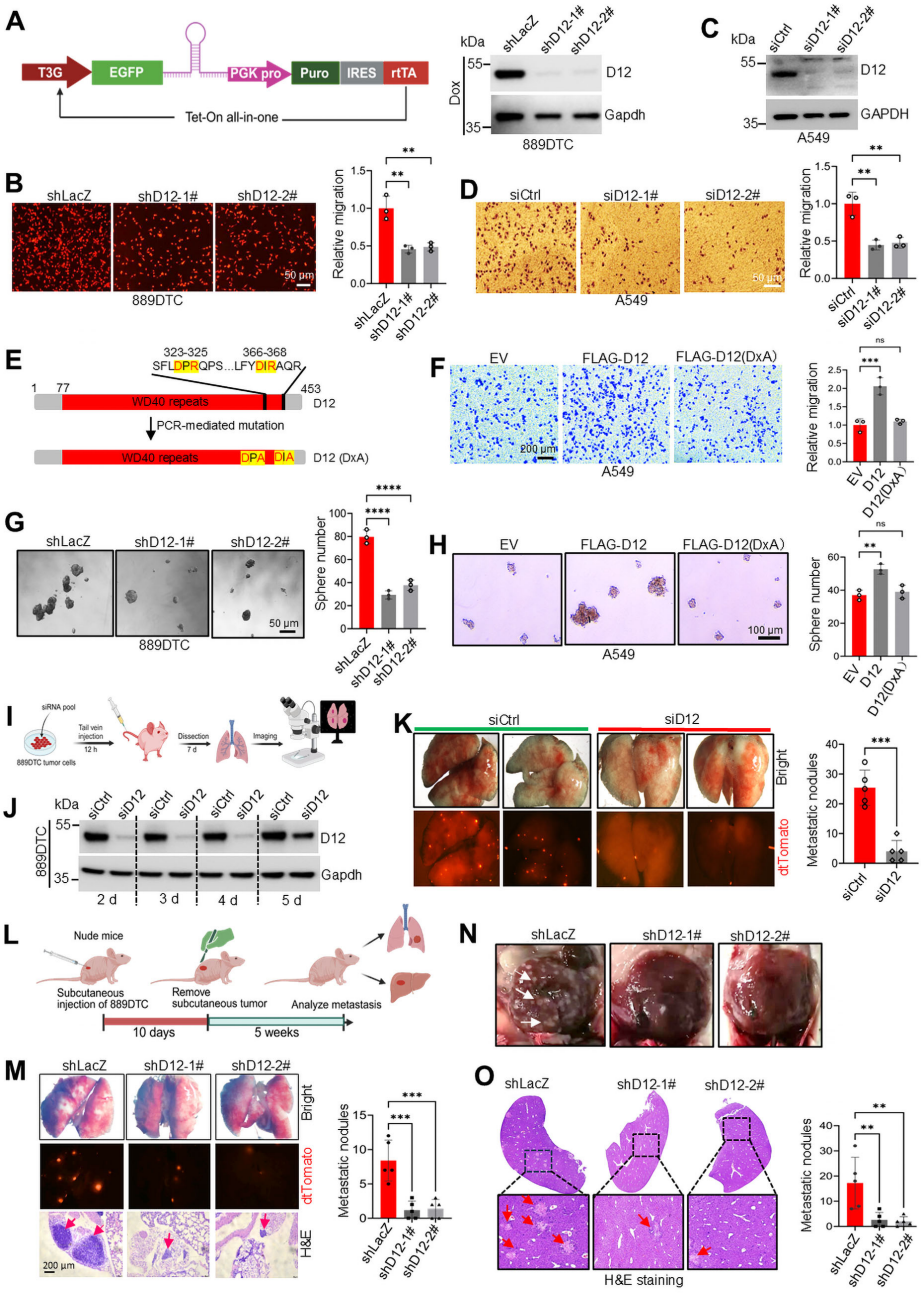
DCAF12 promotes metastatic progression in lung adenocarcinoma. A) Doxycycline‐inducible shRNA‐mediated knockdown of *Dcaf12* in dTomato‐labeled 889DTC cells. Knockdown efficiency was confirmed using an immunoblotting assay. B) Transwell migration assays revealed that Dcaf12 depletion significantly impaired the migratory capacity of 889DTC cells compared to that of the controls. Representative images (left) and quantitative analysis (right) based on dTomato fluorescence show the relative migration rates (*n* = 3 independent experiments). C) Immunoblot validation of the siRNA‐mediated *DCAF12* knockdown efficiency in A549 cells. D) *DCAF12* knockdown significantly reduced A549 cell migration in Transwell assays. Representative images (left) and quantitative analysis (right), as assessed using the ImageJ software, are shown (*n* = 3 independent experiments). E) Schematic representation of the PCR‐based site‐directed mutagenesis strategy used to generate DCAF12 DxR‐to‐DxA mutant. F) A549 cells overexpressing wild‐type DCAF12 exhibit enhanced migratory capacity. Representative images (left) and quantitative analysis (right) measured using ImageJ software are shown (*n* = 3 independent experiments). G)  Tumor sphere formation assays revealed impaired self‐renewal capacity in *Dcaf12*‐knockdown 889DTC cells, as evidenced by reduced sphere formation. Representative images (left) and quantitative analysis of sphere numbers (right) are shown (*n* = 3 independent experiments). H) Overexpression of wild‐type DCAF12, but not the DxA mutant, significantly enhanced tumor sphere formation in A549 cells. Representative images (left) and quantitative analysis of sphere numbers (right) are shown (*n* = 3 independent experiments). I) Schematic of the experimental metastasis assay: tail vein injection of control or *Dcaf12*‐knockdown 889DTC cells to assess the lung colonization potential. J) Western blot analysis confirmed efficient *Dcaf12* knockdown in siRNA‐transfected 889DTC cells and not in non‐targeting siRNA controls. K) Fluorescence microscopy quantification of dTomato‐positive foci revealed significantly reduced lung metastatic burden in mice injected with *Dcaf12*‐knockdown 889DTC cells 7 days post‐injection (*n* = 5 mice per group). L) Spontaneous metastasis model workflow: subcutaneous implantation of 889DTC cells, primary tumor resection at day 10, and metastasis assessment in lung/liver at 5 weeks (fluorescence microscopy and histology). M) *Dcaf12* knockdown significantly reduced lung metastasis in 889DTC‐injected mice 5 weeks of doxycycline treatment (representative H&E sections exhibiting metastatic nodules, arrows; *n* = 5 mice per group). N) Macroscopic examination revealed metastatic liver lesions in 889DTC‐injected mice. O) Histological analysis of liver metastases using H&E staining (representative metastatic nodules indicated by arrows) with quantitative assessment of nodules per section (*n* = 5 mice per group). Data are presented as mean ± SD. Significance is denoted by ^**^ *p* < 0.01, ^***^ *p* < 0.001, and ^****^ *p* < 0.0001. ‘ns’ indicates no significance. D12: DCAF12, D12 (DxA): inactive DCAF12, EV: empty control, H&E: hematoxylin and eosin.

To validate these findings, we used a tetracycline‐inducible shRNA system to achieve stable *Dcaf12* knockdown in 889DTC cells (**Figure** **1**A). This inducible suppression replicated the impaired migration observed with transient siRNA silencing Figure 1B, as confirmed by the wound healing assays (Figure S1G, Supporting Information). This conserved function was further demonstrated in human lung cancer lines (A549 and H1299), where *DCAF12* silencing significantly reduced motility in Transwell (Figure 1D; Figure S2A–C, Supporting Information) and wound healing assays (Figure S2D,E, Supporting Information). Through complementary gain‐of‐function approaches, we discovered that the activation of endogenous *DCAF12* in A549 cells via CRISPRa enhances migration (Figure S3A–C, Supporting Information). Similarly, overexpression of *DCAF12* promoted the migration of A549 and H460 cells. However, this effect was absent in the inactive DCAF12 (DxA) mutant, which was deficient in CRL4 binding (Figure 1E,F; Figure S3E,G, Supporting Information), demonstrating the CRL4‐dependent activity of DCAF12. Notably, while *DCAF12* manipulation strongly affected metastatic behavior, its knockdown had a minimal impact on clonogenic survival (Figures S1H and S2F, Supporting Information), indicating a role specific to metastasis rather than proliferation.

Considering the established connection between cancer stemness and metastasis,^[^ ^44^ ^]^ we investigated the role of DCAF12 in tumorsphere formation. Silencing *Dcaf12* (or its human homolog) reduced the size and number of spheres in 889DTC cells (Figure 1G), and H1299 cells (Figure S4A,B, Supporting Information). Contrastingly, CRISPRa‐mediated activation of endogenous *DCAF12* in A549 cells significantly enhanced tumorsphere formation (Figure S3D, Supporting Information).Similarly, DCAF12 overexpression increased sphere formation in A549 cells, whereas the DxA mutant had no significant effect (Figure 1H), suggesting that CRL4‐mediated ubiquitination was the underlying mechanism, consistent with the findings from the in vivo validation using two metastatic models. In experimental metastasis assays, mice injected with *Dcaf12*‐knockdown 889DTC cells developed significantly fewer lung nodules than those of the controls by day 7 (Figure 1I–K). Winslow spontaneous metastasis model,^[^ ^45^ ^]^ which recapitulates the full metastatic cascade, reduced metastasis in both lungs (Figure 1M) and the liver (Figure 1N) 5 weeks after primary tumor resection (Figure 1L). Combined, these results established that DCAF12 drives lung adenocarcinoma metastasis mediated by CRL4‐mediated ubiquitination.

### 2.2 DCAF12 Directly Interacts with the TRiC/CCT Chaperonin Complex

**Figure 2:**
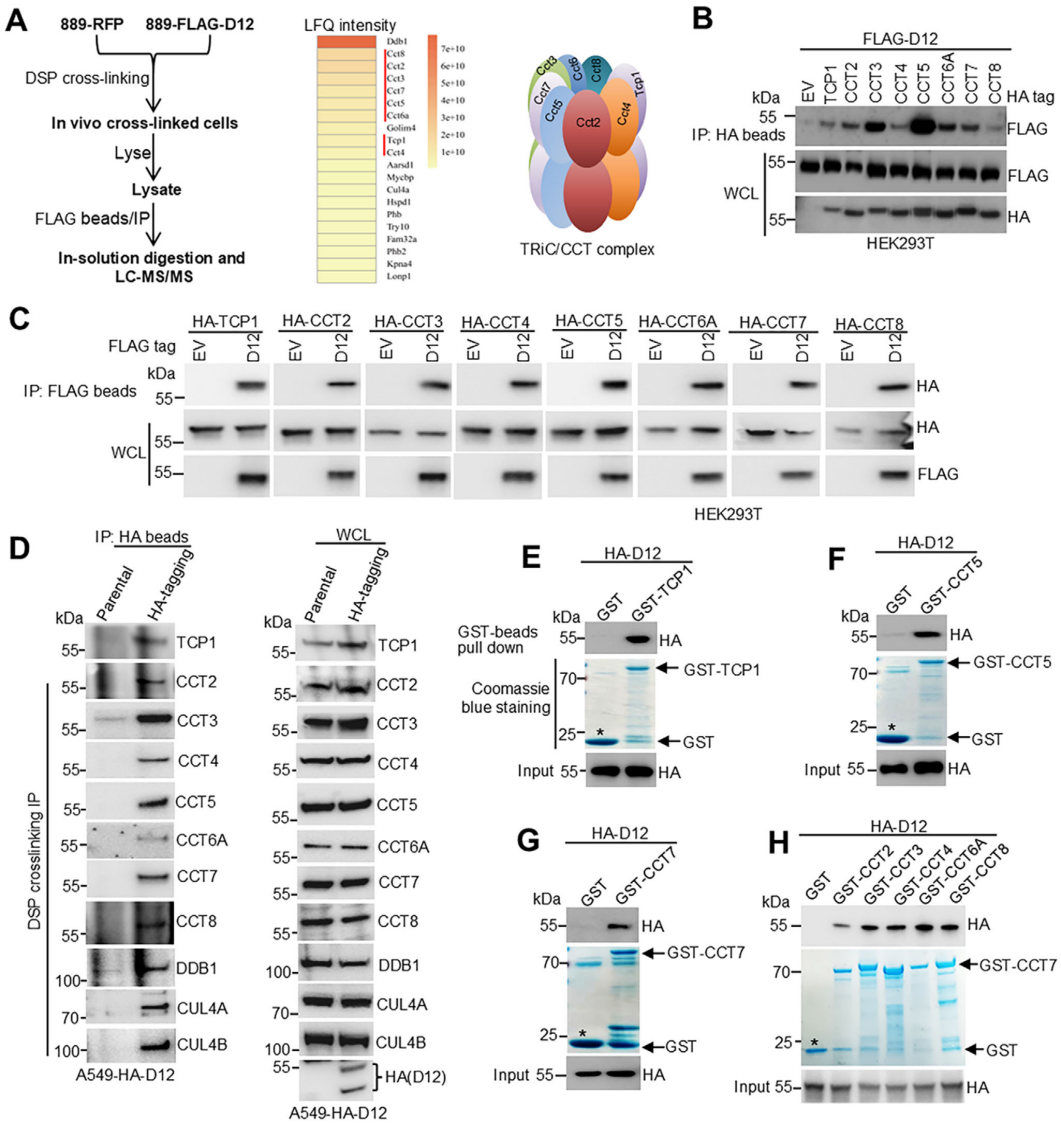
DCAF12 interacts with the TRiC/CCT subunits. A) Proteomic analysis of Dcaf12 interactors. Schematic representation of the mass spectrometry workflow used to analyze Dcaf12‐associated proteins (left). CRL4 and TRiC/CCT complex subunits were identified as the major Dcaf12‐binding partners (middle and right). B) Co‐IP of FLAG‐DCAF12 with the HA‐tagged TRiC/CCT subunits. HA‐tagged TRiC/CCT subunits were immunoprecipitated with anti‐HA beads, and bound FLAG‐DCAF12 was detected using anti‐FLAG immunoblotting. C) Reciprocal co‐IP validation of the DCAF12‐TRiC/CCT interaction. FLAG‐DCAF12 was immunoprecipitated with anti‐FLAG beads, and the associated HA‐tagged TRiC/CCT subunits were detected using an anti‐HA immunoblotting assay. D) Protein–protein interaction analysis of endogenously HA‐tagged DCAF12 in A549 cells. HA‐DCAF12 was immunoprecipitated using anti‐HA antibodies, followed by immunoblotting detection of the associated endogenous TRiC/CCT subunits and CRL4 complex subunits with specific antibodies. E–H) GST pull‐down assays confirmed direct binding between DCAF12 and TRiC/CCT subunits. Recombinant GST‐tagged CCT subunits (expressed in *Escherichia coli*) were incubated with HA‐DCAF12 (from HEK293T lysates). GST‐bound complexes were analyzed using anti‐HA immunoblotting to detect HA‐DCAF12 association. The asterisk denotes purified GST protein.

To elucidate the molecular basis of DCAF12‐mediated metastasis, we performed a proteomic screen to identify the potential ubiquitination substrates. In 889DTC cells stably expressing FLAG‐tagged Dcaf12, we employed crosslinking with dithiobis(succinimidyl propionate) (DSP) followed by immunoprecipitation (IP) to capture transient interactions (Figure 2A). Mass spectrometry analysis revealed two key insights. First, we detected strong enrichment of core CRL4 components (Ddb1 and Cul4A) (Figure 2A, middle panel), confirming the involvement of Dcaf12 in ubiquitin ligase complexes, consistent with our IP results in human cell lines (Figure S3F,G, Supporting Information). Second, we observed robust co‐enrichment of all eight TRiC/CCT subunits in the Dcaf12 pull‐downs (Figure 2A, middle and right panels), revealing an association between ubiquitination pathways and protein folding machinery.

Given their established oncogenic roles ^[^ ^25^, ^26^ ^]^ and their overexpression in lung cancer (Figure S5A–D, Supporting Information), we systematically validated these interactions. Co‐IP of 889DTC‐FLAG‐Dcaf12 cells confirmed the association of Dcaf12 with the endogenous TRiC/CCT subunits (Figure S5E, Supporting Information). To assess the binding specificity, we co‐expressed HA‐tagged human TRiC/CCT subunits with FLAG‐DCAF12 in HEK293T cells. HA‐IP assays revealed interactions between DCAF12 and all TRiC/CCT subunits, with particularly strong binding to CCT3 and CCT5, as validated by reciprocal co‐IP (Figure  2C). Notably, these interactions were highly specific, as control DCAF proteins (DCAF2, DCAF7, and DCAF13) with conserved WD40 domains ^[^ ^31^ ^]^ exhibited negligible (DCAF2 and DCAF7) or weak (DCAF13) binding to the TRiC/CCT subunits (Figure S6A,B, Supporting Information).

To evaluate the physiological relevance, we generated A549 cells with endogenously HA‐tagged DCAF12 using CRISPR/Cas9 (Figure S6D,E, Supporting Information). HA pull‐downs in these cells recapitulated the interactions with all the TRiC/CCT subunits (Figure 2D). GST pull‐down assays with bacterially expressed TRiC/CCT subunits and eukaryotic HA‐DCAF12 confirmed the direct physical binding (Figure 2E–H), excluding artifacts from mammalian post‐translational modifications. Using this multifaceted approach, we established that DCAF12 is a physiological interactor of the TRiC/CCT complex, implicating its potential role in modulating TRiC/CCT function during lung cancer progression.

### 2.3 DCAF12 Catalyzes Non‐Degradative Ubiquitination of TRiC/CCT Subunits

**Figure 3:**
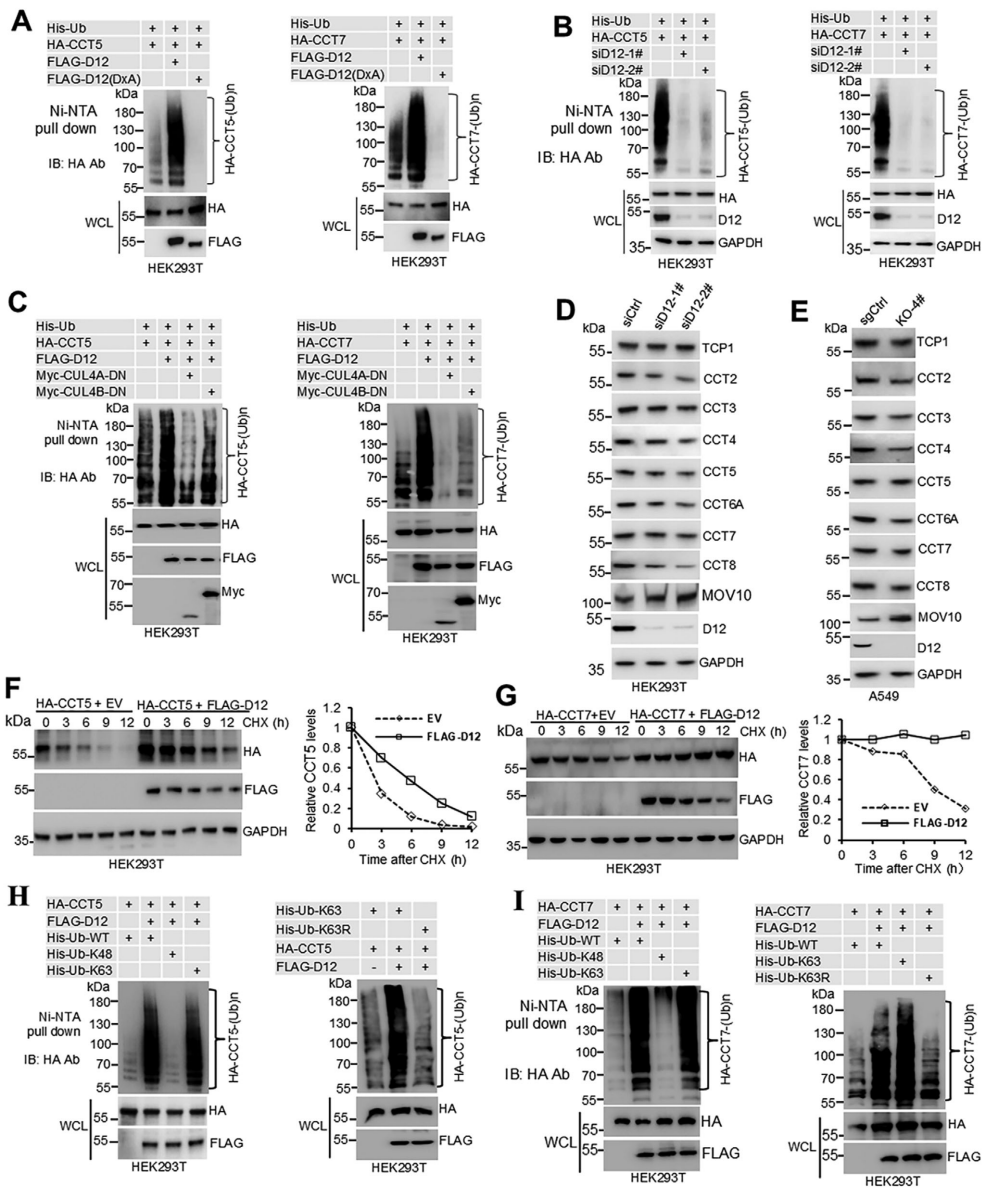
DCAF12 promotes non‐degradative ubiquitination of TRiC/CCT subunits. A) In vivo ubiquitination assay of HA‐tagged TRiC/CCT subunits in HEK293T cells. The cells were co‐transfected with HA‐TRiC/CCT subunit constructs, His‐tagged ubiquitin (His‐Ub), and wild‐type or mutant (DxA) FLAG‐DCAF12. Ubiquitinated proteins were purified under denaturing conditions using Ni‐NTA affinity chromatography and were analyzed by immunoblotting with an anti‐HA antibody. B) Effect of *DCAF12* knockdown (siRNA) on TRiC/CCT subunit ubiquitination. C) Role of dominant‐negative CUL4A/B (CUL4A‐DN, CUL4B‐DN) in DCAF12‐mediated TRiC/CCT ubiquitination. D) TRiC/CCT subunit expression after *DCAF12* siRNA knockdown in HEK293T cells. E) TRiC/CCT subunit levels in *DCAF12*‐knockout A549 cells. F,G) Protein degradation kinetics of HA‐tagged CCT5 (F) and CCT7 (G) in the presence of FLAG‐DCAF12. HEK293T cells were treated with CHX to inhibit protein synthesis, and the stability of CCT5 and CCT7 was assessed using immunoblotting. Protein half‐lives were determined using densitometric analysis of band intensities over time. H,I) DCAF12‐dependent K63‐linked ubiquitination of CCT5 (H) and CCT7 (I). HEK293T cells were co‐transfected with HA‐tagged CCT5 or CCT7, FLAG‐DCAF12, and either His‐tagged wild‐type ubiquitin (His‐Ub), K63‐only (His‐Ub‐K63), K48‐only (His‐Ub‐K48), or K63R ubiquitin mutants. Ubiquitinated proteins were isolated by Ni‐NTA pull‐down and analyzed using anti‐HA immunoblotting. Ctrl: control, D12: DCAF12, DN: dominant negative, KO: knockout, CHX: cycloheximide.

Having established the physical interaction between DCAF12 and TRiC/CCT subunits, next, we investigated its functional role as a CRL4 substrate receptor in mediating TRiC/CCT subunit ubiquitination. Histidine‐tagged ubiquitination assays demonstrated that co‐expression of FLAG‐DCAF12 with HA‐tagged TRiC/CCT subunits resulted in marked enhancement in ubiquitination levels. The catalytically inactive DxA mutant failed to promote ubiquitination and exerted a dominant‐negative effect, significantly suppressing basal ubiquitination levels (**Figure** **3**A; Figure S7A–F, Supporting Information). This regulatory mechanism was further validated through siRNA‐mediated knockdown of endogenous DCAF12, which substantially reduced the ubiquitination of exogenously expressed TCP1, CCT2, CCT5, and CCT7 (Figure 3B; Figure S8A,B, Supporting information). The DCAF12‐dependent ubiquitination of TCP1, CCT2, CCT3, CCT5, and CCT7 was eradicated by the co‐expression of dominant‐negative CUL4A/B mutants with no E3 ligase activity (Figure 3C; Figure S9A–C, Supporting Information). Together, the results revealed that CRL4‐DCAF12 is the specific E3 ligase responsible for TRiC/CCT subunit ubiquitination.

To assess the functional effect of DCAF12‐mediated ubiquitination, we examined the stability of the TRiC/CCT subunit. While FLAG‐DCAF12 overexpression in A549 cells downregulated the known CRL4‐DCAF12 substrates (MOV10 and MAGEA3), it did not reduce endogenous TRiC/CCT levels (Figure S10A, Supporting Information). The DxA mutant had marginal effects on all tested proteins (Figure S10A, Supporting Information). Conversely, DCAF12 silencing in HEK293T cells increased MOV10 abundance, but paradoxically destabilized most TRiC/CCT subunits, except TCP1 and CCT5 (Figure 3D). This destabilization was recapitulated in DCAF12‐depleted A549 cells (Figure 3E). Pharmacological inhibition of CRLs with MLN4924 (pevonedistat) elevated NRF2 levels, but did not increase TRiC/CCT subunit levels (Figure S10B, Supporting Information), thereby ruling out a CRL‐mediated degradation mechanism. This is consistent with the Cycloheximide (CHX) chase assays showing that DCAF12 overexpression extended the half‐life of TCP1, CCT5, and CCT7 (Figure 3F,G; Figure S11, Supporting Information), supporting a non‐degradative mode of regulation.

To characterize ubiquitin linkage specificity, we first examined K63‐linked modifications, considering their established roles in non‐degradative signaling.^[^ ^46^, ^47^ ^]^ Ubiquitination assays revealed that DCAF12 predominantly mediates K63‐linked polyubiquitination of TCP1, CCT3, CCT5, and CCT7 subunits, with minimal K48‐linked modifications. This K63‐linkage dependence was confirmed by the elimination of ubiquitination in Ub‐K63R‐expressing cells (Figure 3H,I; Figure S12A–C, Supporting Information). Using a comprehensive ubiquitin mutant panel (K0, K6, K11, K27, K29, K33, K48, and K63), we further identified selective K27‐linked ubiquitination of CCT2, CCT3, CCT4, CCT6A, and CCT8, proven by the absence of ubiquitination signals in Ub‐K27R cells (Figure S12D–K, Supporting Information), revealing the ability of DCAF12 to establish subunit‐specific ubiquitination codes through distinct K63 and K27 linkages, both associated with non‐degradative functions.^[^ ^46^, ^47^, ^48^ ^]^

### 2.4 TRiC/CCT Ubiquitination is Essential for the Pro‐Metastatic Function of DCAF12

**Figure 4:**
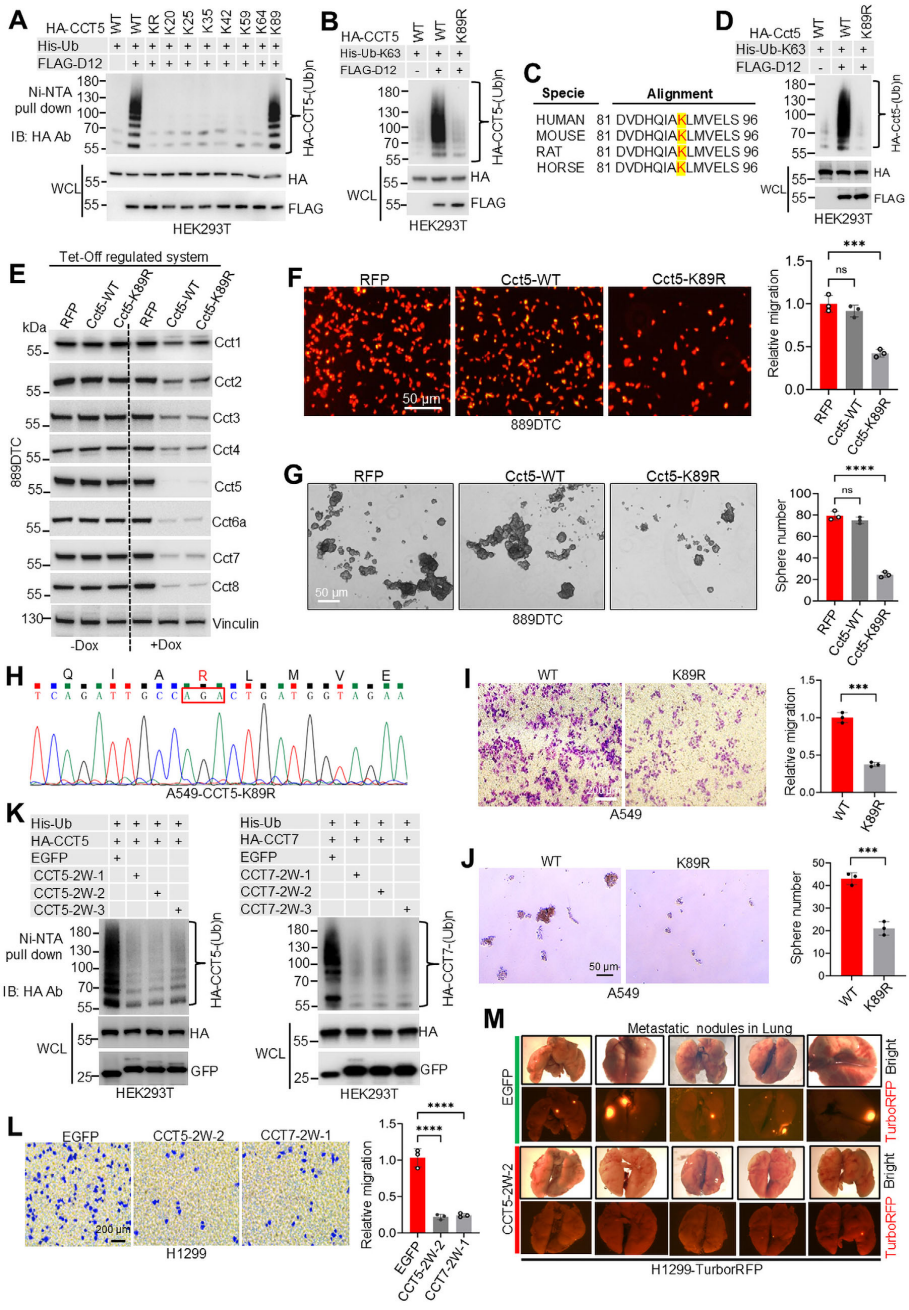
DCAF12‐mediated ubiquitination of TRiC/CCT subunits promotes lung cancer progression. A) Mapping of the DCAF12‐dependent ubiquitination sites on CCT5. Ubiquitination assays in HEK293T cells expressing HA‐tagged wild‐type (WT) CCT5, lysine‐to‐arginine (KR) mutants, or single lysine mutants identified specific modification sites. B) DCAF12 specifically targets ubiquitination of CCT5 K89. HEK293T cells co‐expressing HA‐tagged WT or K89R CCT5 with FLAG‐DCAF12 and His‐ubiquitin were subjected to ubiquitination pull‐down assay. C) Conservation of species by DCAF12‐mediated ubiquitination. Ubiquitination of mouse Cct5 at K89 was assessed in HEK293T cells expressing HA‐tagged WT, K89, or K89R mouse Cct5 with FLAG‐Dcaf12 and His‐ubiquitin. D) Species conservation of DCAF12‐mediated ubiquitination. Mouse Cct5 ubiquitination at K89 was assessed in HEK293T cells expressing HA‐tagged WT, K89, or K89R mouse Cct5 with FLAG‐Dcaf12 and His‐ubiquitin. E) Functional reconstitution of the TRiC/CCT complex in 889DTC cells. CRISPR/Cas9‐mediated *Cct5* knockout cells were engineered with the Tet‐Off‐regulated expression of the WT mouse or K89R Cct5. Western blotting confirmed complete ablation of endogenous Cct5 and stringent doxycycline control of transgene expression. F,G) Phenotypic consequences of the Cct5 K89R mutation in 889DTC cells. Transwell migration and 3D tumor spheroid formation assays revealed significantly impaired cell motility (F) and reduced self‐renewal capacity (G), respectively (mean SD from three independent experiments H) Genotypic validation of the CCT5 K89R knock‐in in A549 cells using Sanger sequencing (representative chromatogram is shown). I,J) CCT5 K89R knock‐in A549 cells exhibited reduced migration (I) and tumor spheroid formation (J) compared to WT (mean ± SD from three independent experiments K) Peptide screening identified CCT5‐2W‐2 and CCT7‐2W‐1 as inhibitors of CCT5 (left) and CCT7 (right) ubiquitination, respectively. L) Ubiquitination inhibitory peptides (CCT5‐2W‐2 and CCT7‐2W‐1) suppressed H1299 cell migration. M) Pharmacological inhibition of CCT5 ubiquitination suppresses metastasis. H1299 cells expressing CCT5‐2W‐2 or EGFP (control) were injected into the tail vein (*n* = 5 mice per group). Lung metastatic burden was quantified 6 weeks post‐injection using ex vivo fluorescence imaging (representative images shown). Significance levels are indicated as follows: ^***^ *p* < 0.001, ^****^ *p* < 0.0001; ns, not significant.

To elucidate the functional significance of DCAF12‐mediated TRiC/CCT ubiquitination, we systematically mapped the critical modification sites. Considering the strong binding preference of DCAF12 for CCT5, we engineered a lysine (K)‐free CCT5 mutant (CCT5‐KR) by substituting all lysine residues with arginine (R). Domain mapping using a series of overlapping CCT5‐KR mutants (KR1‐KR5; Figure S13A, Supporting Information) revealed that the KR1 region (residues 25–89) is sufficient for ubiquitination (Figure S13B, Supporting Information), suggesting that it contains critical modification sites. The reintroduction of lysine 89 (K89) into the CCT5‐KR background (CCT5‐K89) restored ubiquitination (**Figure** **4**A), whereas the K89R mutation in wild‐type CCT5 significantly reduced this modification (Figure 4B). The essential role of K89 was further corroborated by its evolutionary conservation across species (Figure 4C) and reduced ubiquitination of mouse Cct5‐K89R relative to the wild‐type protein (Figure 4D).

To assess the functional consequences of K89 ubiquitination, we developed a dual CRISPR‐Cas9/Tet‐Off system in 889DTC cells. This system employed synonymous codon mutagenesis to generate tetracycline‐regulatable sgRNA‐resistant vectors expressing either wild‐type Cct5 or the K89R mutant. Following CRISPR‐mediated knockout of endogenous Cct5, we reconstituted the TRiC/CCT complex with these variants (Figure S14, Supporting Information), thereby circumventing the lethality associated with complete Cct5 loss. As previously reported,^[^ ^49^, ^50^ ^]^ doxycycline‐induced depletion of Cct5 led to universal destabilization of the TRiC/CCT complex (Figure 4E), confirming the structural dependence of the complex. Reconstitution with our system preserved the native expression levels and subunit stoichiometry (Figure 4E). Functionally, Cct5‐K89R cells recapitulated the phenotype observed after *Dcaf12* knockdown, demonstrating significantly impaired migration and tumorsphere formation capacity, with minimal impact on cell viability (Figure 4F,G; Figure S14B, Supporting Information). These findings were consistent with results from A549 cells harboring homozygous CCT5 K89R knock‐in mutations (Figure 4H; Figure S15A,B, Supporting Information), which likewise exhibited suppressed motility and tumorsphere formation without significant cytotoxicity (Figure 4I,J; Figure S15C, Supporting Information). Collectively, these results demonstrated that the ubiquitination of CCT5 at K89 is crucial for the pro‐metastatic function of DCAF12.

To translate these mechanistic insights into therapeutic strategies, we designed inhibitory peptides targeting the DCAF12‐CCT3/5/7 interaction interface (Figures S16A–C and S17A–D, Supporting Information). Three lead peptides, CCT3‐2W, CCT5‐2W‐2, and CCT7‐2W‐1, inhibited DCAF12‐mediated ubiquitination of the corresponding subunits (Figure 4K; Figures S16D and S17E,F, Supporting Information). Upon induction via the Tet‐On expression system in A549 and H1299 cells, these peptides demonstrated potent anti‐metastatic effects by significantly inhibiting cell migration (Figure 4L; Figure S17G, Supporting Information) and tumorsphere formation (Figure S17H,I, Supporting Information) with negligible cytotoxicity (Figure S17J, Supporting Information). These effects reflect the phenotypes observed with DCAF12 depletion or CCT5‐K89R mutation, as previously described. In vivo, H1299 cells expressing CCT5‐2W‐2 produced significantly fewer lung metastatic nodules than controls (Figure 4M), thereby replicating the anti‐metastatic effects observed with *Dcaf12* knockdown in 889DTC cells (Figure 1K,M–O). Together, these results confirm that DCAF12 promotes lung cancer metastasis through ubiquitination‐dependent regulation of TRiC/CCT subunits.

### 2.5 DCAF12‐Mediated Ubiquitination Maintains TRiC/CCT Structural and Functional Integrity

**Figure 5:**
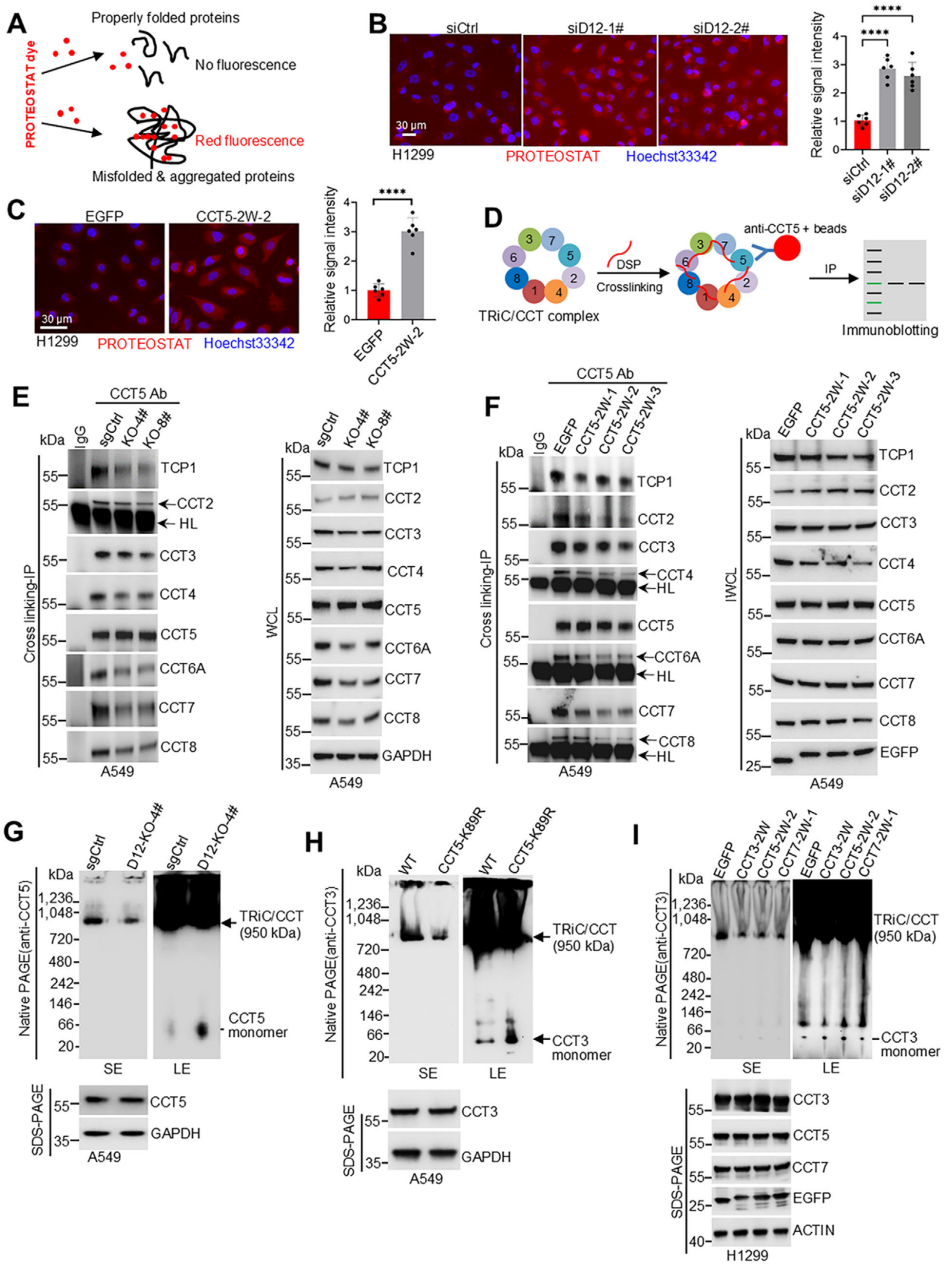
DCAF12 maintains TRiC/CCT complex integrity through ubiquitin‐dependent regulation. A) Schematic representation of the PROTEOSTAT aggregation assay. The fluorescent dye binds to misfolded/aggregated proteins, enabling aggregate quantification using fluorescence microscopy. B) *DCAF12* knockdown induces protein aggregation in H1299 cells. Representative images show increased PROTEOSTAT fluorescence (red) in DCAF12‐depleted cells (Hoechst33342, blue). Quantification revealed a significant increase in aggregates (mean ± SD; six random fields per condition; ^****^ *p* < 0.0001). Data are representative of two independent experiments. C) Inhibition of CCT5 ubiquitination mimicked DCAF12 depletion. H1299 cells expressing the CCT5‐2W‐2 peptide exhibited a significant increase in PROTEOSTAT fluorescence (mean ± SD; six random fields per condition; ^****^ *p* < 0.0001). Data are representative of two independent experiments. D) Experimental workflow for the TRiC/CCT stability assessment. The cells were treated with a DSP crosslinker before CCT5 IP and subunit analysis. E) TRiC/CCT subunit co‐IP in DCAF12‐KO A549 cells. Anti‐CCT5 IP after DSP crosslinking exhibited reduced interaction with other TRiC subunits (whole‐cell lysate input controls shown). F) Inhibition of ubiquitination disrupts TRiC/CCT interactions. CCT5 IP in A549 cells treated with CCT5 ubiquitination inhibitors revealed impaired subunit binding (DSP‐crosslinked samples). G) Native PAGE (anti‐CCT5) showing compromised TRiC/CCT integrity in DCAF12‐KO A549 cells. The total CCT5 levels were confirmed by SDS‐PAGE (GAPDH loading control). H) Native PAGE (anti‐CCT3) revealed assembly defects in CCT5 K89R knock‐in A549 cells (SE: short exposure; LE: long exposure). The total CCT3 levels remained unchanged (SDS‐PAGE normalized to GAPDH). I) Ubiquitination‐targeting peptides disrupted TRiC/CCT assembly. Treatment with CCT3‐2W, CCT5‐2W‐2, or CCT7‐2W‐1 impaired complex formation in H1299 cells (native PAGE anti‐CCT3). Equal loading was confirmed by SDS‐PAGE (GAPDH).

The TRiC/CCT chaperonin complex maintains cellular proteostasis through its protein‐folding activity.^[^ ^7^, ^49^ ^]^ To explore the regulatory role of DCAF12‐mediated ubiquitination, we evaluated the effect on TRiC/CCT function by assessing protein aggregation. Using three complementary methods, *DCAF12* knockout in A549 cells, *DCAF12* knockdown in H1299 cells, and expression of the ubiquitination‐inhibiting CCT5‐2W‐2 peptide in H1299 cells, quantitative PROTEOSTAT analysis consistently demonstrated significant protein aggregation (**Figure** **5**A–C, Figure S18, Supporting Information), indicating that DCAF12‐dependent ubiquitination is vital for maintaining the protein‐folding capacity of TRiC/CCT.

We performed a comprehensive biochemical characterization to elucidate the underlying mechanisms. Cross‐linking IP assays using DSP‐treated lysates revealed that DCAF12 deletion substantially impaired the interaction between CCT5 and the other TRiC/CCT subunits (Figure 5D,E), consistent with the findings of experiments with CCT5‐targeted ubiquitination‐blocking peptides, which revealed disruption of complex integrity (Figure 5F), indicating that DCAF12‐mediated ubiquitination stabilizes critical intersubunit interactions. Although the total subunit expression was unaltered, *DCAF12*‐knockdown HEK293T cells exhibited a notable reduction in intact 1 MDa TRiC/CCT complexes (Figure S19A, Supporting Information). Similarly, *DCAF12*‐KO A549 cells exhibited reduced complex formation and increased monomeric CCT5 levels on extended immunoblotting (Figure 5G). The ubiquitination dependence of these structural defects was demonstrated using three independent approaches: i) analysis of CCT5 K89R knock‐in A549 cells (Figure 5H); ii) evaluation of H1299 cells with peptides inhibiting the ubiquitination of CCT3, CCT5, or CCT7 (all presenting similar assembly defects and elevated CCT3 monomer levels; Figure 5I); and iii) pharmacological inhibition of CRLs using MLN4924, which replicated the structural defects (Figure S19B, Supporting Information).The ubiquitination dependence of these structural defects was demonstrated using three independent approaches. i) Analysis of CCT5 K89R knock‐in A549 cells (Figure 5H); ii) evaluation of H1299 cells with peptides inhibiting the ubiquitination of CCT3, CCT5, or CCT7 (all presenting similar assembly defects and elevated CCT3 monomer levels; Figure 5I); and iii) pharmacological inhibition of CRLs using MLN4924, which replicated the structural defects (Figure S19B, Supporting Information). Collectively, these findings establish that DCAF12‐mediated ubiquitination is a post‐translational modification that maintains TRiC/CCT complex integrity, with important implications for chaperonin assembly and cellular proteostasis.

### 2.6 DCAF12‐TRiC/CCT Axis Integrates Cytoskeletal Remodeling with Oncogenic Signaling

**Figure 6:**
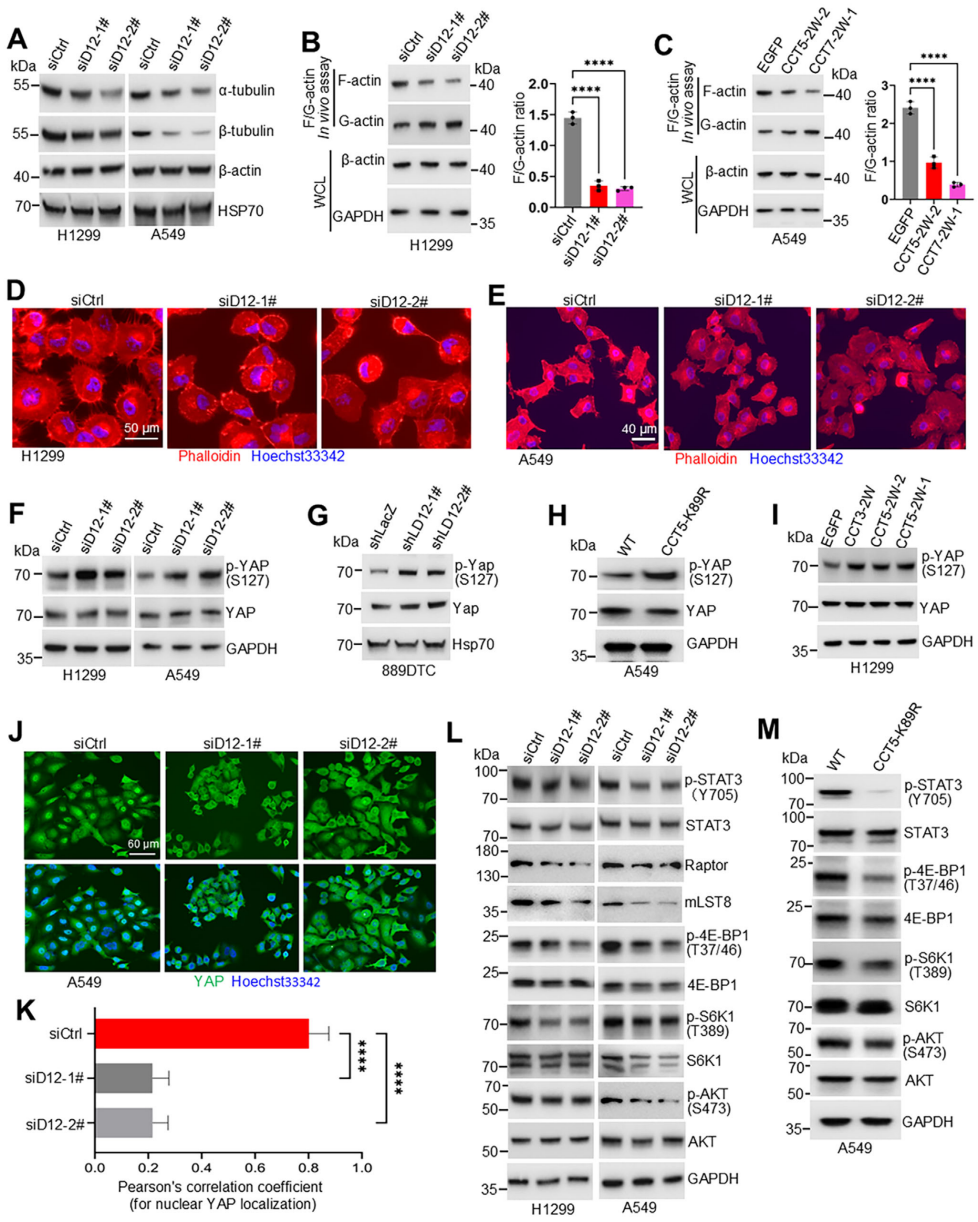
DCAF12 regulates cytoskeletal dynamics and oncogenic signaling pathways. A) Immunoblot analysis of tubulin and actin expression after *DCAF12* knockdown in H1299 and A549 cells. B) DCAF12 depletion alters actin polymerization. Quantification of F‐actin and G‐actin content (ImageJ analysis) revealed a significant decrease in the F‐actin/G‐actin ratio. C) Disruption of DCAF12‐CCT5/CCT7 interactions similarly decreased the F‐actin/G‐actin ratio. D,E) Phalloidin staining (Alexa Fluor 594) showed impaired filopodia formation in DCAF12‐depleted H1299 (D) and A549 (E) cells compared to that in controls. F,G) Immunoblots indicating increased YAP phosphorylation (S127) following *DCAF12* knockdown in H1299, A549 (F), and 889DTC (G) cells. H,I) Immunoblots indicating enhanced YAP phosphorylation in A549 cells with the CCT5 K89R mutation (H) and in H1299 cells treated with the CCT5‐2W‐2 peptide (I). J,K) Immunofluorescence staining showed reduced nuclear translocation of YAP upon *DCAF12* silencing (J). The nuclear colocalization of YAP with DAPI was quantified using Pearson's correlation coefficient (K). L,M) *DCAF12* knockdown (L) and the CCT5 K89R mutation (M) attenuated oncogenic signaling, as evidenced by reduced phosphorylation of STAT3, 4EBP‐1, S6K1, and AKT. Data represent the mean ± SD of three independent experiments, ^****^ *p* < 0.0001.

Our proteomic analysis, utilizing DSP crosslinking with CCT5 IP and LC‐MS/MS, identified key TRiC/CCT substrates, including the cytoskeletal regulators actin and tubulin (Figure S20A, Supporting Information), which are essential for cell motility.^[^ ^51^, ^52^ ^]^ *DCAF12 *knockdown in H1299 and A549 cells specifically reduced α‐ and β‐tubulin levels without altering β‐actin expression (**Figure** **6**A). This phenotype was recapitulated in *DCAF12*‐knockout A549 cells (Figure S20B, Supporting Information) and is consistent with the results observed in TRiC/CCT‐deficient cells.^[^ ^14^ ^]^ The overexpression of wild‐type DCAF12 increased tubulin levels, whereas the CRL4‐binding‐deficient DxA mutant lacked this capacity (Figure S20C, Supporting Information), thus establishing DCAF12 as a ubiquitination‐dependent regulator of microtubule homeostasis. A detailed analysis of actin organization revealed that DCAF12 depletion significantly accumulated G‐actin in H1299 cells (Figure 6B), indicating impaired F‐actin polymerization. This defect was replicated by treatment with ubiquitination‐blocking peptides that targeted either CCT5 or CCT7 (Figure 6C). Phalloidin staining confirmed the functional consequences, revealing markedly reduced filopodia formation (Figure 6D,E). The coordinated disruption of the microtubule and microfilament networks provides a mechanistic explanation for the observed migration defects.

Given the interplay between cytoskeletal tension and Hippo pathway activation,^[^ ^53^, ^54^ ^]^ we investigated the role of DCAF12 in YAP/TAZ regulation. *DCAF12* knockdown increased YAP phosphorylation (Ser127) in H1299, A549, and 889DTC cells (Figure 6F,G; Figure S19B, Supporting Information), indicating pathway inactivation. Conversely, overexpression of wild‐type DCAF12 decreased YAP phosphorylation, whereas overexpression of DxA mutant did not (Figure S20C, Supporting Information), indicating a ubiquitination‐dependent regulation. Orthogonal validation using 1) CCT5‐K89R knock‐in A549 cells and 2) inhibitory peptides disrupting DCAF12‐CCT interactions in H1299 cells confirmed this mechanism; both approaches increased YAP phosphorylation (Figure 6H,I) and reduced nuclear YAP localization (Figure 6J,K). Taken together, these results mechanistically link DCAF12‐mediated ubiquitination to cytoskeletal mechanotransduction.

In addition to cytoskeletal regulation, the DCAF12‐TRiC/CCT axis modulates oncogenic signaling pathways, including STAT3 and mTOR. This finding extends the established role of TRiC/CCT in folding and maturation of key oncoproteins such as STAT3, Raptor, and mLST8.^[^ ^22^, ^23^ ^]^ In DCAF12‐depleted H1299 and A549 cells, we observed reduced STAT3 phosphorylation (Y705) and impaired mTOR signaling, as evidenced by destabilized Raptor/mLST8 and decreased phosphorylation of 4EBP1 (T37/46), S6K1 (T389), and AKT (S473) (Figure 6L). The ubiquitination dependence of these effects was confirmed using the CCT5‐K89R mutants (Figure 6M) and CCT‐targeting inhibitory peptides (Figure S20D, Supporting Information), both of which phenocopied DCAF12 depletion. Consistently, CCT6A‐ or CCT8‐depleted cells exhibited analogous signaling defects (Figure S20E, Supporting Information), underscoring the essential role of TRiC/CCT in mediating the oncogenic functions of DCAF12.

### 2.7 Targeting the TRiC/CCT Complex Inhibits Lung Cancer Metastasis

**Figure 7:**
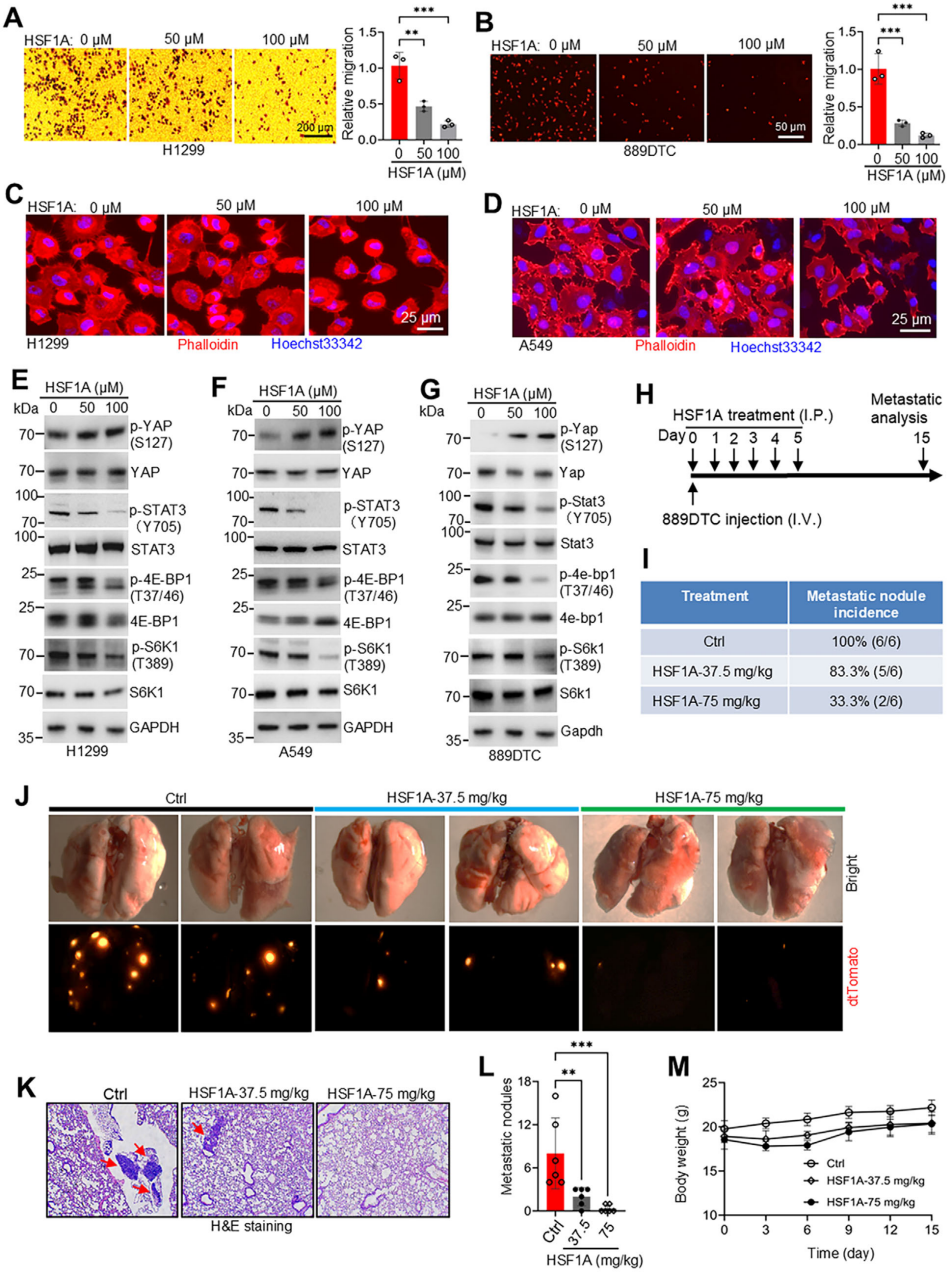
Pharmacological inhibition of the TRiC/CCT complex suppresses lung cancer metastasis. A,B) Transwell migration assays reveal that 18 h HSF1A treatment significantly inhibited the migratory capacity of H1299 (A) and 889DTC (B) lung cancer cells (mean ± SD; *n* = 3 independent experiments; ^**^ *p* < 0.01, and ^***^ *p* < 0.001). C,D) Phalloidin staining demonstrates impaired filopodia formation in A549 (C) and H1299 (D) cells after 18 h HSF1A treatment, indicating cytoskeletal disruption. E–G) Western blot analysis showing HSF1A‐mediated downregulation of key oncogenic pathways, including YAP, STAT3, and mTOR signaling, in H1299 (E), A549 (F), and 889DTC (G) cells following 18 h treatment with HSF1A. H) Experimental metastasis model: Nude mice (*n* = 6 per group) were intravenously injected with 1 × 10⁵ 889DTC cells and subsequently treated for 6 days with either vehicle control (5% DMSO in corn oil) or HSFA1 (37.5 or 75 mg kg^−1^) over a 15‐day observation period. I) Quantitative assessment of the metastatic burden by fluorescence imaging exhibited a dose‐dependent inhibition of lung colonization. J) Representative whole‐lung fluorescence images captured on day 15 post‐injection, demonstrating reduced metastatic foci in HSFA1‐treated groups. K,L) Histopathological analysis of H&E‐stained lung sections (scale bar = 100 µm), with arrows indicating metastatic nodules (K) and quantitative evaluation of lung metastatic foci (L) (mean ± SD; ^**^ *p* < 0.01, ^***^ *p* < 0.001). M) Monitoring of mouse body weight throughout the 15‐day treatment period confirmed treatment tolerability.

Considering the pivotal role of the DCAF12‐TRiC/CCT axis in cytoskeletal dynamics and the regulation of oncogenic pathways, such as YAP, STAT3, and mTOR, in lung cancer cells, we investigated the therapeutic potential of HSF1A, a novel small‐molecule inhibitor of the TRiC/CCT complex.^[^ ^55^ ^]^ After treating cells with HSF1A for 18 h, we observed dose‐dependent inhibition of migration in H1299 and 889DTC cells (**Figure** **7**A). Consistently, phalloidin staining revealed that HSF1A treatment significantly reduced filopodia formation in A549 and H1299 cells, indicating disruption of actin‐dependent invasive protrusions (Figure 7C,D). Mechanistically, HSF1A mimicked the effects of TRiC/CCT depletion by promoting YAP phosphorylation at S127 and suppressing phosphorylation of STAT3 at Y705, 4EBP1 at T37/46, and S6K1 at T389 (Figure 7E–G), thus attenuating YAP, STAT3, and mTOR signaling, corroborating the phenotypic outcomes of *CCT6A* and *CCT8* knockdown (Figure S20E, Supporting Information) and affirming the specificity of HSF1A for the TRiC/CCT complex.

To assess the efficacy of HSF1A in vivo, we intravenously injected 889DTC cells into nude mice and administered HSF1A (37.5 or 75 mg kg^−1^ daily for 6 days; *n* = 6/group) (Figure 7H). After 15 days, HSF1A‐treated mice exhibited a dose‐dependent reduction in lung tumors quantified using fluorescence microscopy (Figure 7I,J) and validated by H&E staining (Figure 7K,L). Notably, metastatic lesions were fewer and smaller in the treated mice than those in the controls (Figure 7J,L), with no acute toxicity observed (average weight loss <10%; Figure 7M). Taken together, these results demonstrate that the pharmacological inhibition of TRiC/CCT by HSF1A considerably suppresses lung cancer metastasis, revealing a promising therapeutic strategy.

### 2.8 DCAF12 Clinical Significance in LUAD

**Figure 8:**
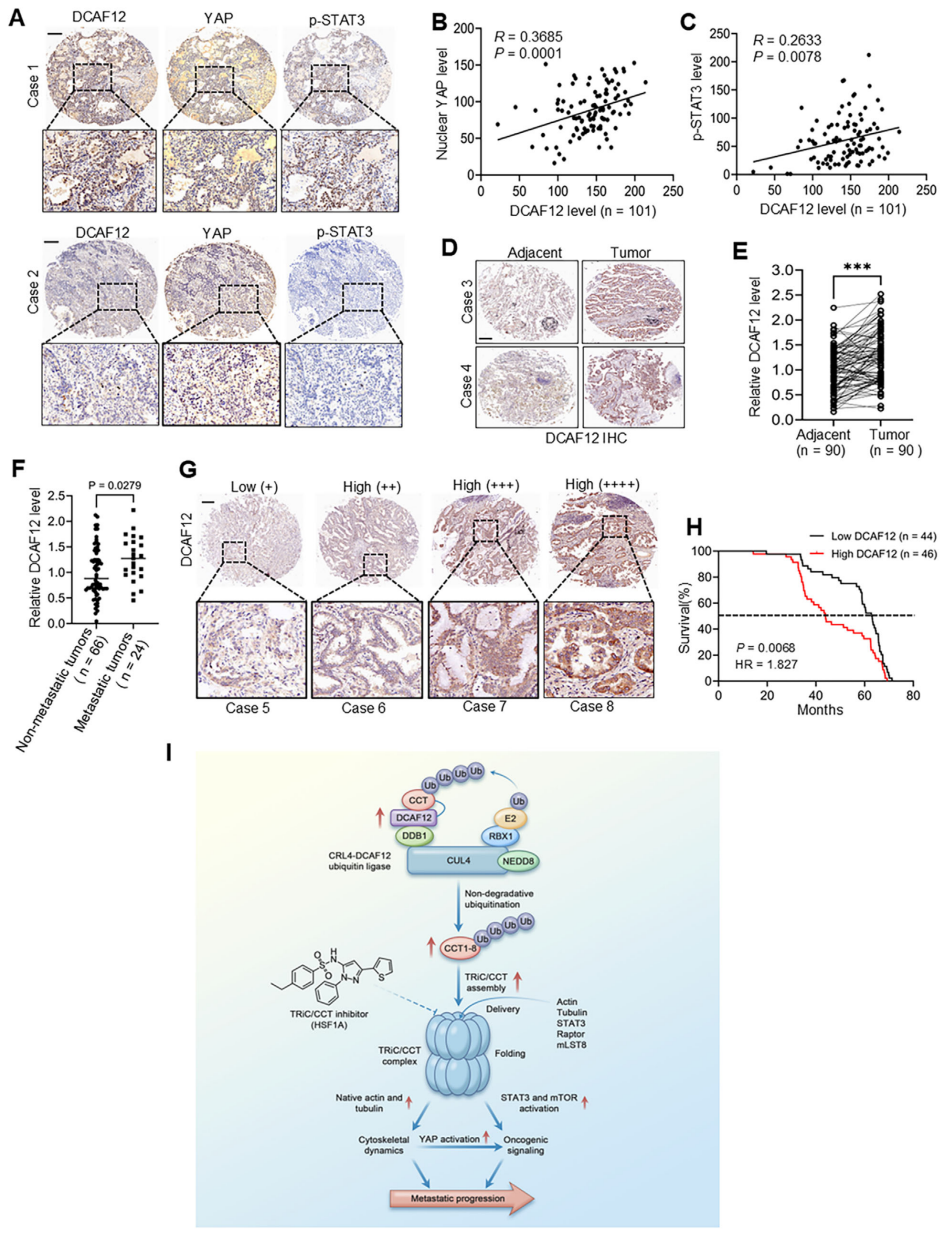
Prognostic significance of DCAF12 in LUAD. A) Representative immunohistochemical(IHC) staining for DCAF12, YAP, and phospho‐STAT3 (Y705) in patients with LUAD. Case 1 exhibited high DCAF12 expression, whereas Case 2 exhibited low expression. Scale bar: 200 µm. B,C) Protein correlation analysis in 101 patients with LUAD using Pearson's test, with the coefficients and *p*‐values indicated. D) IHC exhibited significantly higher DCAF12 levels in LUAD tissues than that in the adjacent normal tissues. Scale bar: 200 µm. E) Quantitative analysis confirmed DCAF12 upregulation in LUAD tissues. F) DCAF12 expression is elevated in metastatic and non‐metastatic LUAD tissues. G) Representative images indicating differential DCAF12 expression in tissue samples. Scale bar: 200 µm. H) Kaplan–Meier analysis revealed that high DCAF12 expression is correlated with worse overall survival in patients with LUAD. I) Proposed mechanistic model:  DCAF12 mediates non‐degradative ubiquitination of TRiC/CCT subunits, stabilizing the chaperonin complex and enhancing its folding capacity. This ubiquitination facilitates the conformational maturation of structural proteins (e.g., tubulin and actin), thereby improving cytoskeletal dynamics while concurrently activating signaling components (e.g., STAT3, Raptor, and mLST8) and initiating pro‐metastatic pathways, such as YAP, STAT3, and mTOR. By synchronously remodeling cellular architecture and amplifying oncogenic signaling, the DCAF12–TRiC/CCT axis drives metastatic progression in lung cancer. Consequently, treatment with the TRiC/CCT inhibitor HSF1A effectively suppressed tumor metastasis.

Our integrated multi‐omics analysis combining single‐cell RNA sequencing (scRNA‐seq) and tissue microarray profiling revealed the clinical significance of DCAF12 in LUAD pathogenesis. scRNA‐seq analysis of the GSE149655 dataset demonstrated cell type‐specific dysregulation, with tumor epithelial cells exhibiting the most significant upregulation of *DCAF12* compared to their normal counterparts (adjusted *p* < 0.001; Figure S21A–C, Supporting Information), suggesting that tumor cell‐autonomous mechanisms drive *DCAF12* dysregulation during malignant progression. Immunohistochemical validation of 101 LUAD specimens revealed the coordinated expression of DCAF12, YAP, and p‐STAT3 (Y705) (**Figure** **8**A). Quantitative analysis showed significant positive correlations between DCAF12 and nuclear YAP (*r* = 0.3685, *p* = 0.0001; Figure 8B), and between DCAF12 and p‐STAT3 (*r* = 0.2633, *p* = 0.0078; Figure 8C), indicating that DCAF12 is pivotal in the regulation of these oncogenic pathways.

Further validation in an independent cohort of 90 LUAD patients with survival data confirmed elevated DCAF12 expression in tumor tissues compared to that in adjacent non‐tumor tissues (*p* < 0.0001; Figure 8D,E), which was consistent with the findings from the public CPTAC dataset (Figure S22A, Supporting Information). Notably, metastatic tumors (*n* = 24) exhibited higher DCAF12 levels than non‐metastatic tumors (*n* = 66; *p* = 0.028; Figure 8F). Moreover, high DCAF12 expression was significantly associated with poor overall survival (*p* = 0.0068, HR = 1.827; Figure 8G,H), corroborating the results from the CPTAC dataset (Figure S22B, Supporting Information). Collectively, these findings establish DCAF12 as both a mechanistic hub for YAP/STAT3 signaling and a potential prognostic biomarker in LUAD, warranting further validation in larger cohorts.

## 3 Discussion

Our study identified DCAF12 as a crucial regulator of malignant dissemination through its dual control over cytoskeletal organization and oncogenic signaling. Comprehensive analyses demonstrated that genetic disruption of DCAF12 consistently reduced metastatic potential in preclinical models: inhibiting cell migration and tumorsphere formation in vitro, and decreasing metastatic burden in vivo. Mechanistic investigations revealed that DCAF12 maintained the integrity of the TRiC/CCT complex through non‐degradative ubiquitination, thereby preserving cytoskeletal organization while concurrently activating pro‐metastatic pathways, such as Hippo‐YAP, JAK‐STAT3, and mTOR (Figure 8I). This bifunctional mechanism elucidates the significant role of DCAF12 in metastasis and highlights its potential as a therapeutic target for advanced disease.

Despite evolutionary conservation, DCAF12 exhibited significant functional divergence across species. In *Drosophila*, DCAF12 regulates apoptosis through ubiquitin ligase‐independent cleavage of Diap1.^[^ ^40^ ^]^ In stark contrast, we uncovered a pro‐metastatic role for DCAF12 in human lung cancer, mediated through ubiquitin‐dependent regulation of the TRiC/CCT complex. Multiple lines of evidence support its oncogenic function: i) knockdown of *DCAF12* consistently impaired cell migration (Figure 1B,D; Figures S1G and S2B,E, Supporting Information) and tumorsphere formation (Figure 1G; Figure S4A,B, Supporting Information), whereas its overexpression restored these phenotypes (Figure 1F,H; Figure S3D, Supporting Information); ii) in murine models, *DCAF12* inhibition suppressed metastasis (Figure 1I–O); and iii) clinical studies associated DCAF12 overexpression with poor prognosis (Figure 8F–H; Figure S21A–C, Supporting Information). Notably, unlike its role in *Drosophila*, this cancer‐specific activity is fundamentally dependent on the ubiquitin ligase activity of DCAF12. This was evidenced by 1) the mediation of TRiC/CCT subunit ubiquitination (Figure 3A–C,H,I; Figure S7A–F, Supporting Information); 2) suppression of metastasis through mutations at ubiquitination sites or using blocking peptides (Figure 4I–M; Figure S17G–I, Supporting Information); and 3) the therapeutic efficacy of disrupting the DCAF12‐TRiC/CCT interaction in vivo (Figure 4M). These findings establish DCAF12 as a context‐dependent molecular switch in cancer progression, highlighting its novel role in promoting lung cancer metastasis through TRiC/CCT ubiquitination.

The evolutionary repurposing of the molecular function of DCAF12, from apoptosis regulation in *Drosophila* to ubiquitin‐dependent metastasis promotion in humans, prompted us to investigate its cancer‐specific mechanism. We established a novel regulatory paradigm for the TRiC/CCT chaperonin complex through DCAF12‐mediated non‐degradative ubiquitination. While previous reports suggested that DCAF12 targets unassembled CCT5 monomers for proteasomal degradation,^[^ ^56^, ^57^ ^]^ our data support a distinct model in which DCAF12 specifically catalyzes stabilization‐linked ubiquitination of all TRiC/CCT subunits. This conclusion was supported by three independent lines of evidence. First, contrary to the degradation model, neither genetic depletion of DCAF12 nor pharmacological inhibition of CRL4 activity resulted in subunit accumulation. Instead, these interventions moderately decreased the subunit stability (Figure 3D,E; Figure S10B, Supporting Information), whereas DCAF12 overexpression did not reduce subunit levels (Figure S10A, Supporting Information). Second, quantitative pulse‐chase analyses revealed that DCAF12 significantly prolonged the half‐life of the core subunits (Figure 3F,G; Figure S11A,B, Supporting Information). Third, comprehensive ubiquitin profiling identified K63‐ and K27‐linked polyubiquitin chains on TRiC/CCT subunits (Figure 3H,I; Figure S12, Supporting Information). These linkage types have been primarily associated with structural stabilization and protein complex regulation,^[^ ^46^, ^47^, ^48^ ^]^ in direct contrast to K48‐linked chains that signal for proteasomal degradation.

To evaluate the physiological relevance of ubiquitination‐dependent stabilization, we assessed its functional impact on cellular homeostasis. Our quantitative assessments revealed that disrupting DCAF12‐mediated ubiquitination led to widespread protein aggregation (Figure 5A–C), underscoring its critical role in maintaining protein stability and homeostasis. Structural analyses further established the necessity of DCAF12 to preserve the quaternary structure of the TRiC/CCT complex (Figure 5E–I), which is consistent with our observations of extended subunit half‐lives (Figure 3F,G; Figure S11A,B, Supporting Information). These results collectively support a model in which DCAF12 enhances the stability of functional TRiC/CCT holoenzymes through non‐degradative ubiquitination, revealing an unprecedented regulatory mechanism of chaperonin modulation that likely facilitates inter‐subunit stabilization and optimizes the chaperonin cycle. While our present study provides comprehensive support for this model, future high‐resolution structural studies are critical to fully delineate the atomic details of this regulatory mechanism and to explore its implications in metastatic progression.

Our study identified DCAF12 as a central signaling integrator in lung cancer metastasis, bridging cytoskeletal dynamics with oncogenic pathways through its regulation of the TRiC/CCT chaperonin complex. By ensuring proper folding of actin and tubulin, the DCAF12‐TRiC/CCT axis maintains cytoskeletal structural integrity, which is essential for cell motility.^[^ ^51^, ^52^, ^58^ ^]^ Downregulation of DCAF12 disrupts this balance, leading to tubulin destabilization, impaired actin polymerization, and defective formation of migratory structures, such as filopodia (Figure 6A–E), suggesting that DCAF12 regulates cytoskeletal remodeling, which is indispensable for metastatic dissemination. Notably, the role of DCAF12 extends beyond structural regulation to encompass mechanosensitive signaling. We demonstrated that DCAF12 activated YAP (Figure 6F‐K; Figure S20B,C, Supporting Information) linking cytoskeletal tension to transcriptional reprogramming. This dual control coordinating mechanical motility (via cytoskeletal dynamics) and YAP‐driven gene expression suggests a novel feedforward loop that coordinates metastatic progression. Furthermore, the interaction of DCAF12 with the TRiC/CCT complex enables quality control over key oncogenic effectors, including STAT3 and mTOR components (Raptor and mLST8),^[^ ^22^, ^23^ ^]^ activating the STAT3 and mTOR pathways, which are essential for cellular proliferation, migration, and stemness.^[^ ^59^, ^60^ ^]^ While our study focused on cytoskeletal and YAP/STAT3/mTOR pathways, previous studies have implicated DCAF12 in TGF‐β signaling in other cancers.^[^ ^39^ ^]^ Given the role in metastasis,^[^ ^61^ ^]^ this conserved regulatory mechanism may further augment the pro‐metastatic function of DCAF12 in lung cancer. The tissue‐invariant nature of these interactions highlights that DCAF12 is an evolutionary hub for cell motility and stemness. Therapeutically, our results suggest that targeting DCAF12 is more effective than inhibiting the individual downstream effectors. As a chaperone regulator, upstream of multiple pathways, it disrupts the integrated oncogenic network that drives metastasis. Future studies should investigate the interaction of DCAF12 with TGF‐β and other context‐dependent partners to fully understand its therapeutic potential.

Building on the complex role of DCAF12 in regulating metastasis, we aimed to exploit its TRiC/CCT‐mediated protein‐folding activity as a therapeutic target. Pharmacological inhibition of the DCAF12‐TRiC/CCT axis by HSF1A recapitulated the anti‐metastatic effects observed with genetic depletion or ubiquitination‐blocking peptides (Figure 4L,M; Figure S17G–I, Supporting Information). Notably, HSF1A treatment suppressed the metastatic potential in in vitro and in vivo systems (Figure 7A–L), reflecting the phenotypic outcomes of the DCAF12 pathway inhibition through other modalities. Consistent results from genetic, peptide‐based, and pharmacological interventions strongly validated the DCAF12‐TRiC/CCT axis as a promising therapeutic target. Our findings demonstrate that disrupting this pathway through various approaches consistently suppresses metastasis and exhibits robust and reproducible biological effects. This convergence of evidence confirms the central role of DCAF12‐mediated TRiC/CCT regulation in cancer progression and offers multiple potential avenues for clinical intervention.

Our study identified a pharmacologically exploitable window for inhibiting the TRiC/CCT chaperonin complex in cancer, thereby revealing a therapeutic strategy that leverages the differential dependence of tumor cells on chaperonin function. We demonstrated that while complete ablation of TRiC/CCT is generally lethal, partial disruption—achieved through DCAF12 inhibition, CCT5‐K89R mutagenesis, or ubiquitination‐blocking peptides—reduces functional complex assembly by ≈70% (Figure 5G–I) without affecting cell viability (Figures S1H,S2F,S14B,S15C, and S17J, Supporting Information). This suggests that tumors can endure significant deficits in folding capacity, as long as a minimal activity threshold (≈30% residual complex or less) is maintained. This threshold effectively decouples global proteostasis from metastasis‐specific processes, allowing for the selective targeting of malignant dissemination. Importantly, the anti‐metastatic effects are mechanistically distinct from general cytotoxicity, as evidenced by three converging lines of evidence: all interventions (DCAF12 inhibition, the CCT5‐K89R knock‐in, and ubiquitination‐blocking peptides) specifically suppressed migration and tumorsphere formation without compromising basal cell viability; genetic complementation confirmed that wild‐type Cct5, but not the assembly‐defective K89R mutant, rescued metastatic capacity without altering proliferative potential (Figure 4E–G; Figure S14B, Supporting Information); and consistent specificity across genetic, peptide, and pharmacological modalities confirmed that this pathway specifically regulates cytoskeletal remodeling critical for migration/invasion, independent of bulk protein homeostasis. The favorable safety profile of HSF1A underscores the translational potential of targeting this axis to impair metastasis while preserving normal proteostatic function, although further studies in advanced models are essential to confirm long‐term efficacy and anticipate potential resistance mechanisms.

In conclusion, our study establishes DCAF12 as a primary regulator of lung cancer metastasis through a dual mechanism: stabilizing the TRiC/CCT complex via non‐degradative ubiquitination to maintain cytoskeletal dynamics, and concurrently activating the YAP, STAT3, and mTOR pathways. The evolutionary repurposing of DCAF12, from apoptosis regulation in *Drosophila* to ubiquitin‐dependent metastasis promotion in humans, underscores its context‐dependent versatility. Clinically, DCAF12 overexpression correlates with pathway activation and poor prognosis, whereas its genetic or pharmacological targeting selectively inhibits metastasis without compromising cell viability. Although our findings reveal a promising therapeutic option, future studies should address the long‐term efficacy and potential crosstalk with TGF‐β signaling. By bridging chaperone‐mediated protein folding with oncogenic signaling, this study redefines DCAF12 as a central orchestrator of metastasis through ubiquitin‐dependent chaperone regulation, revealing new therapeutic opportunities for targeting metastatic disease.

## 4 Experimental Section

### Reagents

The details of key antibodies and reagents are presented in Table S1 (Supporting Information).

### Cell Culture

The 889DTC cell line, derived from a *K‐ras* ^G12D/+^; *Trp53* ^−/−^; and *tdTomato* genetically engineered mouse model of lung adenocarcinoma from the Winslow laboratory at Stanford University, CA, USA. It has high metastatic potential through subcutaneous inoculation and is a unique model for studying disseminated tumors.^[^ ^45^ ^]^ Although a Research Resource Identifier (RRID) was unavailable, its authenticity was confirmed by the source laboratory.

All human cell lines, HEK293T (RRID: CVCL_0063), A549 (RRID: CVCL_0023), H460 (RRID: CVCL_0459), and H1299 (RRID: CVCL_0060), were obtained from the Cell Bank of the Chinese Academy of Sciences (Shanghai, China). Cell line identities were verified by commercial short tandem repeat profiling with database matching, confirming the absence of cross‐contamination.

All cell lines were maintained at 37 °C with 5% CO_2_ in appropriate media supplemented with 10% fetal bovine serum (FBS) and were tested monthly for mycoplasma contamination using the MycoBlue Detector (Vazyme Biotech, Nanjing, China). All experiments used cells within 10 post‐thaw passages to maintain phenotypic stability.

### Gene Knockdown

siRNA was transfected using Lipofectamine RNAiMAX (Invitrogen, Waltham, MA, USA). Knockdown efficiency was assessed 72 h post‐transfection using quantitative reverse transcription PCR (qRT‐PCR; normalized to *GAPDH*) and immunoblotting. For mouse *Dcaf12* knockdown in 889DTC cells, shRNAs were designed using SplashRNA ^[^ ^62^ ^]^ and cloned into the LT3GEPIR Tet‐On lentiviral system (Addgene Watertown, MA, USA [#111177]). Transduced cells were selected using puromycin and induced with 1 µg mL^−1^ doxycycline for 72 h. All the oligonucleotide sequences are listed in Table S2 (Supporting Information).

### qRT‐PCR

Total RNA was isolated from cancer cells using TRIzol reagent (Invitrogen) and reverse‐transcribed into cDNA. qRT‐PCR was performed using SYBR Green Master Mix (Thermo Fisher Scientific) on a QuantStudio system. Gene expression levels were normalized to *GAPDH*, and the gene‐specific primer sequences are provided in Table S2 (Supporting Information).

### Plasmid Constructs

cDNA sequences encoding mouse Dcaf12, human DCAF12, and all TRiC/CCT subunit genes were cloned into plasmid vectors using a Seamless Cloning Kit (Beyotime Biotechnology, Shanghai, China). The following expression constructs were generated: Lenti‐FLAG‐DCAF12, pcDNA3 × FLAG‐DCAF12, and pcDNA3 × HA‐TCP1/CCT2‐8, along with their respective site‐directed mutants. These constructs were based on lentiviral (Lenti‐EFS‐FLAG) or mammalian expression vectors (pcDNA3 × FLAG and pcDNA3 × HA), each containing an N‐terminal HA or FLAG epitope tag. Table S1 (Supporting Information) presents a complete list of the constructs.

### CRISPR‐Cas9‐Mediated Genome Editing

To label the endogenous *DCAF12* gene with an N‐terminal HA epitope, a single‐guide RNA (sgRNA) targeting the region proximal to the *DCAF12* start codon was designed and cloned into the pLX‐sgRNA vector (Addgene #50662). The homology‐directed repair (HDR) template, constructed in pUC18, contained two 500‐bp homology arms flanking the *DCAF12* locus, an HA‐tag coding sequence, and a T2A‐puromycin resistance cassette (flanked by LoxP sites). A549 cells were co‐transfected with pLX‐sgDCAF12, pGL3‐Cas9, and the HDR template. Puromycin selection (2 µg mL^−1^) was initiated 48 h post‐transfection and maintained until the emergence of resistant colonies. Individual clones were expanded, and HA‐tag integration was validated using immunoblotting with an anti‐HA antibody.

To generate *DCAF12* knockout cells, A549 cells were transduced with LentiCRISPRv2 vectors expressing either *DCAF12*‐targeting sgRNA (Lenti‐sgDCAF12) or non‐targeting *EGFP* sgRNA (Lenti‐sgEGFP; control). After transduction, the cells were selected using puromycin selection (1.5 µg mL^−1^) for 7 days, followed by single‐cell dilution cloning in 96‐well plates (Thermo Fisher Scientific). Clonal lines were screened for *DCAF12* knockout efficiency using immunoblotting using a DCAF12‐specific antibody. Deficiency of the DCAF12 protein confirmed the successful knockout. sgRNA sequences used in the study are listed in Table S2 (Supporting Information).

To introduce the *CCT5* K89R mutation (AAG→CGG), an sgRNA targeting the *CCT5* K89 locus (5′‐TCAGATTGCCAAGCTGATGG‐3′) and a 500‐nt single‐stranded oligonucleotide donor (ssODN) were designed. The ssODN contained a K89R substitution, PAM‐disrupting mutation (TGG→TAG), and restriction site screening marker (CTGAAT→AGCTAG). A549 cells were co‐transfected with 1 µg ssODN, 0.5 µg sgRNA plasmid, and 1 µg Cas9 expression plasmid using lipofectamine stem transfection reagent (Thermo Fisher, STEM00003). Single‐cell clones were isolated 72 h post‐transfection, and successful knock‐in was verified using PCR and Sanger sequencing.

### Migration Assay

For the Transwell migration assay, 4 × 10⁴ cancer cells in 100 µL of serum‐free medium were seeded in the upper chamber of Transwell inserts (8 µm pore size; Corning). The lower chamber was filled with medium supplemented with 15% FBS as a chemoattractant. After incubation for 6–24 h (cell type‐dependent), the non‐migrating cells on the upper membrane surface were removed by gentle swabbing. For tdTomato expressing 88CDTC cells, migration was determined using fluorescence microscopy. For H1299, A549, and H460 cells, the inserts were fixed with 100% methanol, rinsed with phosphate‐buffered saline (PBS), and stained with 0.5% crystal violet. The migrated cells were imaged under bright‐field microscopy and quantified using ImageJ software.

For the wound healing assay, cells were seeded in 6‐well plates and grown to confluence. A sterile pipette tip was used to create a straight scratch on the monolayer. After rinsing the wells with PBS to remove detached cells, images of the scratch area were taken to document the initial “zero‐hour” time point. The healing process was monitored at various time intervals, depending on the cell type. Wound closure was quantified by measuring the distance between the wound edges using the ImageJ software.

### Clonogenic Survival Assay

Cells were seeded into 60‐mm dishes or 6‐well plates at densities ranging from 500 to 1000 cells/well, depending on the cell type, as previously described.^[^ ^63^ ^]^

### Tumor Sphere Formation Assay

Cancer cells were treated with 0.05% trypsin to generate a cell suspension, and subsequently seeded into 24‐well ultra‐low attachment plates at 2 × 10^3^ cells/well, as previously described.^[^ ^63^ ^]^

### Mass Spectrometry Analysis

To identify DCAF12‐interacting proteins, tdTomato expressing 88CDTC cells stably expressing FLAG‐tagged Dcaf12 were cultured to 80% confluence and treated with 2 mm DSP for crosslinking. After two PBS washes, cells were lysed in NP‐40 buffer (50 mm Tris‐HCl [pH 7.4], 150 mm NaCl, and 1% NP‐40) supplemented with a complete protease inhibitor cocktail (Roche, Indianapolis, IN, USA). Lysates were centrifuged at 10 000 × g for 10 min at 4°C, and the supernatants were incubated with anti‐FLAG M2 affinity beads (Sigma–Aldrich, St. Louis, MO, USA) for IP(IP). After washing extensively with PBS, bound proteins were eluted and processed for liquid chromatography‐tandem mass spectrometry (LC‐MS/MS) analysis.

### IP and Immunoblot Analysis

The cells were lysed in NP‐40 buffer supplemented with a complete protease inhibitor cocktail (Roche). Protein concentrations were determined using the bicinchoninic acid (BCA) assay, and equal amounts (typically 500–1000 µg) were used for IP. For affinity purification, clarified lysates were incubated with either anti‐FLAG M2 magnetic beads (Sigma–Aldrich) or Pierce Anti‐HA Magnetic Beads (Thermo Fisher Scientific) for 1 h at 25 °C with gentle rotation. For antibody‐based IP, the samples were incubated overnight at 4 °C with specific antibodies (1–2 µg) conjugated to Protein A/G Sepharose beads (MedChemExpress, Monmouth, NJ, USA). Immune complexes were washed five times with Tris‐buffered saline containing 0.1% Tween‐20 (TBST), eluted by boiling in 1 × Laemmli sample buffer, determined using SDS‐PAGE using 4–12% Bis‐Tris gradient gels (Smart‐Lifesciences, Changzhou, China), and transferred to polyvinylidene difluoride (PVDF) membranes for immunoblotting with the indicated primary antibodies.

### GST Pull‐Down Assay

In the glutathione S‐transferase (GST) pull‐down assay, GST‐fusion proteins bound to glutathione beads were incubated with specific proteins in pull‐down buffer ([Invitrogen] 50 mm Tris‐HCl [pH 7.4], 150 mm NaCl, and 0.5% Triton X‐100) for 4 h at 4 °C. After incubation, the input‐ and bead‐bound proteins were analyzed using an immunoblotting assay.

### In Vivo Ubiquitination Assay

HEK293T cells were transfected with 6 × His‐ubiquitin and other plasmids using Lipofectamine 2000 (Invitrogen). After 48 h, the cells were lysed in denaturing buffer (6 m guanidine‐HCl, 100 mm Na_2_HPO_4_/NaH_2_PO_4_, pH 8.0, 10 mm imidazole). The lysates were sonicated and incubated with Ni‐NTA beads for 3 h at 25 °C to capture ubiquitinated proteins. After washing, the proteins were eluted and analyzed by immunoblotting using the indicated antibodies.

### Protein Half‐Life Assay

The half‐life of the target protein was assessed using a CHX chase assay. Cells were treated with CHX (50 µg mL^−1^) and harvested at the indicated time points (0, 3, 6, 9, and 12 h). Whole‐cell lysates were prepared and analyzed using immunoblotting. Protein levels were quantified using ImageJ, normalized to a loading control, and plotted over time to determine degradation kinetics.

### Protein Aggregation Detection Assay

Protein aggregation was assessed using the PROTEOSTAT Aggresome Detection Kit (Enzo Life Sciences, Farmingdale, NY, USA), following the manufacturer's protocol. Briefly, cells seeded on glass slides were washed with PBS, fixed with 4% formaldehyde for 30 min at 25°C, and permeabilized with permeabilization solution (0.5% Triton X‐100, 3 mm EDTA) for 30 min on ice with gentle agitation. After permeabilization, cells were stained with PROTEOSTAT dye (1: 20 000 dilution) for 30 min at 25 °C. Protein aggregates were visualized and quantified by measuring red fluorescence using a fluorescence microscope.

### Blue Native Gel Analysis of the TRiC/CCT Complex

Cells were lysed in NativePAGE Sample Buffer (Invitrogen) supplemented with protease inhibitors by repeated passage through a 27‐gauge needle. The lysates were centrifuged at 12 000 × g for 10 min, and 50 µg of total protein was resolved on NativePAGE Novex Bis‐Tris gels (Invitrogen). Electrophoresis was performed using NativePAGE Running Buffer in the anode chamber, initially at 150 V for 60 min, followed by 250 V for 90 min. For immunoblotting, proteins were denatured by incubating the gels in a transfer buffer containing SDS for 30 min prior to semi‐dry transfer onto a PVDF membrane at 400 mA for 3 h. The membranes were probed with primary antibodies against CCT3, CCT5, or CCT8. As a control, parallel SDS‐PAGE analysis was performed to verify the total expression levels of individual CCT subunits using β‐actin or GAPDH as loading controls.

### In Vivo F‐Actin/G‐Actin Assay

The in vivo G‐actin/F‐actin ratio assay was performed on living cells using the G‐actin/F‐actin in vivo assay Kit (Cytoskeleton, St. Denver, CL, USA), following the manufacturer's protocol. Upon reaching 80% confluence, the cells were lysed in F‐actin stabilization buffer containing 2 mm ATP and protease inhibitors and then incubated at 37 °C for 10 min. The lysates were centrifuged at 100 000 × g (4 °C) for 1 h to separate the G‐actin (supernatant) from the F‐actin (pellet). The F‐actin‐containing pellet was resuspended in the depolymerization buffer and incubated on ice for 1 h. Equal volumes of G‐ and F‐actin lysates were analyzed using SDS‐PAGE and immunoblotted with an anti‐actin antibody. G‐ and F‐actin levels were quantified using ImageJ software, and the F‐actin‐to‐G‐actin ratio was calculated.

### Phalloidin Staining

For phalloidin staining, cancer cells transfected with siRNA for 48 h were seeded onto coverslips and cultured for 24 h at 37°C. The cells were fixed with 4% paraformaldehyde for 15 min at 25 °C, permeabilized with 0.3% Triton X‐100 for 10 min, and blocked with 1% bovine serum albumin for 30 min. Subsequently, the cells were stained with phalloidin‐Alexa Fluor 594 (1:200 dilution, 40 min) and Hoechst 33342 (5 µg mL^−1^, 5 min) (Beyotime). Visualization and imaging were performed using a fluorescence microscope, in which filopodia and slender, elongated protrusions (characterized by thin, straight structures) were observed.^[^ ^64^ ^]^

### Immunofluorescence Assay

Cells grown on coverslips were fixed with 4% paraformaldehyde, permeabilized with 0.1% Triton X‐100, and blocked with 15% FBS in PBS. Subsequently, the coverslips were incubated overnight at 4 °C with primary antibodies against YAP, washed, and incubated for 1 h at 25 °C with fluorescently labeled secondary antibodies. The nuclei were counterstained with Hoechst 33342. Fluorescence images were captured using a high‐resolution epifluorescence microscope under standardized exposure conditions to avoid signal saturation. For colocalization analysis, background‐subtracted images were processed using ImageJ software. Quantitative colocalization was assessed by calculating Pearson's correlation coefficient (PCC) using the Coloc2 plugin and applying thresholds to exclude nonspecific background signals. To ensure statistical robustness, a minimum of 50 cells per experimental condition were analyzed for each experiment.

### In Vivo Metastasis Studies

All animal experiments were conducted in accordance with protocols approved by the Institutional Animal Care and Use Committee of Shanghai Pulmonary Hospital (Approval #K23‐269). Four distinct metastatic assays were performed using female athymic nude mice (6–8 weeks old, 18–20 g).

For the tail vein injection model, 889DTC cells were transiently transfected with either pooled *Dcaf12* siRNAs (experimental group) or control siRNA (*n* = 5/group). After 24 h, 1 × 10⁵ cells suspended in 200 µL PBS were injected intravenously. Mice were humanely euthanized using CO_2_ asphyxiation 1 week post‐injection, and pulmonary metastases were quantified using fluorescence dissection microscopy of dTomato‐positive tumor foci.

In the subcutaneous tumor model, 5 × 10⁴ 889DTC cells expressing Tet‐on‐regulated shLacZ, shDcaf12‐1#, or shDcaf12‐2# were injected into the right flanks (*n* = 5/group). Primary tumors were surgically removed 10 days post‐injection following established protocols.^[^ ^45^ ^]^ Mice then received doxycycline (1 µg mL^−1^ in drinking water, replaced every 3 days) to induce knockdown. Furthermore, 5 weeks post‐injection, the metastatic burden in the lungs and liver was assessed using fluorescence dissection microscopy and hematoxylin and eosin (H&E) staining.

To evaluate the impact of CCT5 ubiquitination inhibition on metastasis, 2 × 10⁶ H1299 cells (in 100 µL PBS) expressing either the Tet‐on‐regulated CCT5‐2W‐2 blocking peptide or EGFP control were injected intravenously (*n* = 5/group). The mice were administered doxycycline, as described above, to induce expression. Lung metastases were examined using fluorescence dissection microscopy after 6 weeks.

For pharmacological inhibition studies, mice receiving 5 × 10⁴ 889DTC cells intravenously (*n* = 6/group) were treated with either vehicle (5% DMSO in corn oil) or HSF1A (37.5 or 75 mg kg^−1^) for 6 days within a 15‐day window. Body weight was monitored every alternate day for toxicity assessment. Therapeutic efficacy was determined using fluorescence dissection microscopy and H&E staining of lung tissues at the study endpoint.

### IHC

(LUAD) tissue microarray (TMAs) was obtained from Outdo Biotech, Shanghai, China. IHC was performed using the IHC Kit D (Servicebio, Wuhan, China) with primary antibodies targeting DCAF12, YAP, and phosphorylated STAT3 (p‐STAT3). Following staining, all the sections were digitally scanned using a 3D HISTECH whole‐slide imaging system (3DHISTECH, HISTECH Ltd., Budapest, Hungary). Protein expression was quantitatively analyzed using the AIpathwell intelligence‐based digital pathology platform (Servicebio). Modified H‐scores were calculated using the following standard formula:

H‐score = (% weak intensity × 1) + (% moderate intensity × 2) + (% strong intensity × 3).^[^ ^65^ ^]^ This semi‐quantitative scoring system was used to standardize the evaluation of DCAF12, YAP, and p‐STAT3 expression levels across all TMA specimens.

### Statistical Analysis

All in vitro experiments were performed in at least two independent replicates, unless otherwise specified. Data are presented as the mean ± standard deviation. Statistical analyses were performed using the GraphPad Prism (v9.0). For comparisons between two groups, an unpaired two‐tailed Student's *t*‐test (assuming equal variance) was applied. Multiple group comparisons were analyzed using a one‐way analysis of variance with Tukey's post‐hoc test. Statistical significance was set at *p* < 0.05.

## Conflict of Interest

The authors declare no conflict of interest.

## Author Contributions

Z.W., H.H., and K.H. contributed equally to this work. DPW, YPX, and JYC conceived and designed the study. ZYW, HHH, and KZH performed most experiments and conducted data analysis. XWC, ZZ, and ZC contributed to plasmid construction and validation. HC and RYL carried out database mining and IHC analyses. WLJ and SC provided critical reagents and participated in animal experiments. The manuscript was drafted by DPW and revised collaboratively by DPW, YPX, and JYC. All authors reviewed, edited, and approved the final version of the manuscript.

## Supporting information



Supporting Information

Supplementary Table 1

Supplementary Table 2

## Data Availability

The data that support the findings of this study are available from the corresponding author upon request.
