# Supplementary material for: DCAF12 Ubiquitin Ligase Promotes Lung Cancer Metastasis by Modulating the TRiC/CCT Chaperonin Complex
Source: Adv Sci (Weinh). 2025 Oct 5;13(3):e09695. doi: 10.1002/advs.202509695 (PMC12806248; doi:10.1002/advs.202509695)
Supplement: Supplementary file 1 — Supporting Information [file ADVS-13-e09695-s003.docx]

**Supplementary data**


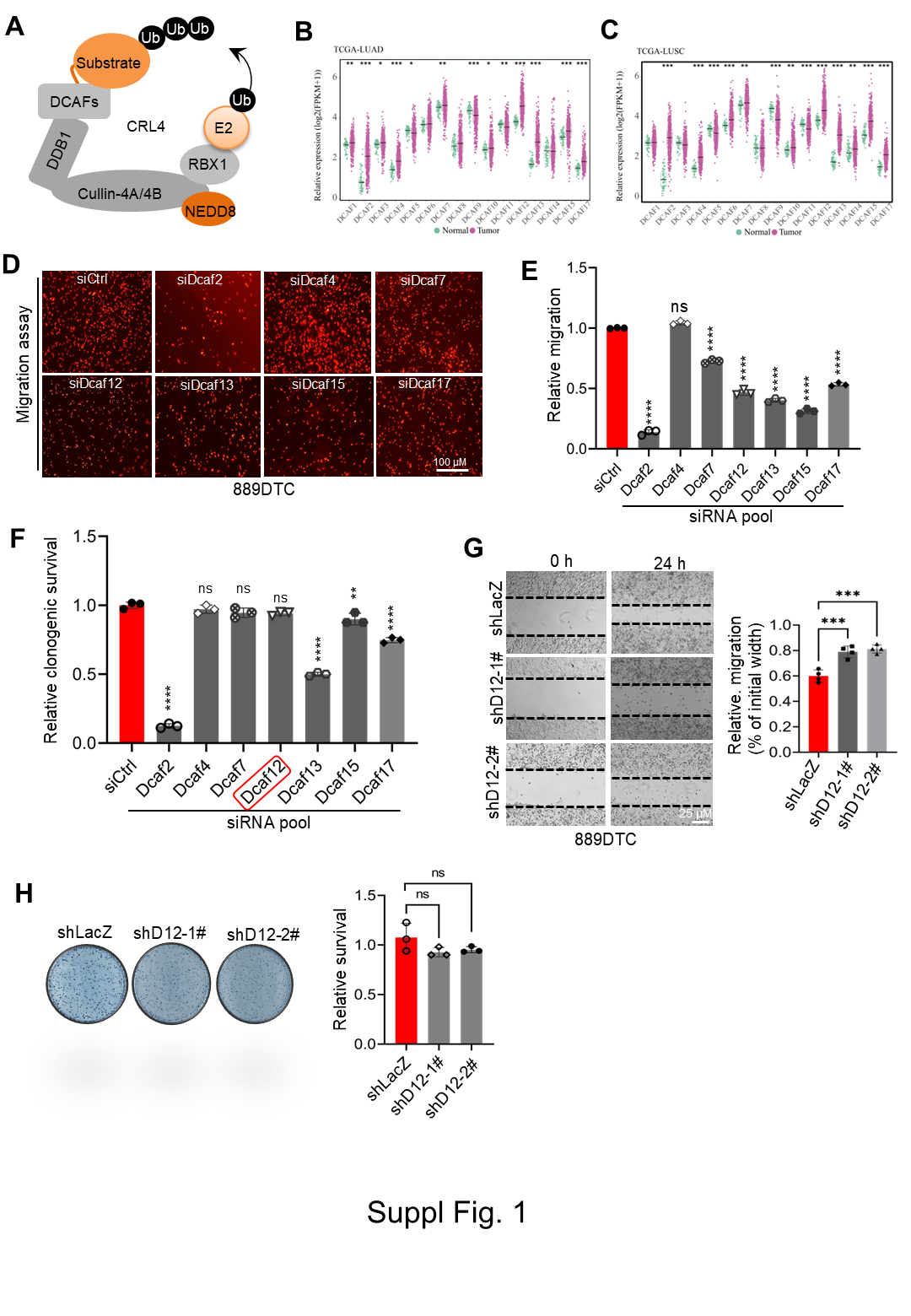


**Fig. S1. The DCAF family regulates 889DTC cell migration and survival.**(**A**) Schematic representation of the CRL4-DCAF substrate recognition.

 (**B,C**) DCAF expression profiles in LUAD (B) and LUSC (C) from TCGA database.

(**D-F**) siRNA knockdown of Dcaf family members (*Dcaf2/4/7/12/13/15/17*) significantly reduced 889DTC migration (transwell; D,E) with differential survival effects (clonogenic; F).

(**G,H**) *Dcaf12* knockdown (shRNA) suppressed migration (wound healing; G) but not survival (clonogenic; H). Data represent the mean ± SD (n = 3-4 technical replicates per condition; representative of two independent experiments; one-way ANOVA with Tukey's post-hoc test, *P* < 0.01, *P < 0.001,* ****P* < 0.0001; ns, not significant).


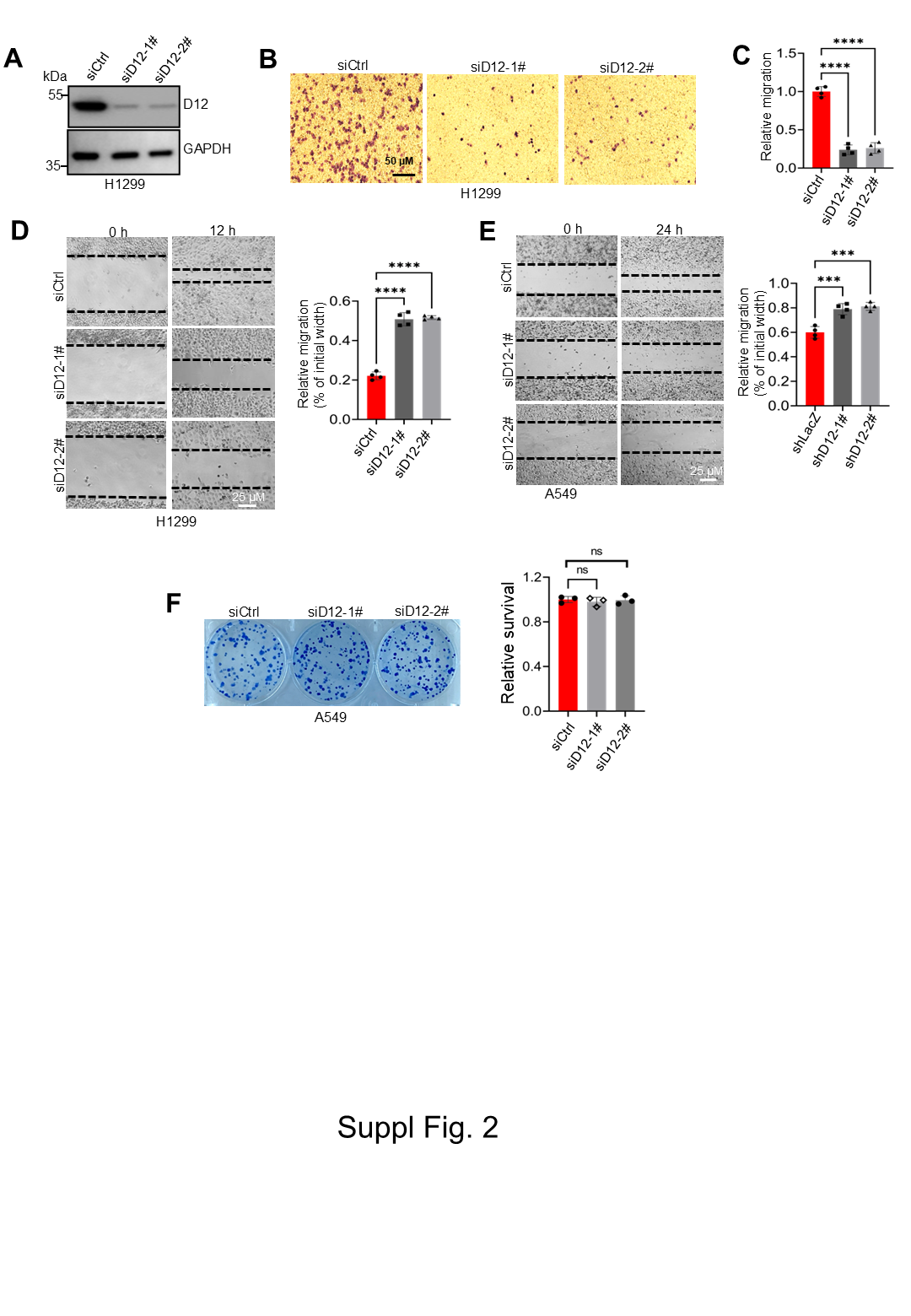


**Fig. S2. *DCAF12* knockdown impairs lung cancer cell migration with limited survival effects.**

(**A-C**) siRNA-mediated *DCAF12* knockdown significantly reduced H1299 cell migration (Transwell assay). (**D,E**) Impaired wound closure in DCAF12-depleted H1299 (D) and A549 (E) cells (transfected with). (**F**)  Minimal survival effect of *DCAF12* knockdown in A549 cells (clonogenic assay). Data represent mean ± SD (n = 3-4 technical replicates per condition; representative of two independent experiments; one-way ANOVA with Tukey's post-hoc test, ****P* < 0.001, *****P* < 0.0001; ns, not significant). Ctrl: control; D12: DCAF12.


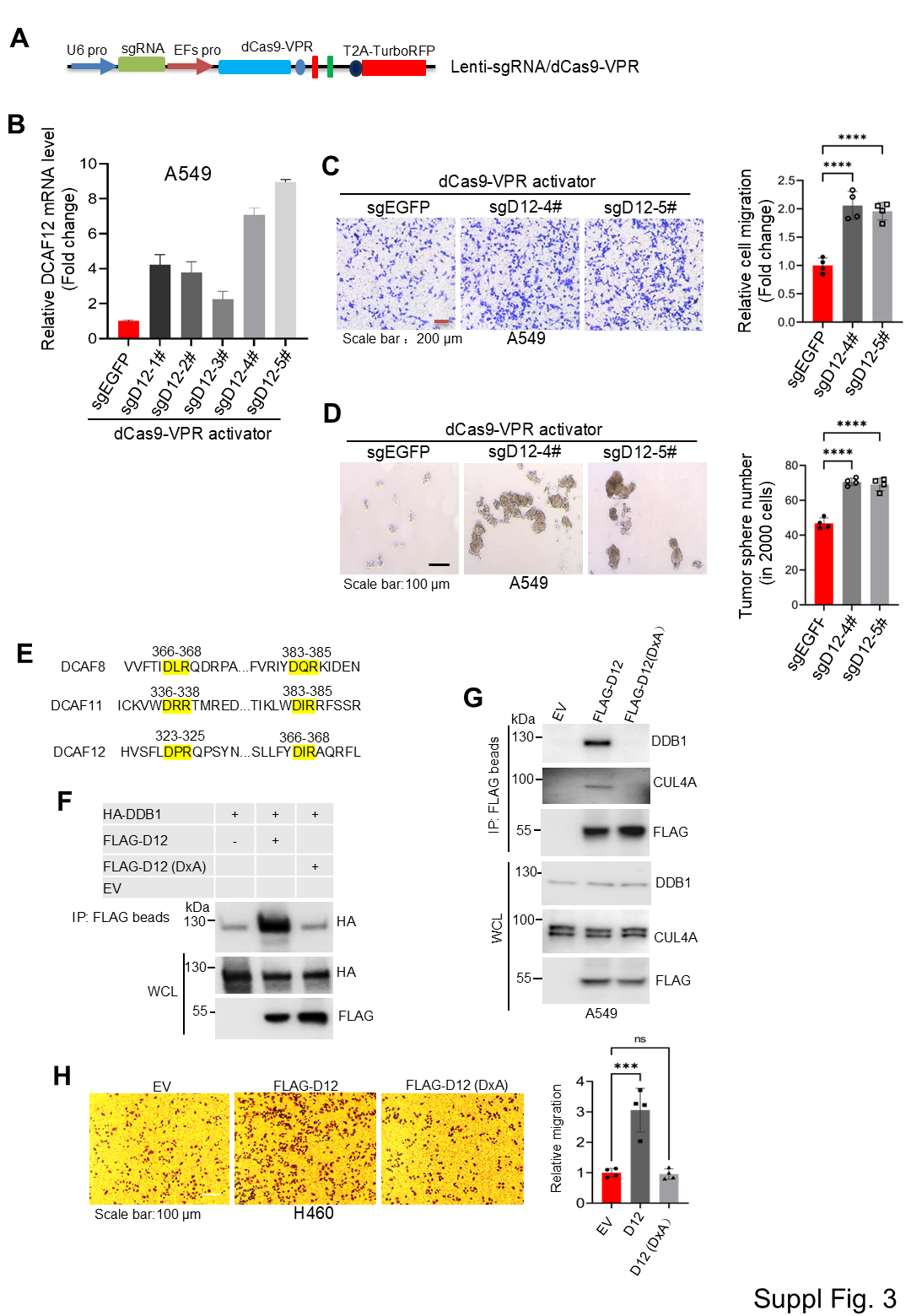


**Fig. S3. DCAF12 promotes cell migration by interacting with the CRL4 complex.**(**A**) Schematic diagram of the lentivirus-delivered CRISPRa system (dCas9-VPR) used for gene activation. (**B**) qPCR analysis of DCAF12 mRNA levels in A549 cells after CRISPRa-mediated activation. (**C**) Enhanced migration of A549 cells following CRISPRa-induced DCAF12 upregulation. (**D**) Increased tumorsphere formation capacity of A549 cells upon DCAF12 activation using the CRISPRa system. (**E**) Evolutionary conservation of the CRL4-binding double DxR-box motif (highlighted in yellow) in DCAF12. (**F, G**) Disruption of the DCAF12–DDB1/CUL4A interaction by the DxR box mutation, as evaluated by co-IP in HEK293T (F) and A549 (G) cells. (**H**) Expression of the DCAF12 DxR mutant impairs the migration ability of H460 cells compared to wild-type DCAF12. Data are presented as mean ± SD (n = 4 technical replicates per condition from one representative experiment of two independent biological replicates; one-way ANOVA with Tukey’s post hoc test; ****P* < 0.001, *****P* < 0.0001; ns, not significant).

**
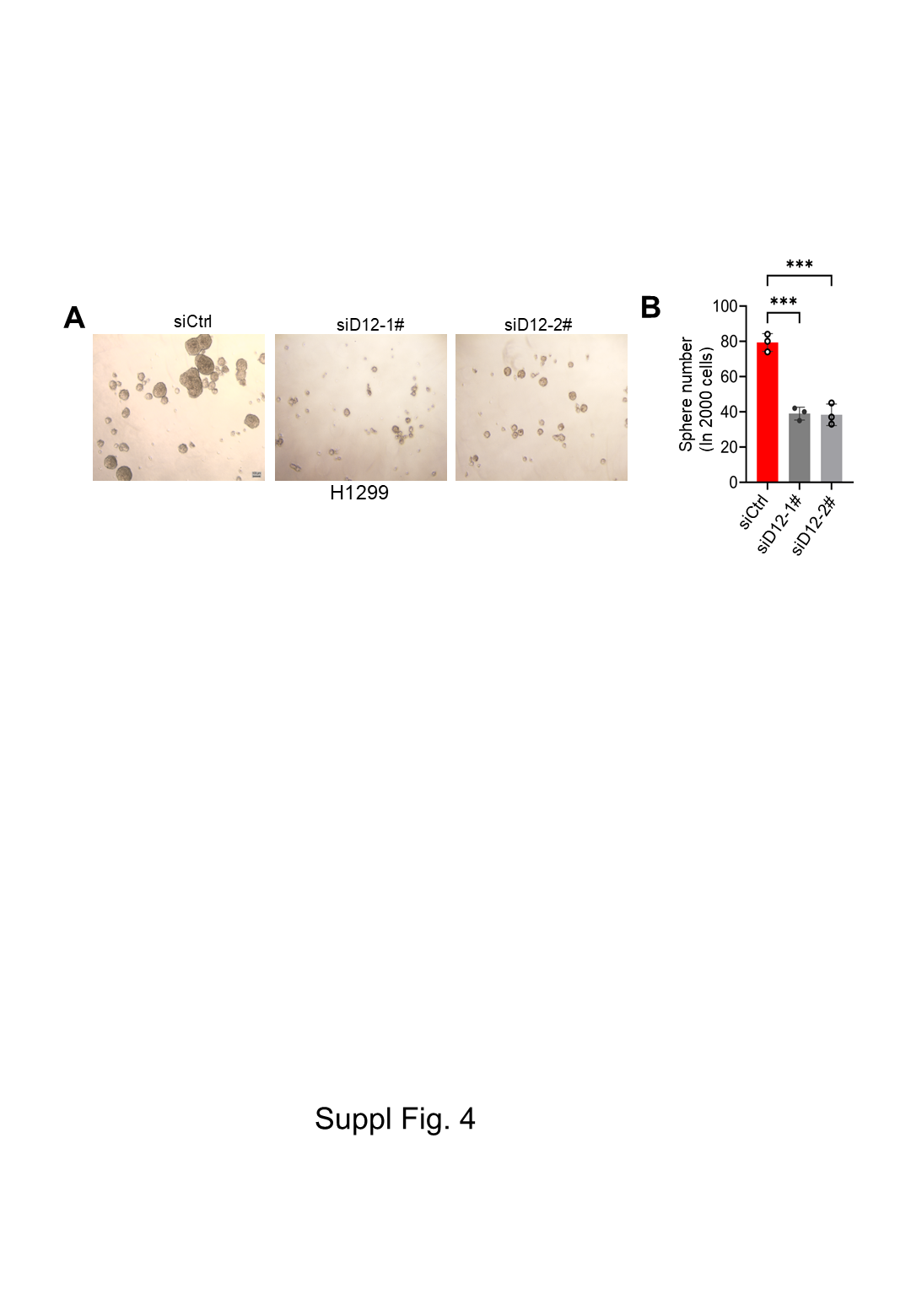
**

**Fig. S4. *DCAF12* knockdown inhibits sphere formation in H1299 cells.**

(**A**) H1299 cells transfected with either *DCAF12*-targeting siRNA or scrambled control siRNA were cultured under nonadherent conditions to assess their sphere-forming ability. After 10 days, tumor spheres were quantified by microscopic analysis. (**B**) Statistical analysis revealed a significant reduction in sphere formation upon DCAF12 depletion (mean ± SD, n = 3 biological replicates; one-way ANOVA with Tukey's post hoc test, *****P* < 0.0001).

**
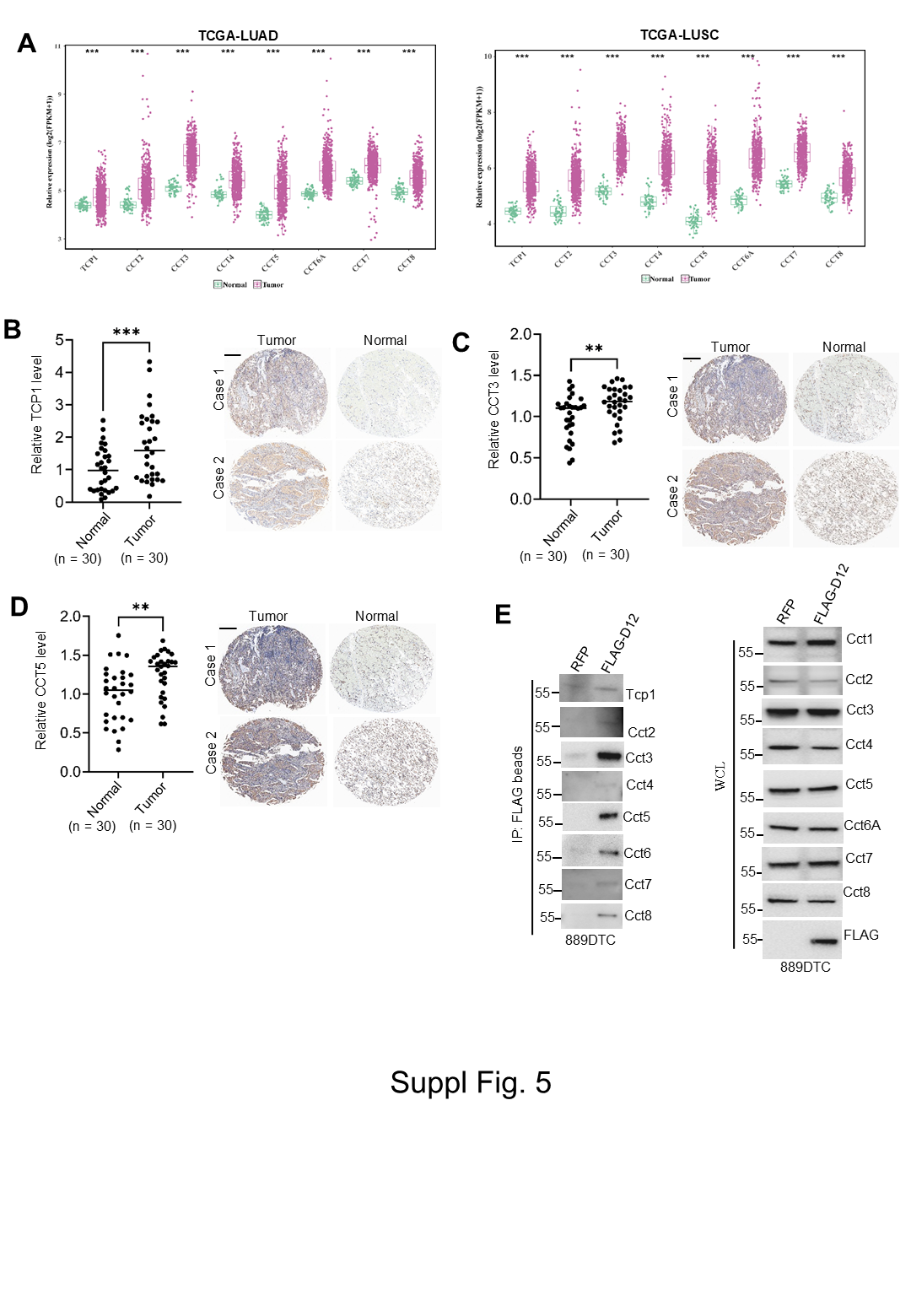
**

**Fig. S5. Expression profiles of TRiC/CCT subunits in lung cancer and their interactions with DCAF12.**(**A**) Comparative mRNA expression analysis of TRiC/CCT subunits between tumor tissues and matched normal adjacent tissues from TCGA datasets in LUAD (left) and LUSC (right).
(**B-D**) Representative IHC staining and quantitative analysis (H-score evaluation) of (B) TCP1, (C) CCT3, and (D) CCT5 protein expression in LUAD specimens and paired adjacent normal tissues from a 30-case tissue microarray cohort (mean ± SD, n = 30 paired samples; representative of one experiment; two-tailed paired Student's t-test, *P* < 0.01, **P* < 0.001).

(**E**)  Co-IP assays in 889DTC cells overexpressing FLAG-Dcaf12 (with 889DTC-RFP as control) demonstrated specific interactions with endogenous TRiC/CCT subunits (Tcp1, Cct2-8), as detected by immunoblotting.

**
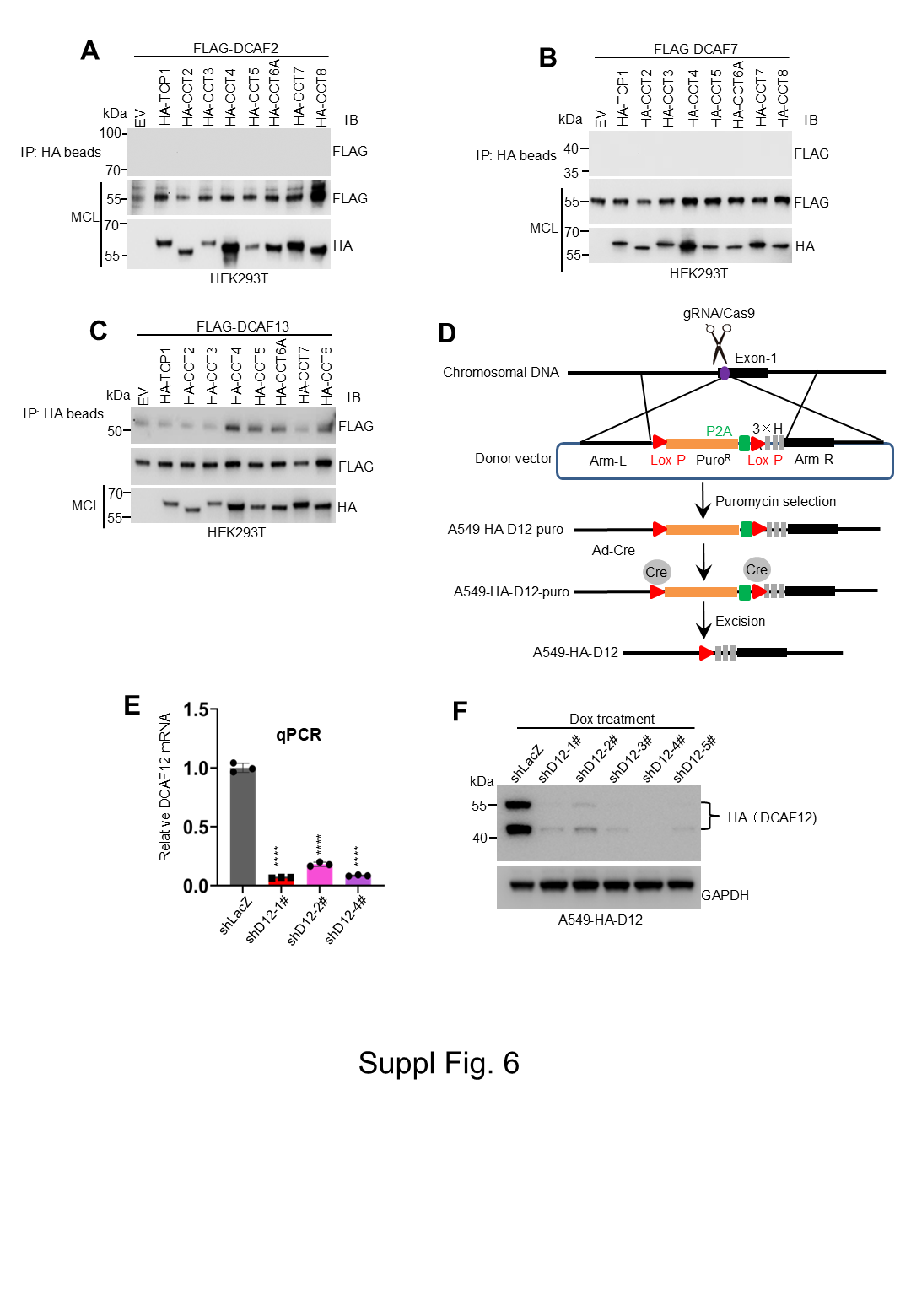
**

**Fig. S6. Endogenous HA-tagged DCAF12 in A549 cells using CRISPR/Cas9.**

(**A-C**) Co-IP assays revealed no or weak binding between the complete TRiC/CCT complex and DCAF2(A), DCAF7(B), or DCAF13 (C) when co-expressed in HEK293T cells.

(**D**) Schematic of the N-terminal HA-tag knock-in strategy at the *DCAF12* locus using gRNA/Cas9 with a repair template containing an HA tag and a floxed puromycin cassette.

(**E,F**) Validation of A549-HA-D12 cells with efficient *DCAF12* knockdown using qPCR (E) and immunoblotting (F). Data are presented as mean ± SD (n = 3 technical replicates per condition from one representative experiment of two independent biological replicates; one-way ANOVA with Tukey's multiple comparisons test, *****P* < 0.0001 vs. control).

**
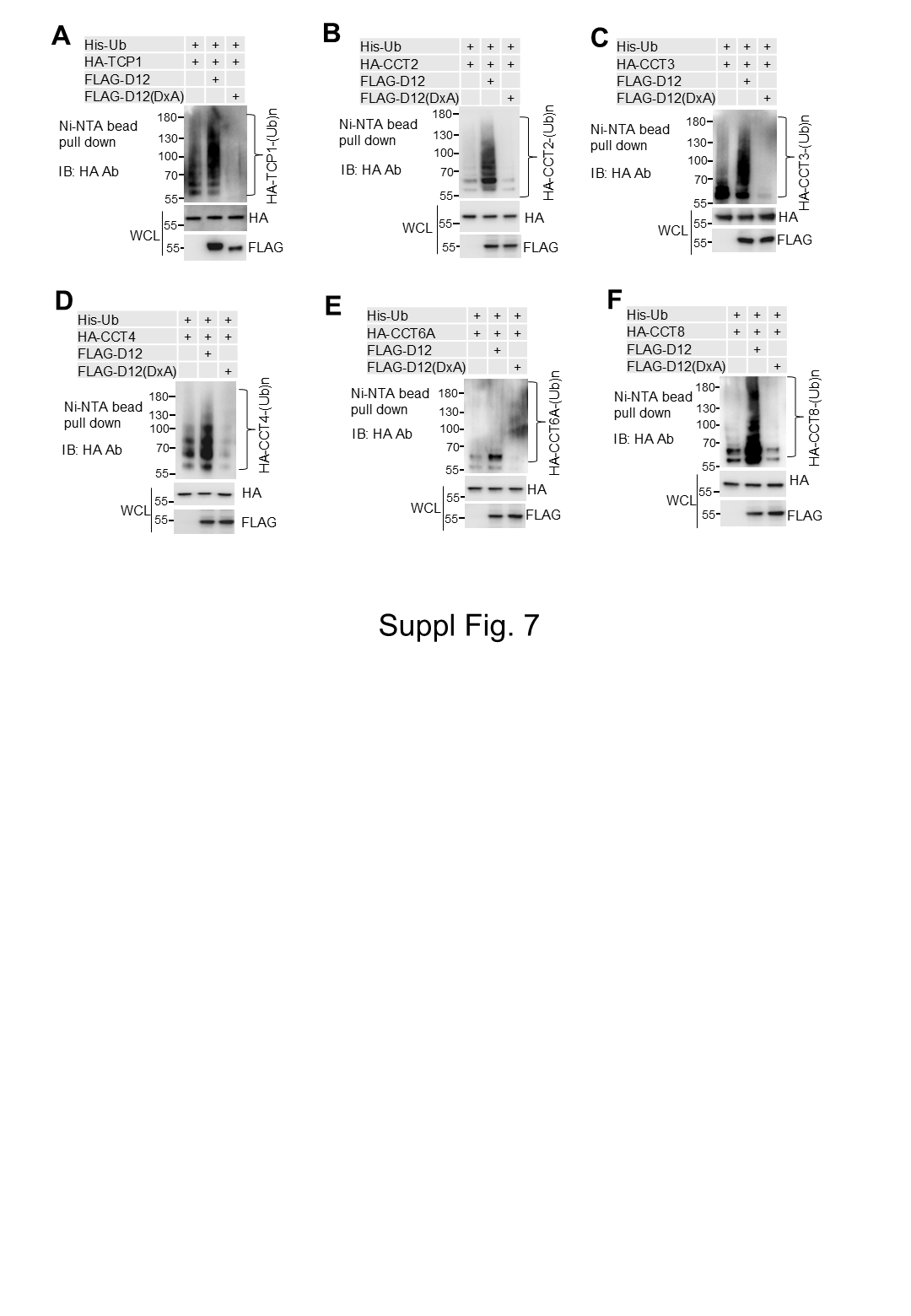
**

**Fig. S7. DCAF12 overexpression promotes ubiquitination of the TRiC/CCT subunits.**

HEK293T cells were co-transfected with either wild-type DCAF12 or the ubiquitination-defective DCAF12(DxA) mutant, together with plasmids encoding the indicated subunits. Ubiquitination assays were conducted 48 h after transfection to assess their effects on the ubiquitination of the following TRiC/CCT subunits: (A) TCP1, (B) CCT2, (C) CCT3, (D) CCT4, (E) CCT6A, and (F) CCT8.

**
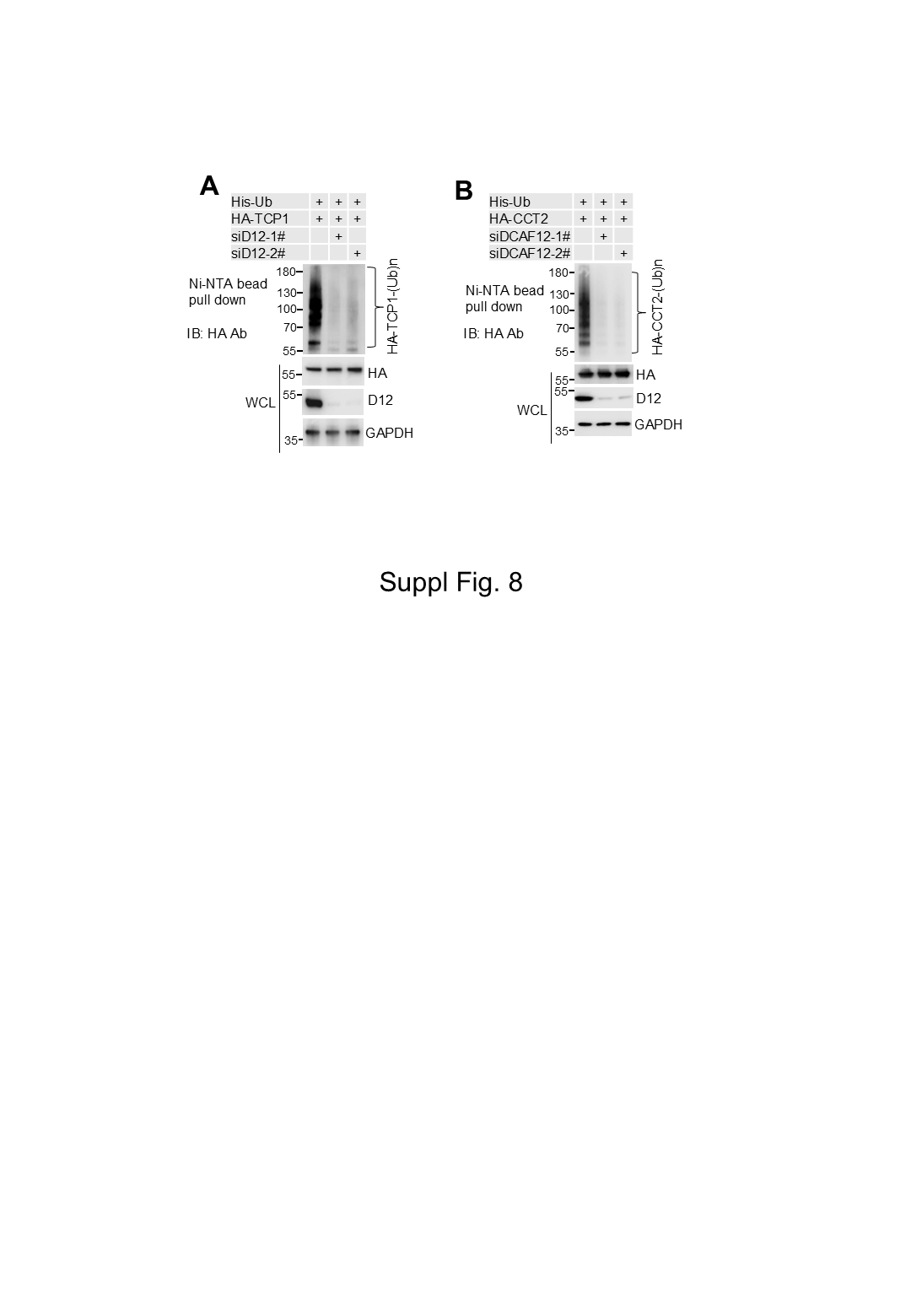
**

**Fig. S8. DCAF12 knockdown reduces ubiquitination of TCP1 and CCT2.**HEK293T cells were transfected with either DCAF12-targeting or control siRNA, followed by transfection with plasmids expressing TCP1 or CCT2. Ubiquitination assays performed 48 h after transfection showed that DCAF12 knockdown significantly decreased the ubiquitination of ectopically expressed TCP1 (A) and CCT2 (B) compared with the control group.

**
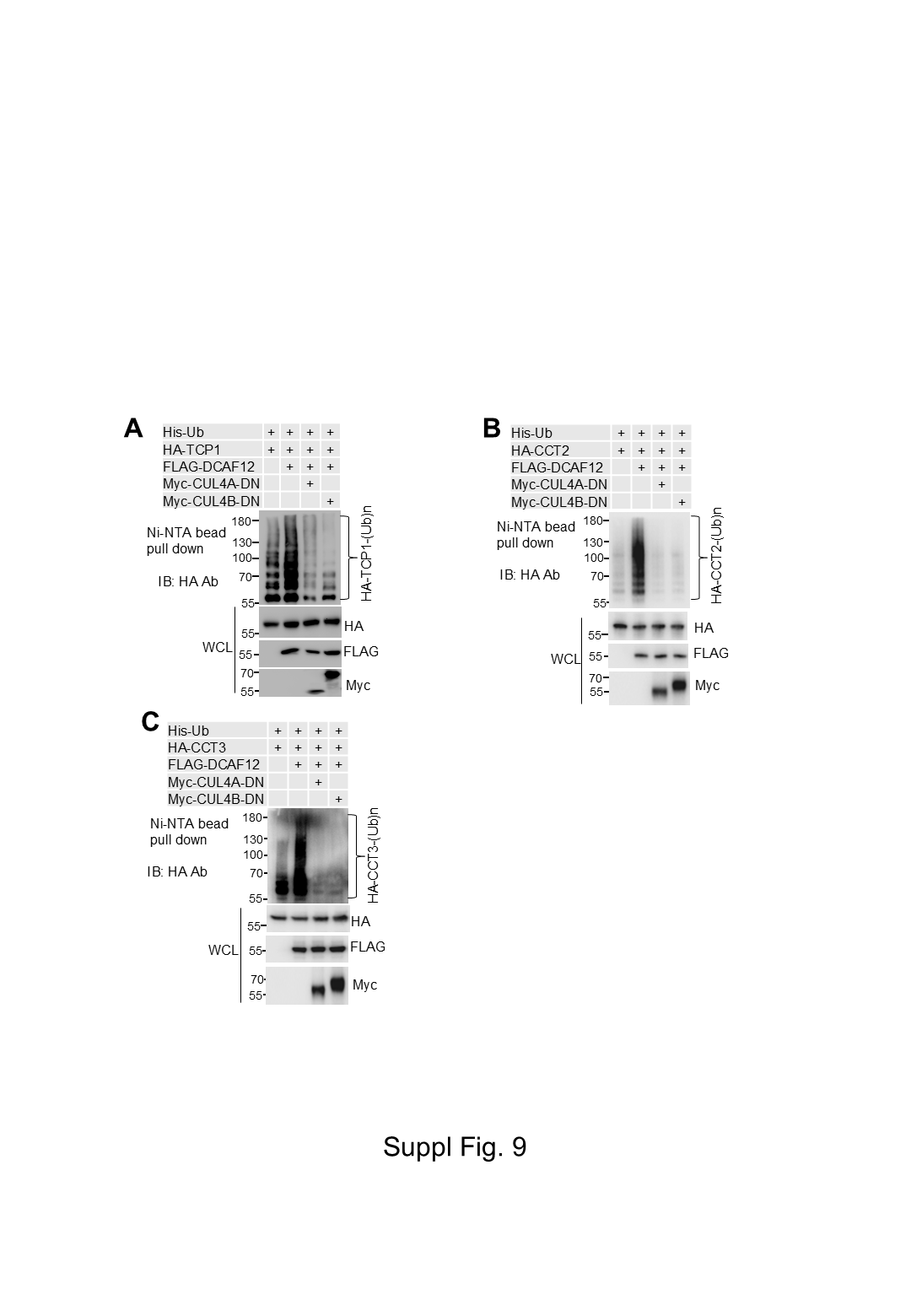
**

**Fig. S9. DCAF12 mediates TRiC/CCT subunit ubiquitination via CUL4-dependent mechanisms.** HEK293T cells were co-transfected with DCAF12 and either dominant-negative CUL4A (CUL4A-DN) or CUL4B (CUL4B-DN) mutants. Ubiquitination assays revealed significant reduction in TCP1(**A**), CCT2 (**B**), and CCT3 (**C**) ubiquitination compared to wild-type controls, demonstrating CRL4-DCAF12's essential role in these regulatory events.

**
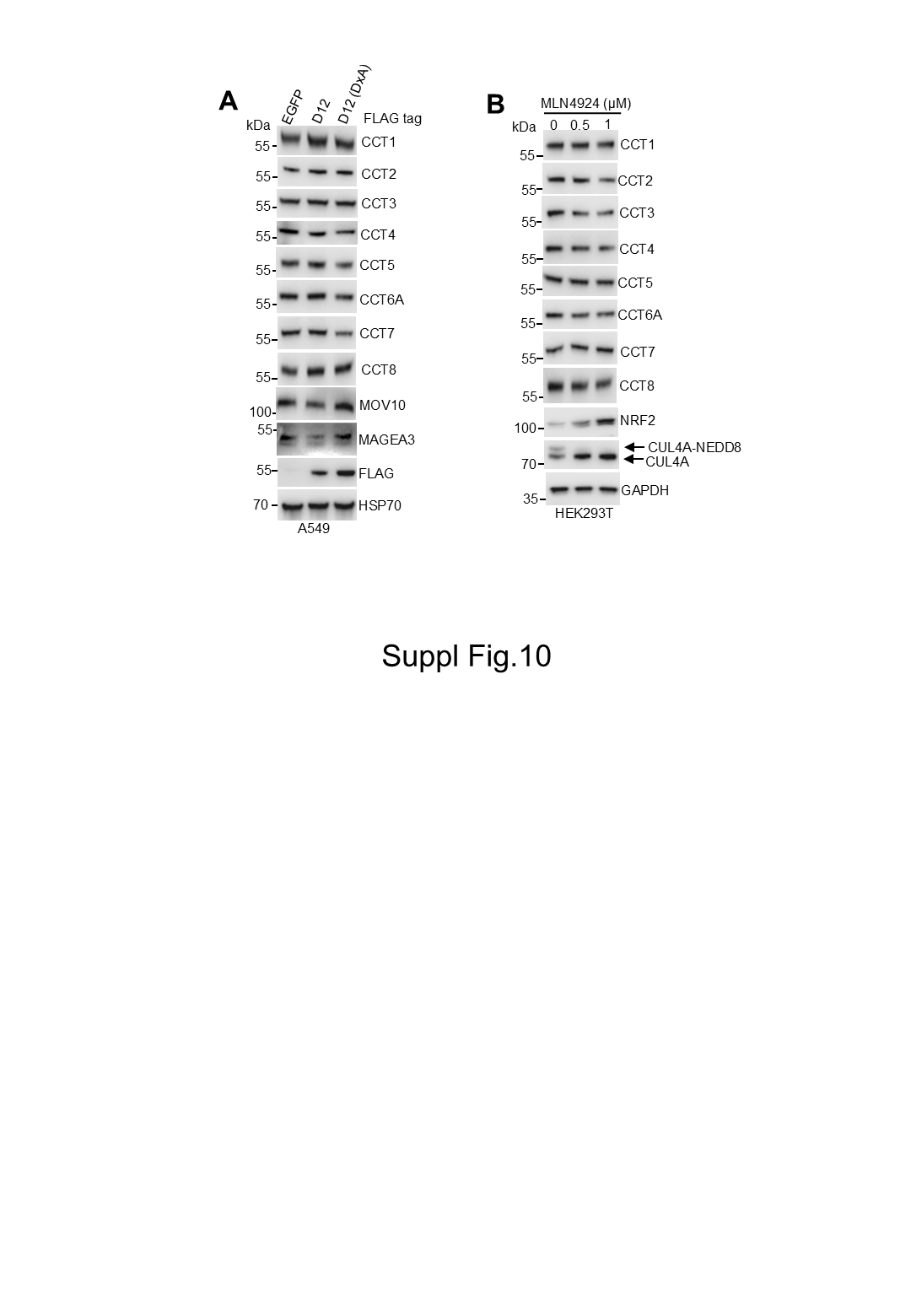
**

**Fig. S10. DCAF12 and neddylation regulate TRiC/CCT subunit stability.**

(**A**) Immunoblot analysis of TRiC/CCT subunit levels in HEK293T cells expressing wild-type DCAF12 or the CRL-binding-deficient mutant DCAF12(DxA). Antibodies against the indicated subunits demonstrated DCAF12-dependent stabilization.

(**B**) Immunoblot analysis of TRiC/CCT subunits after 24 h of treatment with the neddylation inhibitor MLN4924 (0, 0.5, and 1 μM), showing that neddylation inhibition slightly destabilized the complex. β-actin served as loading control.

**
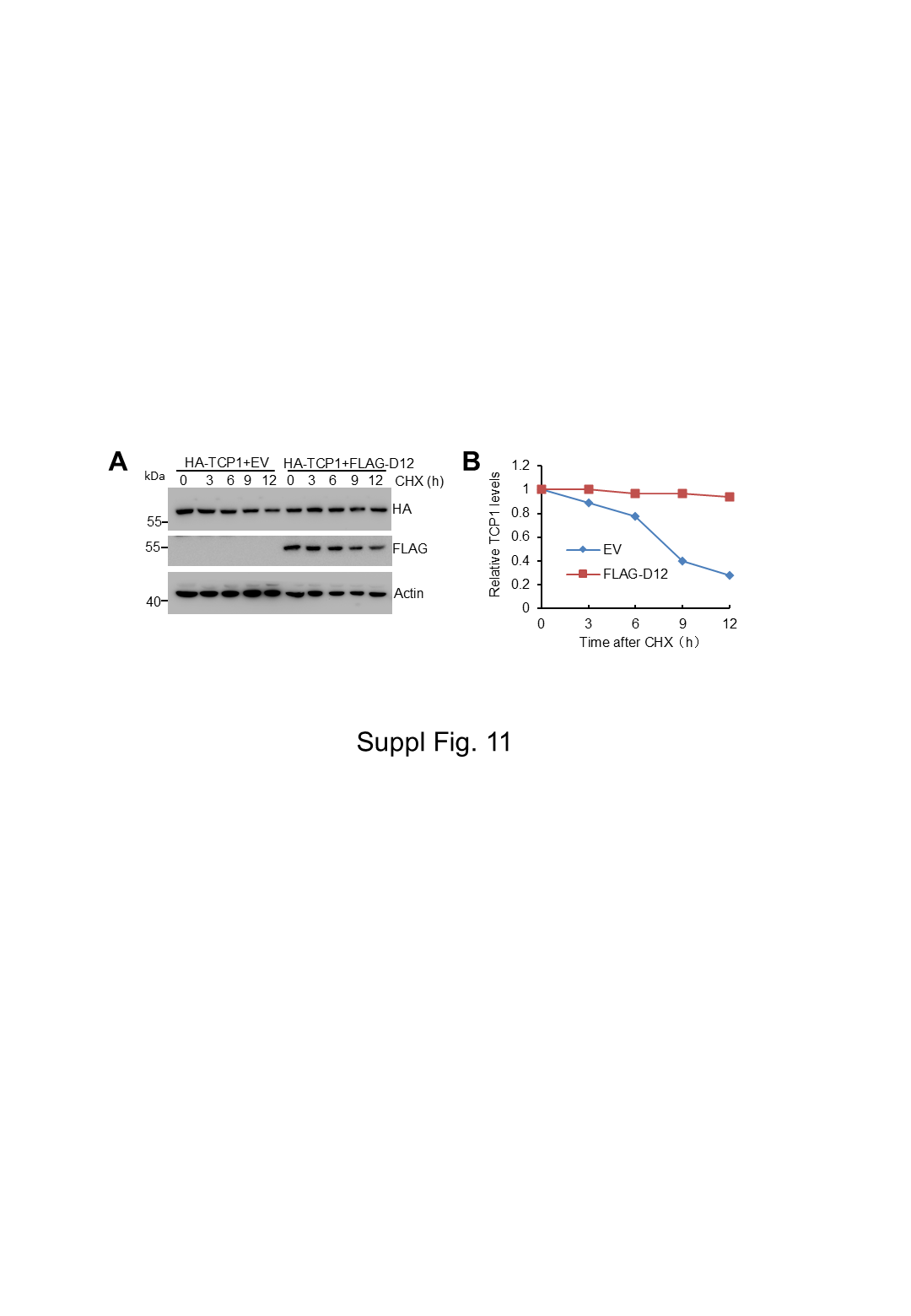
**

**Fig. S11. DCAF12 prolongs TCP1 half-life.**

(**A**)  CHX chase assay in HEK293T cells cotransfected with HA-TCP1 and either FLAG-DCAF12 or empty vector control for 48 h. Cells were treated with 100 μg/ml CHX and harvested at indicated time points (0, 3, 6, 9, and 12 h) for immunoblot analysis.

(**B**) Quantitative analysis of HA-TCP1 levels normalized to the loading control. Data shown are from one representative experiment (of two independent replicates), with band intensities quantified using the ImageJ software. The results demonstrated significant DCAF12-dependent stabilization of the TCP1 protein.

**
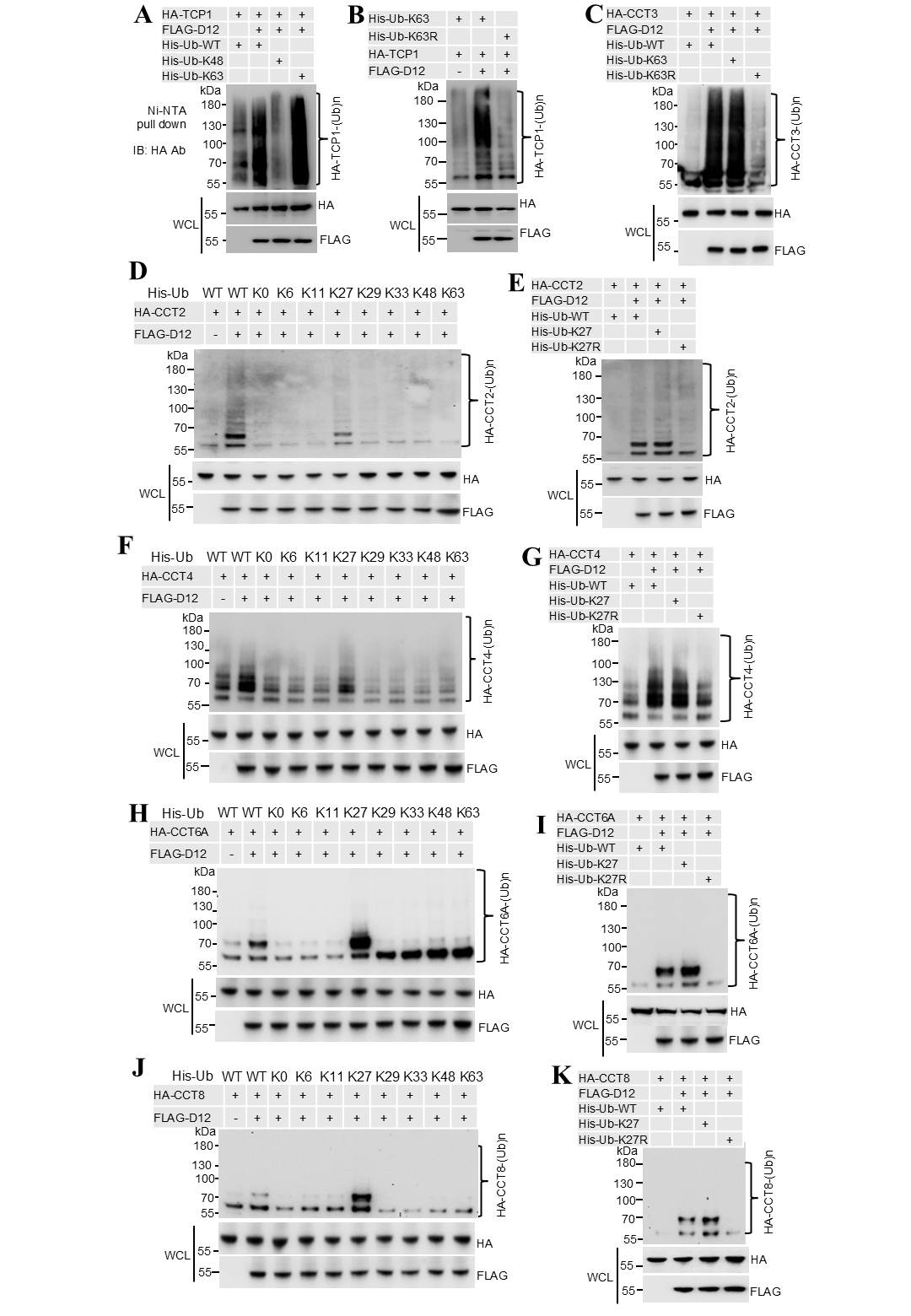
**

**Fig. S12. DCAF12 specifically facilitates K63- and K27-linked ubiquitination of the TRiC/CCT subunits.**(**A–C**) DCAF12 promotes K63-linked ubiquitination of TCP1 (A, B) and CCT3 (C) subunits. (**D–K**) DCAF12 facilitates K27-linked ubiquitination of CCT2 (D, E), CCT4 (F, G), CCT6A (H, I), and CCT8 (J, K) subunits.

**
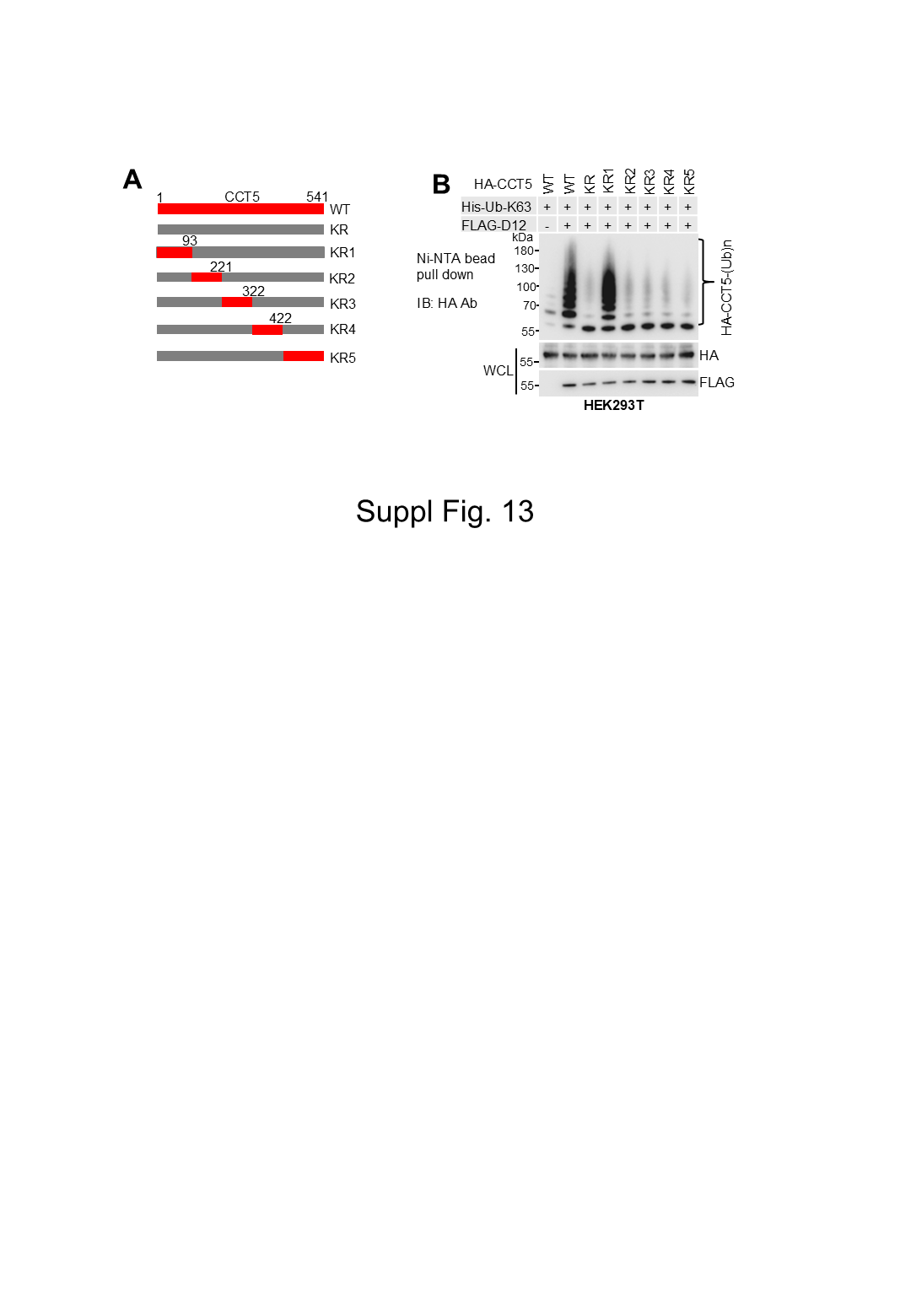
**

**Fig. S13. Identification of DCAF12-dependent ubiquitination sites on CCT5.**

 (**A**) CCT5 mutants were engineered to pinpoint DCAF12-mediated ubiquitination sites. A variant in which all lysines (K) were replaced with arginine (R) was called CCT5-KR. Overlapping mutants of CCT5-KR1 through CCT5-KR5 were created by PCR and seamless cloning.

 (**B**) An *in vivo* ubiquitination assay revealed that CCT5-KR1 is a site-specific target of ubiquitination upon DCAF12 overexpression.

**
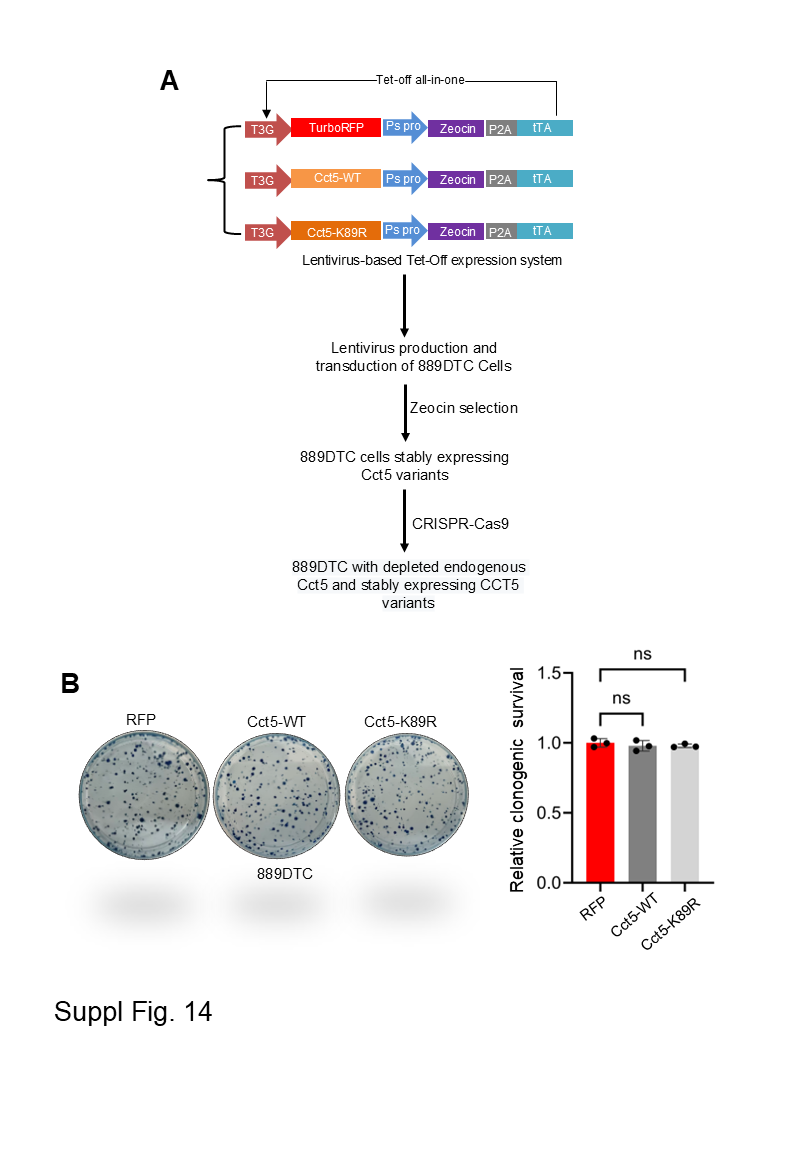
**

**Fig. S14. Workflow for generating the Cct5 functional rescue cell line model.**(**A**) Schematic diagram illustrating the establishment of the rescue cell line using a lentiviral tet-off system for inducible expression of either wild-type Cct5 or the K89R mutant. The procedure involved lentiviral packaging, transduction of target cells, selection with zeocin, and cell expansion. Endogenous Cct5 was subsequently knocked out using CRISPR-Cas9 to enable the reconstitution of the TRiC/CCT complex with exogenous wild-type or K89R mutant Cct5.

(**B**) The Cct5-K89R mutation has a minimal impact on the clonogenic survival of 889DTC cells.

**
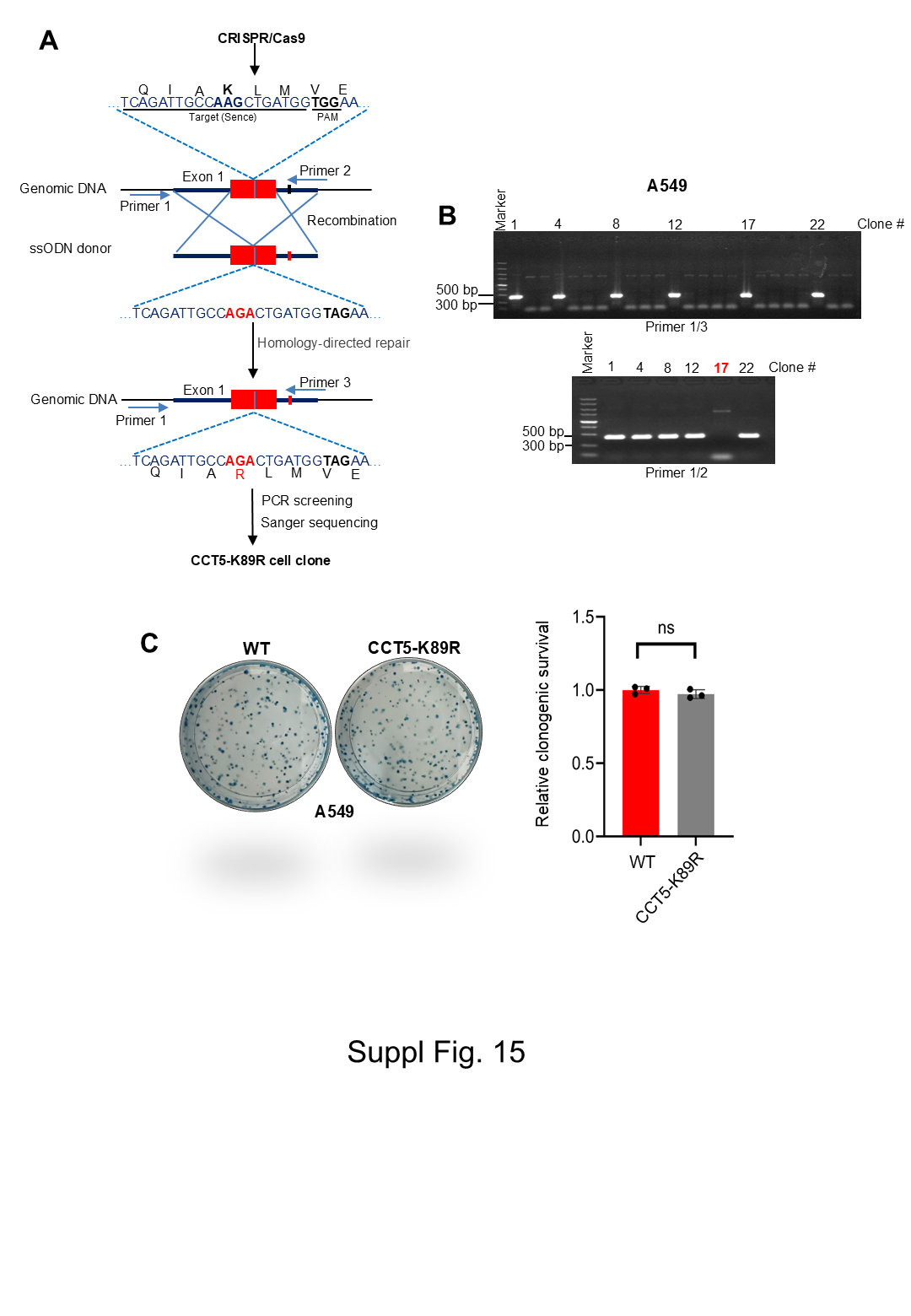
**

**Fig. S15. Generation of CCT5-K89R knock-in mutation in A549 cells.**(**A**) Strategy for CRISPR/Cas9-mediated K89R mutagenesis. The schematic depicts the sgRNA target site and homology-directed repair (HDR) template designed to introduce a lysine-to-arginine substitution at position 89 (K89R).

(**B**) Genotypic validation of the mutant clones. PCR amplification and agarose gel electrophoresis of the edited genomic region confirmed the successful introduction of homozygous K89R mutations using two independent primer sets, as shown in a representative clone (#17).

(**C**) CCT5-K89R mutation had a minimal effect on clonogenic survival in A549 cells.

**
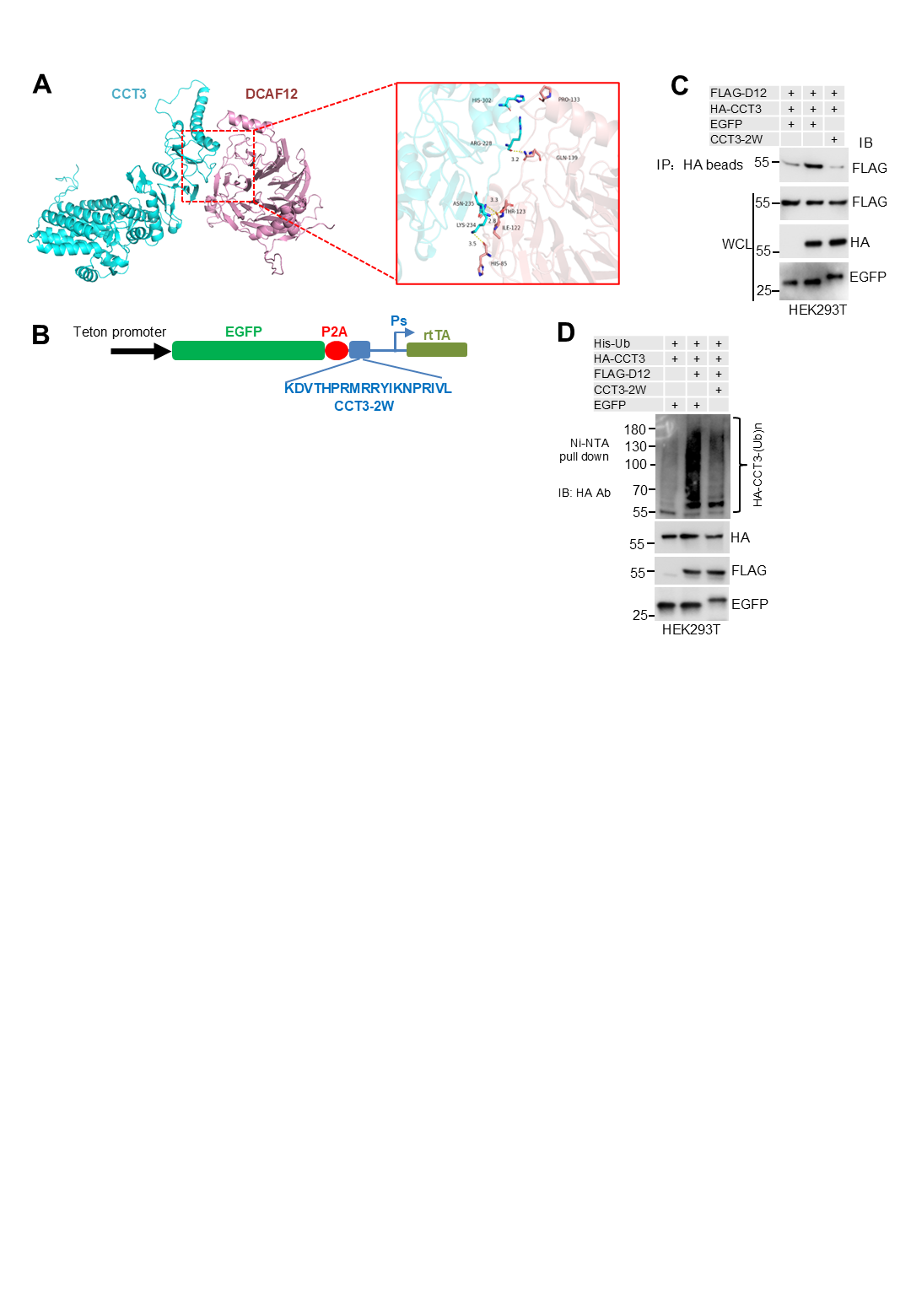
**

**Fig. S16. Design and validation of a CCT3 ubiquitination-blocking peptide**

(**A**) Structural prediction of the DCAF12-CCT3 interaction interface. Molecular docking analysis identified a putative binding interface between DCAF12 and CCT3, highlighting critical contact residues that mediate the interaction.

(**B**) Inducible expression of the CCT3-2W inhibitory peptide. Schematic of the Tet-On regulated bicistronic construct, where the CCT3-2W peptide is co-expressed with EGFP via a self-cleaving P2A peptide linker, enabling the stoichiometric production of both separate proteins.

(**C**) CCT3-2W specifically disrupted DCAF12-CCT3 binding. Co-IP assays demonstrated a significant reduction in DCAF12-CCT3 complex formation upon CCT3-2W overexpression compared to that in the control cells.

(**D**) CCT3-2W blocked CCT3 ubiquitination. His-ubiquitin pull-down assays revealed markedly decreased polyubiquitination of CCT3 in cells expressing the CCT3-2W peptide, relative to control conditions.

**
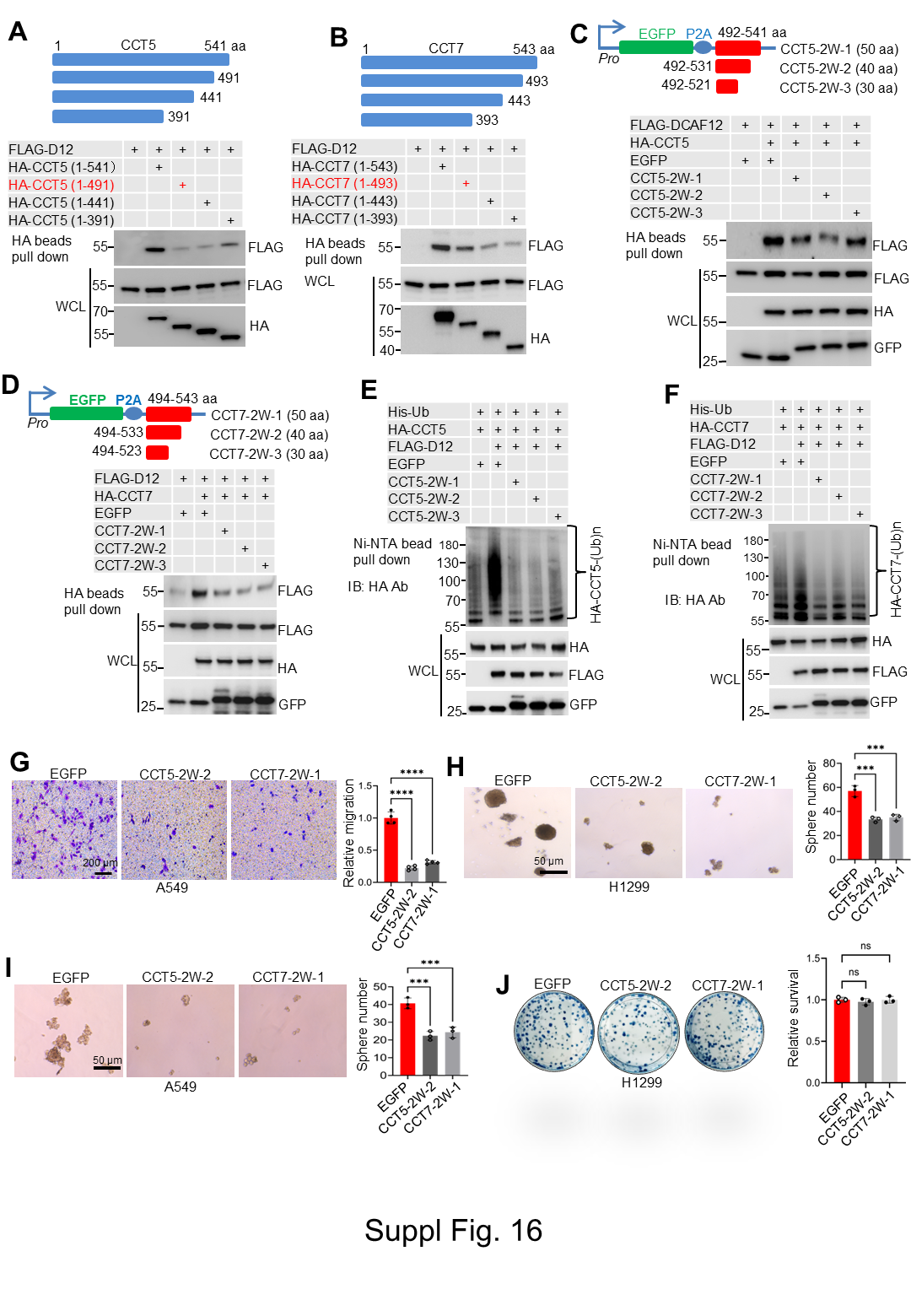
**

**Fig. S17. Peptide-based inhibition of DCAF12-mediated ubiquitination of TRiC/CCT components suppresses metastatic potential.**
(**A, B**) Co-IP analysis identified the essential C-terminal residues in CCT5 (A) and CCT7 (B) that are required for DCAF12 binding.
(**C, D**) Competitive inhibitory peptides that disrupt DCAF12–CCT5 (C) and DCAF12–CCT7 (D) interactions were identified by Co-IP. Peptides were expressed as EGFP fusions linked via a P2A self-cleaving motif under the Tet-On inducible control.
(**E, F**) In vivo ubiquitination assays confirmed that the inhibitory peptides effectively blocked the DCAF12-mediated ubiquitination of CCT5 (E) and CCT7 (F).
(**G**) Transwell migration assays showed that both CCT5-2W-2 and CCT7-2W-1 peptides significantly reduced A549 cell migration compared to the control group (n = 4 technical replicates per condition; data are from one representative experiment out of two independent repeats; one-way ANOVA with Tukey’s post-hoc test, ****P < 0.0001).
(**H, I)** Sphere formation assays revealed that both peptides markedly reduced tumor spheroid formation in H1299 (H) and A549 (I) cell lines compared to that in the control (n = 3 independent experiments; one-way ANOVA with Tukey’s post-hoc test, ***P < 0.001), indicating impaired cancer stem cell-like properties.
(**J**) Clonogenic survival assays demonstrated that both peptides had minimal cytotoxic effects in H1299 cells. Data from one representative experiment out of two independent replicates; one-way ANOVA with Tukey’s post-hoc test; ns: not significant.


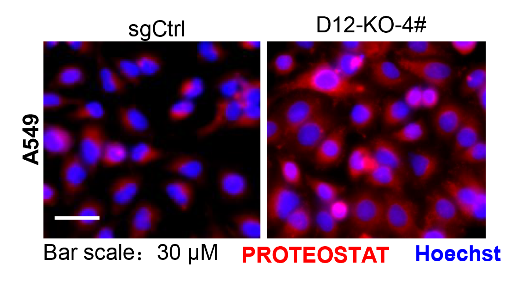


**Fig. S18. DCAF12 deficiency promotes protein aggregation in A549 cells**Representative fluorescence microscopy images of PROTEOSTAT (protein aggregates, red) and Hoechst 33342 (nuclei, blue) staining in control (sgCtrl) and DCAF12 knockout (D12-KO-4#) A549 cells.

**
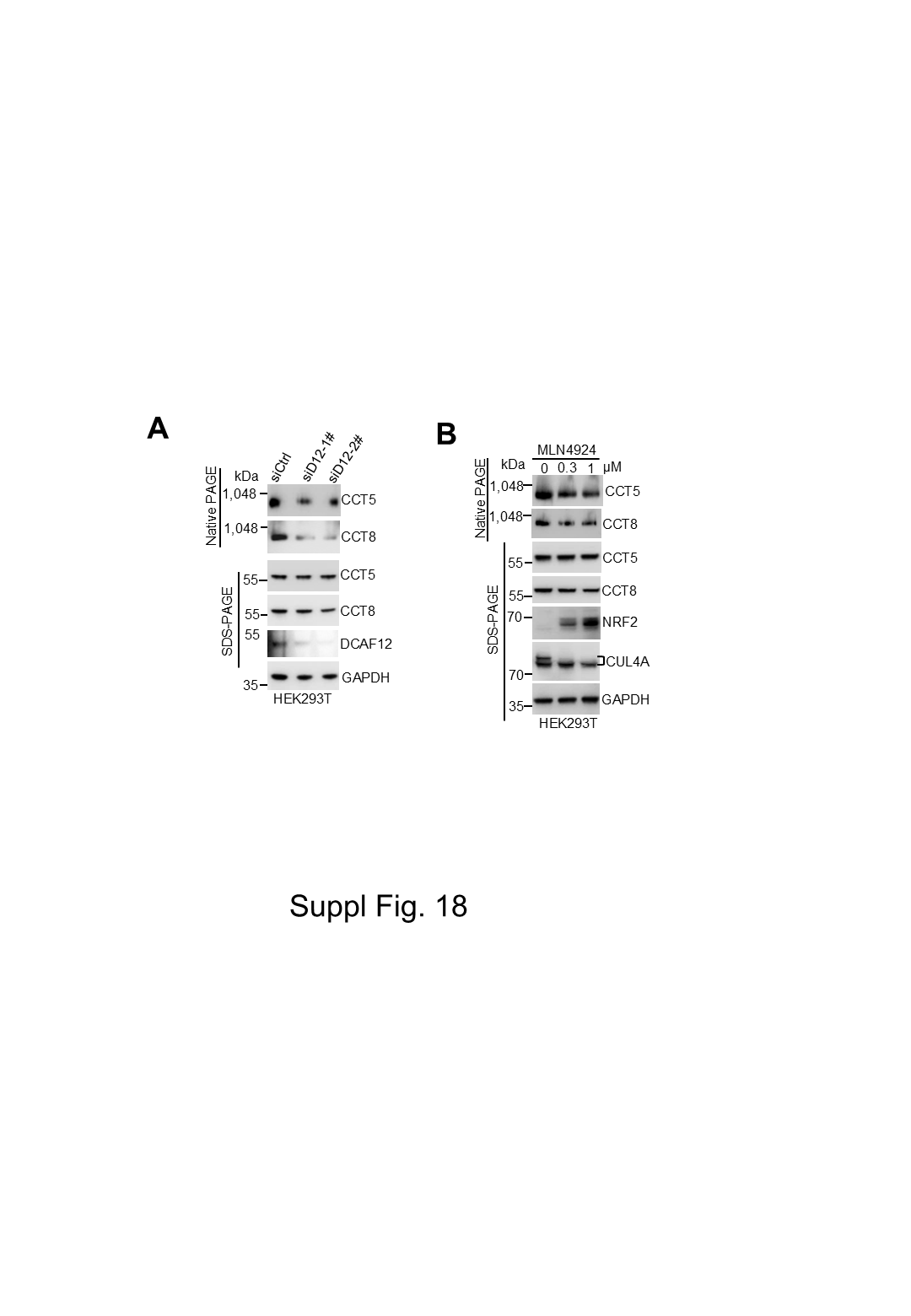
**

**Fig. S19. Native PAGE analysis of the TRiC/CCT complex assembly under DCAF12 depletion or neddylation inhibition.**

(**A**) Assessment of TRiC/CCT complex integrity in DCAF12-knockdown HEK293T cells. Native gel electrophoresis, followed by immunoblotting with anti-CCT5 and anti-CCT8 antibodies, revealed complex disruption. The total protein levels of CCT5 and CCT8 were verified by SDS-PAGE, with GAPDH serving as a loading control.

(**B**) Evaluation of the neddylation-dependent TRiC/CCT complex assembly in HEK293T cells. Cells were pretreated with MLN4924 (1 μM, 24 h) to inhibit neddylation before native gel electrophoresis. Complex formation was assessed by immunoblotting the CCT5 and CCT8 subunits.

**
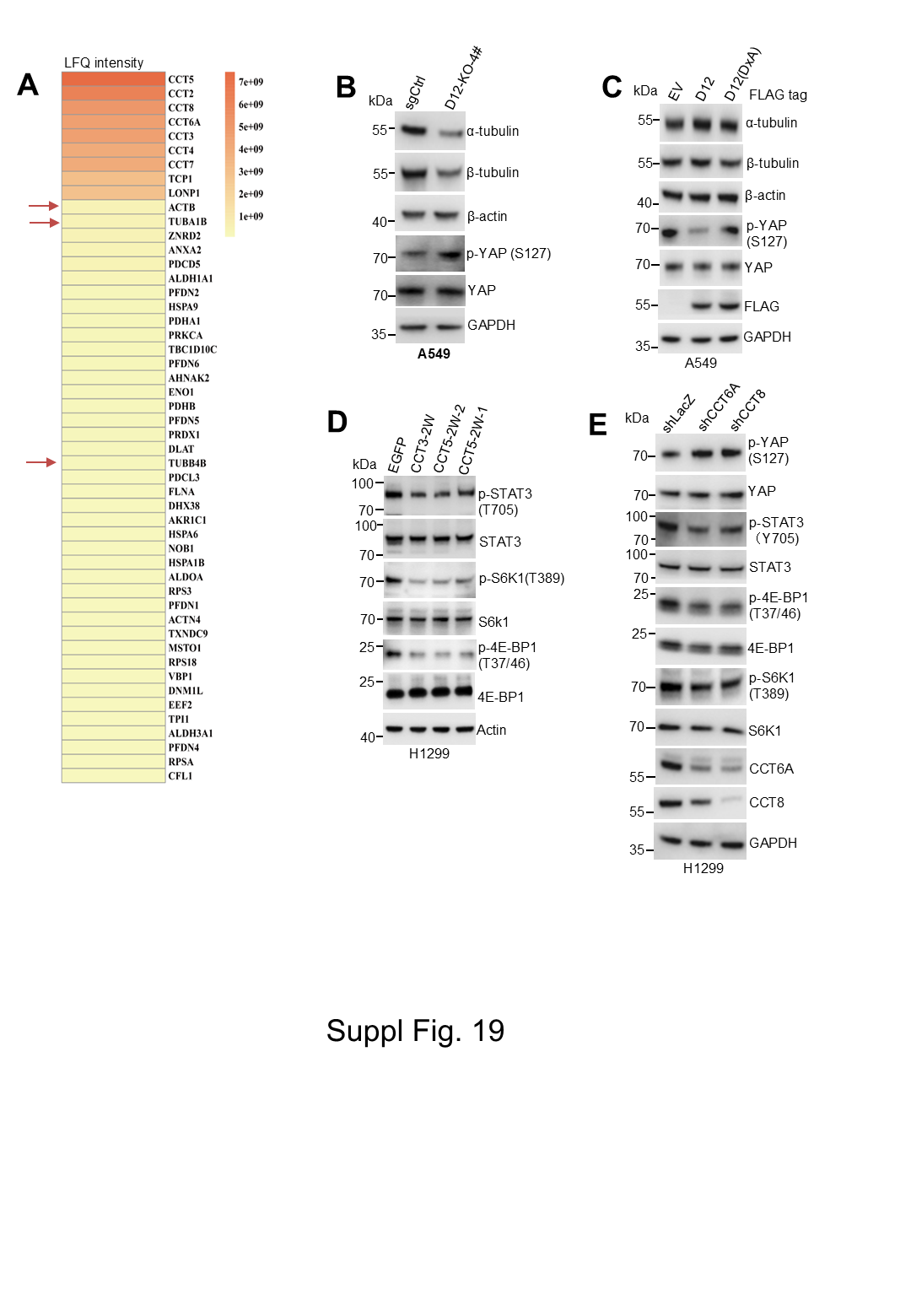
**

**Fig. S20. DCAF12 modulates cytoskeletal organization and oncogenic signaling through TRiC/CCT regulation**

(**A**) Quantitative proteomic profiling of CCT5-interacting proteins in A549 cells was performed using DSP cross-linking coupled with LC-MS/MS. The heatmap displays the top 50 interacting partners ranked by label-free quantification (LFQ) intensity, with prominent enrichment of cytoskeletal regulatory components (indicated by arrows).

(**B**) Genetic ablation of DCAF12 in A549 cells induced significant alterations in cytoskeletal protein networks and enhanced YAP phosphorylation at Ser127 compared to that in sgRNA control cells.

(**C**) Functional complementation assays demonstrated that wild-type DCAF12, but not its ubiquitination-defective DxA mutant, regulates both tubulin polymerization dynamics and YAP-S127 phosphorylation status in A549 cells.

(**D**) Pharmacological disruption of TRiC/CCT function using subunit-targeting inhibitory peptides suppressed the activation of key oncogenic signaling nodes, including 4EBP1 (Thr37/46), S6K1 (Thr389), and AKT (Ser473) in H1299 cells.

(**E**) Genetic perturbation of TRiC/CCT complex integrity through shRNA-mediated knockdown of either CCT6A or CCT8 subunits attenuated oncogenic pathway activation in H1299 cells.


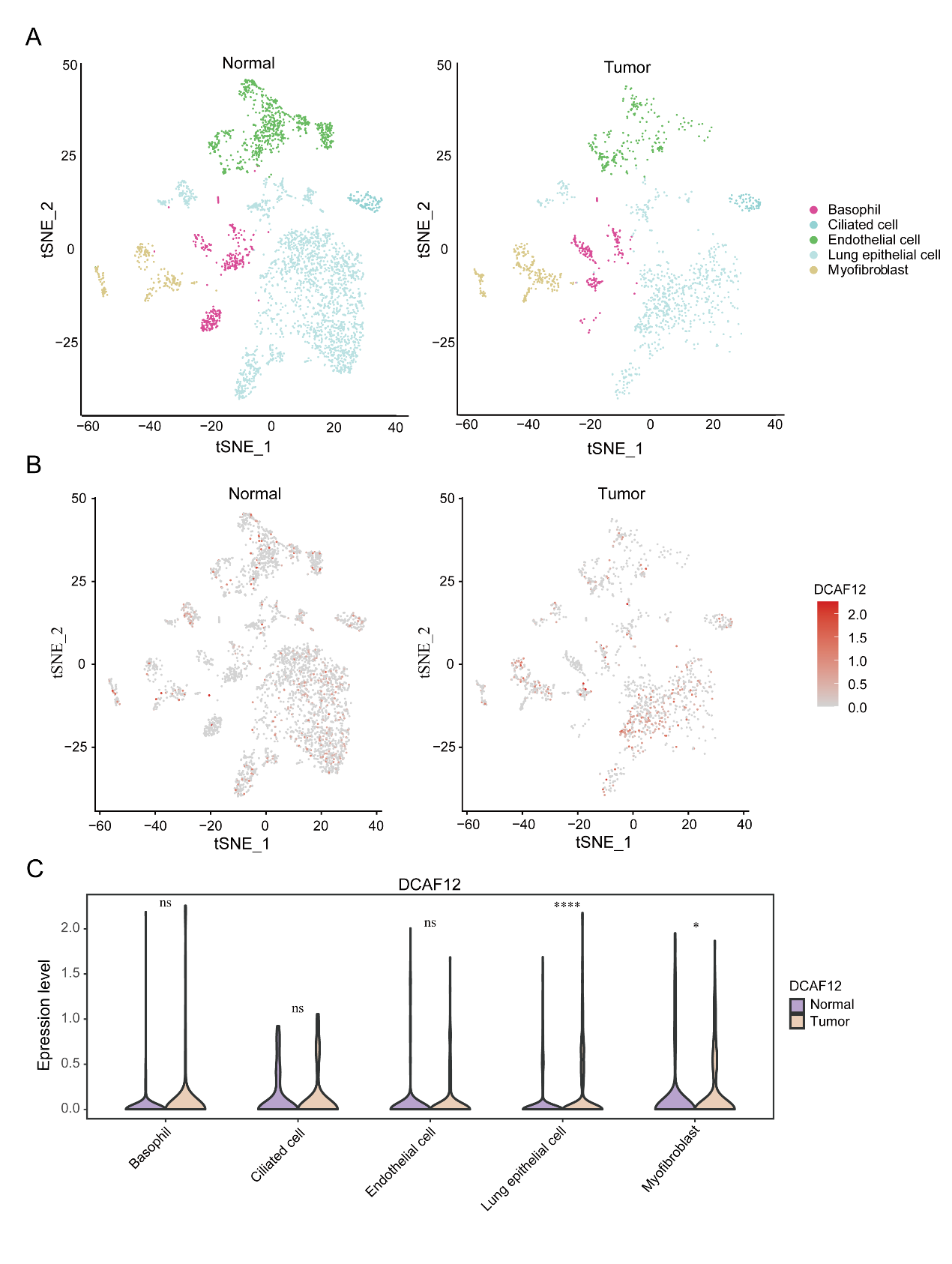


**Fig. S21. Single-cell RNA sequencing identified DCAF12 as a tumor-enriched marker in lung adenocarcinoma.**

(**A, B**) t-SNE projection of single-cell transcriptomes from the GSE149655 dataset revealed significant enrichment of DCAF12 expression in tumor cells from LUAD tissues compared to that in normal lung tissues.

(**C**) Violin plot quantification demonstrating significantly elevated DCAF12 expression levels in LUAD cells (**P* < 0.05; *****P* < 0.001; ns, not significant).


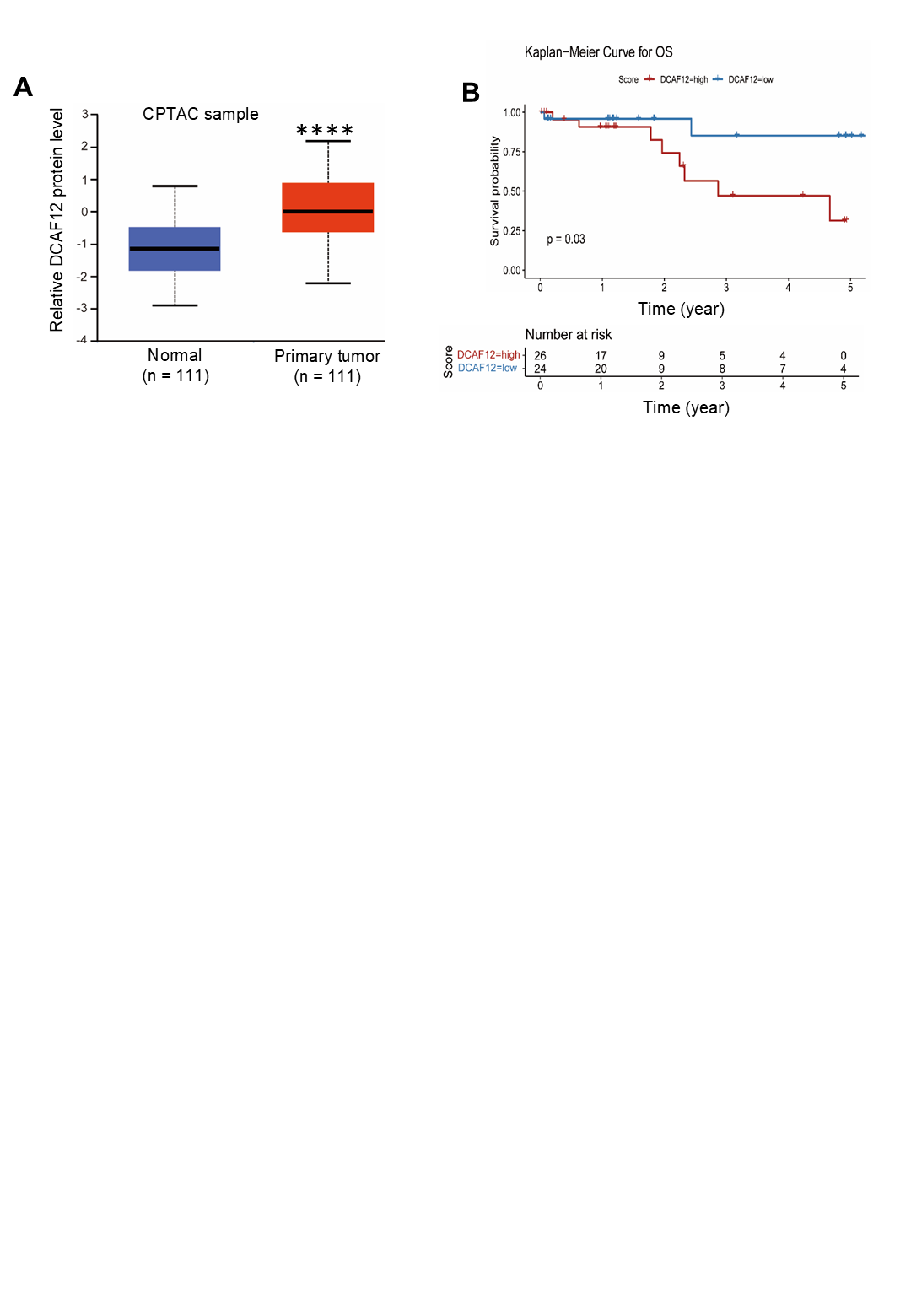


**Fig. S22. Elevated DCAF12 protein expression correlates with poor prognosis in LUAD.**(**A**) DCAF12 protein levels are significantly higher in LUAD tumors than in normal lung tissues in the CPTAC dataset. *****P* < 0.001.
(**B**) Kaplan–Meier analysis indicated that high DCAF12 expression predicted worse overall survival in patients with LUAD.

**Supplemental Experimental Procedures**

**Bioinformatic analysis**

**Transcriptomic Analysis**
Transcriptomic analysis was conducted using bulk RNA-sequencing data (in FPKM format) from The Cancer Genome Atlas (TCGA), including 503 LUAD samples with 54 matched normal tissues and 502 LUSC samples with 51 matched normal tissues. The data were log2(FPKM + 1) transformed before analysis. Differential expression of the complete DCAF gene family (DCAF1–DCAF17) and all CCT chaperonin genes was assessed by comparing tumor tissues with normal samples. A two-tailed Wilcoxon rank-sum test (implemented in R via the Wilcoxon test function) was applied, with an uncorrected significance threshold of p < 0.05.

**Single-Cell RNA Sequencing Analysis**
For higher-resolution expression profiling, single-cell RNA-seq data from two matched LUAD tumor–normal pairs (GSE149655, GEO) were analyzed using Seurat (v4.0). Quality control filtering was applied to retain genes detected in at least three cells and cells expressing between 250 and 7,500 genes with mitochondrial RNA content below 15% and more than 1,000 UMIs. After log-normalization and selection of the top 2,000 highly variable genes (variance-stabilizing transformation), batch effects were mitigated using canonical correlation analysis (CCA) integration. Dimensionality reduction was performed using principal component analysis (30 PCs), and cell clusters were identified using graph-based clustering (Louvain algorithm, resolution = 0.5), yielding five distinct cell populations. DCAF12 expression was visualized and compared between the tumor and normal tissues using the Wilcoxon rank-sum test.

**Proteomic and Survival Analysis**
To further investigate the role of DCAF12 in lung adenocarcinoma (LUAD), we assessed its protein expression and prognostic significance. Protein expression levels in LUAD and normal lung tissues were compared using the UALCAN online tool (<https://ualcan.path.uab.edu/index.html>), which leverages data from the Clinical Proteomic Tumor Analysis Consortium (CPTAC). For survival analysis, 50 patients with LUAD from the PCAS portal (<https://jingle.shinyapps.io/PCAS/>) were divided into high expression (n = 26) and low expression (n = 24) groups based on median DCAF12 expression. Kaplan–Meier survival curves were generated to compare the overall survival between the two groups and to evaluate the correlation of DCAF12 expression with clinical outcomes.

**CRISPRa-Mediated Transcriptional Activation of DCAF12**

Stable transcriptional activation of *DCAF12* was achieved in A549 non-small cell lung carcinoma cells using the CRISPR activation (CRISPRa) system. For lentiviral transduction, A549 cells were seeded in 6-well plates at a density of 2 × 10 cells per well (approximately 50% confluency). After 24 h, the cells were transduced with lentiviruses co-expressing dCas9-VPR transcriptional activator and sgRNAs targeting the *DCAF12* promoter.

Five sgRNAs (SgD12-1# to SgD12-5#) were designed using the Broad Institute’s CRISPick tool (<https://portals.broadinstitute.org/gpp/public/analysis-tools/sgrna-design>) to maximize specificity and on-target efficiency. Their sequences are as follows:
SgD12-1#: GGGGTTGGAAGATAGTTCTC
SgD12-2#: GTGCGGCTGCGCAAGGGTGA
SgD12-3#: GTTGTTTGGCTACTGCTGGA
SgD12-4#: GTGACGGCGCGCGAGCAAGG
SgD12-5#: TCCGAAGTGCGGCTGCGCAA

Non-targeting anti-EGFP sgRNA (GAAGGGCATCGACTTCAAGG) was used as a control. At 72 hours post-transduction, transduction efficiency was estimated to exceed 90% based on fluorescence observation.The activation efficiency was evaluated by qRT-PCR using SYBR Green Master Mix with specific primers for *DCAF12* (Forward: GAGCAGTGTCTCTGGATGGCTA; Reverse: AGCCAGACACACATTCTCACGG) and *GAPDH* (Forward: GTCTCCTCTGACTTCAACAGCG; Reverse: ACCACCCTGTTGCTGTAGCCAA) as the internal control. The relative gene expression levels were calculated using the 2^(− ΔΔCt) method.
