# Supplementary material for: DCAF12 Ubiquitin Ligase Promotes Lung Cancer Metastasis by Modulating the TRiC/CCT Chaperonin Complex
Source: Adv Sci (Weinh). 2025 Oct 5;13(3):e09695. doi: 10.1002/advs.202509695 (PMC12806248; doi:10.1002/advs.202509695)
Supplement: Supplementary file 2 — Supplementary Table 1 [file ADVS-13-e09695-s002.docx]

**Supplementary Table 1. Antibodies and reagents**

| **Antibodies** | **Source** | **Identifier** |
| --- | --- | --- |
| DCAF12 (for immunoblotting) | Abcam | ab106707 |
| DCAF12 (for immunoblotting and IHC) | Bioss | bs-9394R |
| Anti-FLAG M2 (mouse) | Sigma | F3165 |
| Anti-FLAG (Rabbit) | Proteintech | 20543-1-AP |
| TCP1/CCT1 | Proteintech | 10320-1-AP |
| Anti-CCT2 | Proteintech | 24896-1-AP |
| Anti-CCT3 | Proteintech | 10571-1-AP |
| Anti-CCT4 | Proteintech | 21524-1-AP |
| Anti-CCT5 | Proteintech | 11603-1-AP |
| Anti-CCT6A | Proteintech | 19793-1-AP |
| Anti-CCT7 | Proteintech | 15994-1-AP |
| Anti-CCT8 | Proteintech | 12263-1-AP |
| Anti-MOV10 | Proteintech | 10370-1-AP |
| Anti-MAGEA1 | Proteintech | 55105-1-AP |
| Anti-NRF2 | Proteintech | 16396-1-AP |
| Anti-CUL4A | Proteintech | 14851-1-AP |
| Anti-CUL4B | Proteintech | 12916-1-AP |
| Anti-DDB1 | Proteintech | 11380-1-AP |
| Anti-β-actin | Proteintech | 66009-1-Ig |
| Anti-α-tubulin | Proteintech | 11224-1-AP |
| Anti-β-tubulin | Proteintech | 10094-1-AP |
| Anti-GAPDH | Proteintech | 60004-1-Ig |
| Anti-HSP70 | Proteintech | 10995-1-AP |
| Anti-p21 | Proteintech | 10355-1-AP |
| Anti-NMI | Proteintech | 83986-2-RR |
| Anti-INCA1 | Proteintech | 30441-1-AP |
| Rabbit IgG control | Proteintech | 30000-0-AP |
| CoraLite488-conjugated Goat Anti-Rabbit IgG(H+L) | Proteintech | SA00013-2 |
| anti-HA | Cell Signaling Technology | 2367 |
| Anti-YAP | Cell Signaling Technology | 14074 |
| Anti-p-YAP (S127) | Cell Signaling Technology | 13008 |
| Anti-STAT3 | Cell Signaling Technology | 30835 |
| Anti-p-STAT3 (Y705) | Cell Signaling Technology | 9145 |
| Anti-4E-BP1 | Cell Signaling Technology | 9644 |
| Anti-p-4E-BP1 (T37/46) | Cell Signaling Technology | 2855 |
| Anti-AKT | Cell Signaling Technology | 9272 |
| Anti-AKT (S473) | Cell Signaling Technology | 4060 |
| Raptor | Cell Signaling Technology | 2280 |
| mLST8 | Cell Signaling Technology | 3274 |
| Anti-S6K1 | HUBIO | HA500095 |
| Anti-p-S6K1 (T389) | HUBIO | PSH02-23 |
|  |  |  |
| **Chemicals, beads, and kit** | **Source** | **Identifier** |
| Cycloheximide | Sigma | 1810 |
| MLN4924 | MedChemExpress | HY-70062 |
| HSF1A | MedChemExpress | HY-103000 |
| Phalloidin-Alexa Fluor 594 | Beyotime | C2205S |
| Hoechst 33342 | Beyotime | C1022 |
| anti-FLAG M2 magnetic beads | Sigma | M8823 |
| anti-HA magnetic beads | Thermo Fisher | PI88837 |
| Ni-NTA Agarose | QIAGEN | 30210 |
| Anti-GST Magnetic Beads | MedChemExpress | HY-K0222 |
| PROTEOSTAT Aggresome Detection Kit | Enzo Life Sciences | ENZ-51035-0025 |
| G-actin: F-actin In Vivo Assay Kit | Cytoskeleton | BK037 |
| **Recombinant DNA** | **Source** | **Identifier** |
| LT3GEPIR | Addgene | #111177 |
| Lenti-Tet-On::shDcaf12-1# | This paper | N/A |
| Lenti-Tet-On::shDcaf12-2# | This paper | N/A |
| Lenti-Tet-On::shDCAF12-1# | This paper | N/A |
| Lenti-Tet-On::shDCAF12-2# | This paper | N/A |
| Lenti-Tet-On::shDCAF12-3# | This paper | N/A |
| Lenti-Tet-On::shDCAF12-4# | This paper | N/A |
| Lenti-Tet-On::shDCAF12-5# | This paper | N/A |
| Lenti-Tet-On::shLacZ | This paper | N/A |
| Lenti-RFP | This paper | N/A |
| Lenti-FLAG-Dcaf12 | This paper | N/A |
| pcDNA3×FLAG-DCAF12 | This paper | N/A |
| pcDNA3×FLAG-DCAF12 (DxA) | This paper | N/A |
| Lenti-Tet-On::EGFP | This paper | N/A |
| Lenti-Tet-On:: FLAG-DCAF12 | This paper | N/A |
| Lenti-Tet-On:: FLAG- DCAF12 (DxA) | This paper | N/A |
| pcDNA3×HA-DCAF12 | This paper | N/A |
| pGEX-GST-TCP1 | This paper | N/A |
| pGEX-GST-CCT5 | This paper | N/A |
| pGEX-GST-CCT7 | This paper | N/A |
| pcDNA3×HA-TCP1 | This paper | N/A |
| pcDNA3×HA-CCT2-8 | This paper | N/A |
| pcDNA3×HA-DDB1 | This paper | N/A |
| pcDNA3×his-Ub | This paper | N/A |
| pcDNA3×his-Ub-K48 | This paper | N/A |
| pcDNA3×his-Ub-K63 | This paper | N/A |
| pcDNA3×his-Ub-K63R | This paper | N/A |
| pcDNA-myc-CUL4A-DN | This paper | N/A |
| pcDNA-myc-CUL4B-DN | This paper | N/A |
| pcDNA3×HA-CCT5-KR | This paper | N/A |
| pcDNA3×HA-CCT5-KR1-5 | This paper | N/A |
| pcDNA3×HA-CCT5-KR1 | This paper | N/A |
| pcDNA3×HA-CCT5-KR2 | This paper | N/A |
| pcDNA3×HA-CCT5-KR3 | This paper | N/A |
| pcDNA3×HA-CCT5-KR4 | This paper | N/A |
| pcDNA3×HA-CCT5-KR5 | This paper | N/A |
| pcDNA3×HA-CCT5-K20 | This paper | N/A |
| pcDNA3×HA-CCT5-K25 | This paper | N/A |
| pcDNA3×HA-CCT5-K35 | This paper | N/A |
| pcDNA3×HA-CCT5-K42 | This paper | N/A |
| pcDNA3×HA-CCT5-K59 | This paper | N/A |
| pcDNA3×HA-CCT5-K64 | This paper | N/A |
| pcDNA3×HA-CCT5-K89 | This paper | N/A |
| pcDNA3×HA-CCT5-K89R | This paper | N/A |
| pcDNA3×HA-Cct5-K89R | This paper | N/A |
| Tet-Off::RFP | This paper | N/A |
| Tet-Off::Cct5 | This paper | N/A |
| Tet-Off::Cct5-K89R | This paper | N/A |
| pLXsgRNA | Addgene | #50662 |
| [lentiCRISPRv2 puro](https://www.addgene.org/98290/) | Addgene | #98290 |
| Lenti-sgDCAF12 | This paper | N/A |
| Lenti-[sgCct5](https://www.addgene.org/107402/) | This paper | N/A |
| Lenti-[sgCtrl](https://www.addgene.org/107402/) | This paper | N/A |
| HA-CCT5 (1-491) | This paper | N/A |
| HA-CCT5 (1-441) | This paper | N/A |
| HA-CCT5 (1-391) | This paper | N/A |
| HA-CCT7 (1-493) | This paper | N/A |
| HA-CCT7 (1-443) | This paper | N/A |
| HA-CCT7 (1-393) | This paper | N/A |
| Lenti-EFS::EGFP-P2A-CCT5-2W-1 | This paper | N/A |
| Lenti-EFS::EGFP-P2A-CCT5-2W-2 | This paper | N/A |
| Lenti-EFS::EGFP-P2A-CCT5-2W-3 | This paper | N/A |
| Lenti-EFS::EGFP-P2A-CCT7-2W-1 | This paper | N/A |
| Lenti-EFS::EGFP-P2A-CCT7-2W-2 | This paper | N/A |
| Lenti-EFS::EGFP-P2A-CCT7-2W-3 | This paper | N/A |
| Lenti-EFS::EGFP | This paper | N/A |
| Lenti-Tet-On:: EGFP-P2A-CCT3-2W | This paper | N/A |
| Lenti-Tet-On:: EGFP-P2A-CCT5-2W-2 | This paper | N/A |
| Lenti-Tet-On:: EGFP-P2A-CCT7-2W-1 | This paper | N/A |
| pcDNA-EGFP | This paper | N/A |
| pcDNA-EGFP-P2A-TCP1 | This paper | N/A |
| pcDNA-EGFP-P2A-CCT2 | This paper | N/A |
| pcDNA-EGFP-P2A-CCT3 | This paper | N/A |
| pcDNA-EGFP-P2A-CCT4 | This paper | N/A |
| pcDNA-EGFP-P2A-CCT5 | This paper | N/A |
| pcDNA-EGFP-P2A-CCT6A | This paper | N/A |
| pcDNA-EGFP-P2A-CCT7 | This paper | N/A |
| pcDNA-EGFP-P2A-CCT8 | This paper | N/A |
| pcDNA3×HA-p53 | This paper | N/A |
| pcDNA3×HA-LATS1 | This paper | N/A |
| pcDNA3×HA-NF2 | This paper | N/A |
| pcDNA3×HA-PERP | This paper | N/A |
| pcDNA3×HA-p21 | This paper | N/A |
| pcDNA3×HA-p27 | This paper | N/A |
| Lenti-sgRNA/dCas9-VPR | This paper | N/A |
