# Supplementary material for: DCAF12 Ubiquitin Ligase Promotes Lung Cancer Metastasis by Modulating the TRiC/CCT Chaperonin Complex
Source: Adv Sci (Weinh). 2025 Oct 5;13(3):e09695. doi: 10.1002/advs.202509695 (PMC12806248; doi:10.1002/advs.202509695)
Supplement: Supplementary file 3 — Supplementary Table 2 [file ADVS-13-e09695-s001.docx]

**Supplementary Table 2.**

| Gene name | Gene ID | siRNA name | Sense (5′-3′) |
| --- | --- | --- | --- |
| *Dcaf2/Dtl* | *76843* | siDcaf2-1# | GAACGGGUGGUCUUCACAUUA |
|  |  | siDcaf2-2# | UGAUGAAGCUGCCUACAUUUG |
|  |  | siDcaf2-3# | GCACCAGCUGUCUAGUAUAUU |
| *Dcaf4* | 73828 | siDcaf4-1# | CCUUGAACGUCCAGGCAAAUA |
|  |  | siDcaf4-2# | CUUCUCCAGUUAUUGCCGUUU |
|  |  | siDcaf4-3# | CACUAAAUGUGUGAGACAGUA |
| *Dcaf7* | 71833 | siDcaf7-1# | CAACAACAAGAACUCAGACUU |
|  |  | siDcaf7-2# | CGCAUGUGUAUGACCUGACUU |
|  |  | siDcaf7-3# | GAUCGCUAUCUGCUACAACAA |
| *Dcaf12* | 245404 | siDcaf12-1# | GCGUGGAUCAACGAUACUAUG |
|  |  | siDcaf12-2# | AGUGGUGUGUGGCACGAAAUG |
|  |  | siDcaf12-3# | CCCUAACAGUCUUGCCAUCUA |
| *Dcaf13* | 223499 | siDcaf13-1# | CCCUGUUGAGACAUUUCUCUU |
|  |  | siDcaf13-2# | GCCAGCUUUGAUAAGUCUAUU |
|  |  | siDcaf13-3# | GAAAUCUAUCUACAGCCAGAU |
| *Dcaf15* | 212123 | siDcaf15-1# | UUACCCUGGACUUCGAGUAUG |
|  |  | siDcaf15-2# | GAGAGUCUGGAUGUGUCUUUA |
|  |  | siDcaf15-3# | ACGACUUCUCCUUCUACAUAU |
| *Dcaf17* | 75763 | siDcaf17-1# | AUAAUUGGCUGCUUCGUAUAU |
|  |  | siDcaf17-2# | UCCUGACCCAUCAGACUAUAA |
|  |  | siDcaf17-3# | ACUACAUCAUCACGCCUAAUA |
|  |  | siControl | GGCUACGUCCAGGAGCGCACC |
|  | | | |
| Gene name | Gene ID | shRNA name | 97-mer oligonucleotides （Full Hairpin Sequence） |
| *Dcaf12* | *245404* | shDcaf12-1# | TGCTGTTGACAGTGAGCGAACAGGGATATTAAGTACATAATAGTGAAGCCACAGATGTATTATGTACTTAATATCCCTGTGTGCCTACTGCCTCGGA |
|  |  | shDcaf12-2# | TGCTGTTGACAGTGAGCGCGAGTGTTGAAGAGTAATCCAATAGTGAAGCCACAGATGTATTGGATTACTCTTCAACACTCATGCCTACTGCCTCGGA |
| *DCAF12* | 139170 | ShDCAF12-1# | TGCTGTTGACAGTGAGCGCTCATACATTCTGTTTCTTTAATAGTGAAGCCACAGATGTATTAAAGAAACAGAATGTATGAATGCCTACTGCCTCGGA |
|  |  | ShDCAF12-2# | TGCTGTTGACAGTGAGCGCTCCGAACAACTTAATATAATATAGTGAAGCCACAGATGTATATTATATTAAGTTGTTCGGATTGCCTACTGCCTCGGA |
|  |  | ShDCAF12-3# | TGCTGTTGACAGTGAGCGCCCGAACAACTTAATATAATAATAGTGAAGCCACAGATGTATTATTATATTAAGTTGTTCGGATGCCTACTGCCTCGGA |
|  |  | ShDCAF12-4# | TGCTGTTGACAGTGAGCGCCCCAGGATTAGTAATTTGTTATAGTGAAGCCACAGATGTATAACAAATTACTAATCCTGGGTTGCCTACTGCCTCGGA |
|  |  | ShDCAF12-5# | TGCTGTTGACAGTGAGCGACACTAATACTTAGCAAATAAATAGTGAAGCCACAGATGTATTTATTTGCTAAGTATTAGTGGTGCCTACTGCCTCGGA |
| CCT6A | 908 | ShCCT6A | TGCTGTTGACAGTGAGCGCGGAGATGAAACATAAATCTGATAGTGAAGCCACAGATGTATCAGATTTATGTTTCATCTCCATGCCTACTGCCTCGGA |
| CCT8 | 150160 | ShCCT8 | TGCTGTTGACAGTGAGCGAAAGCGATCAATTACTAGAAAATAGTGAAGCCACAGATGTATTTTCTAGTAATTGATCGCTTCTGCCTACTGCCTCGGA |
|  |  |  |  |
| Gene name | Gene ID | gRNA name | gRNA sequence |
| *DCAF12* | 139170 | DCAF12-gRNA | 5′ -CATCTCAGTGGTTGAATCAT-3′ |
|  |  | Control-gRNA | 5′-GGGCGAGGAGCTGTTCACCG-3′ |
